# Novel core promoter elements in the oomycete pathogen *Phytophthora infestans* and their influence on expression detected by genome-wide analysis

**DOI:** 10.1186/1471-2164-14-106

**Published:** 2013-02-16

**Authors:** Sourav Roy, Laetitia Poidevin, Tao Jiang, Howard S Judelson

**Affiliations:** 1 Department of Plant Pathology and Microbiology, University of California, 92521, Riverside, CA, USA; 2 Department of Computer Science, University of California, 92521, Riverside, CA, USA

**Keywords:** Core promoter, Transcription initiation, Oomycete genome, Promoter mutagenesis, Transcription factor binding site, Reporter gene assay

## Abstract

**Background:**

The core promoter is the region flanking the transcription start site (TSS) that directs formation of the pre-initiation complex. Core promoters have been studied intensively in mammals and yeast, but not in more diverse eukaryotes. Here we investigate core promoters in oomycetes, a group within the Stramenopile kingdom that includes important plant and animal pathogens. Prior studies of a small collection of genes proposed that oomycete core promoters contain a 16 to 19 nt motif bearing an Initiator-like sequence (INR) flanked by a novel sequence named FPR, but this has not been extended to whole-genome analysis.

**Results:**

We used expectation maximization to find over-represented motifs near TSSs of *Phytophthora infestans,* the potato blight pathogen. The motifs corresponded to INR, FPR, and a new element found about 25 nt downstream of the TSS called DPEP. TATA boxes were not detected. Assays of DPEP function by mutagenesis were consistent with its role as a core motif. Genome-wide searches found a well-conserved combined INR+FPR in only about 13% of genes after correcting for false discovery, which contradicted prior reports that INR and FPR are found together in most genes. INR or FPR were found alone near TSSs in 18% and 7% of genes, respectively. Promoters lacking the motifs had pyrimidine-rich regions near the TSS. The combined INR+FPR motif was linked to higher than average mRNA levels, developmentally-regulated transcription, and functions related to plant infection, while DPEP and FPR were over-represented in constitutively-expressed genes. The INR, FPR, and combined INR+FPR motifs were detected in other oomycetes including *Hyaloperonospora arabidopsidis, Phytophthora sojae, Pythium ultimum,* and *Saprolegnia parasitica,* while DPEP was found in all but *S. parasitica.* Only INR seemed present in a non-oomycete stramenopile.

**Conclusions:**

The absence of a TATA box and presence of novel motifs show that the oomycete core promoter is diverged from that of model systems, and likely explains the lack of activity of non-oomycete promoters in *Phytophthora* transformants. The association of the INR+FPR motif with developmentally-regulated genes shows that oomycete core elements influence stage-specific transcription in addition to regulating formation of the pre-initiation complex.

## Background

Growth, development, and responses to environmental signals require the proper level and timing of transcription. In eukaryotes, the DNA binding sites for the transcription machinery are named enhancers, proximal or distal elements, or core promoter motifs depending on their location and function [1]. The core promoter is normally defined as the 50 bases on either side of the transcription start site (TSS), which contain sites that help position RNA polymerase during establishment of the preinitiation complex [2]. It has recently been recognized that core promoter elements also influence tissue-specific transcription [3]. Interactions at the core promoter thus help ensure that transcription is efficient, regulated, and fine-tuned.

Most of what is known about core promoters comes from yeast and animals [2]. The first discovered core motif was the TATA box, which lies about 30 nt upstream of the TSS in mammals and binds TFIID, which contains TATA-binding protein (TBP). Other motifs revealed later include Initiator (INR) and downstream promoter element (DPE), which also bind TFIID, and TFIIB recognition elements (BREs). Computational approaches enabled by whole-genome data then identified further motifs, and a strong majority of human and mouse promoters are now recognized to contain known core elements [4]. Although yeast and animals both belong to the kingdom Opisthokonta, there is ample evidence for diversification of their core promoters: only some motifs such as TATA and INR are well-conserved, pre-initiation complexes assemble much further upstream in yeast than mammals, and sequence differences within their TBPs affect promoter recognition and the binding of other transcription factors [5].

In other eukaryotic kingdoms, the nature of core promoters is just starting to be understood. Most is known from plants, where *Arabidopsis* and rice were shown to use a TATA box, INR, and plant-specific motifs such as the pyrimidine-rich Y-patch [6,7]. In the kingdom Excavata, *Trichomonas* appears to employ an INR and two novel elements but not a TATA box, while *Plasmodium* core promoters lack any defined sequence element. In the latter, initiation sites appear to be determined by the physicochemical properties of DNA, which may also play roles in all eukaryotes [8,9].

This paper focuses on core promoter elements in oomycetes, a group within the kingdom Stramenopila that includes many important plant and animal pathogens. Although most oomycetes superficially appear fungal-like, they contain many characteristics distinct from fungi which is consistent with the divergence of stramenopiles from opisthokonts early in the eukaryotic radiation [10]. Little is known about core promoters in any stramenopile. Plant, animal, and fungal promoters were shown to work poorly in the potato late blight pathogen *Phytophthora infestans,* which suggested divergence of the transcriptional apparatus of oomycetes [11]. Researchers examining the few genes that were available prior to the development of whole-genome sequences reported that nearly all oomycete promoters contain a 16 to 19 nt region near the TSS, which contained a 7 nt INR-like element at its 5^′^ end followed by an approximately 9 nt sequence named Flanking Promoter Region or FPR [12,13]. Some genes were reported to contain TATA-like motifs, but these were not functionally tested and could be spurious matches [14,15].

Here we address the structure of core promoters in *Phytophthora infestans* and relatives, by using whole-genome analyses to characterize known and novel motifs and assess their association with gene expression patterns. We report that INR and FPR motifs usually occur separately, but come together as an INR plus FPR “supramotif” in 10-15% of promoters where they are linked to higher than average mRNA levels, an increased propensity towards developmental regulation, and gene functions related to pathogenesis. We also identify and functionally test a new motif, DPEP, which tends to associate with housekeeping genes, but fail to obtain convincing evidence for the presence of a TATA box-like sequence. These data help illuminate how the oomycete transcriptional apparatus has evolved, and may be useful for predicting genes within their genomes and optimizing transgene expression.

## Results and discussion

### Transcription start sites in *P. infestans*

To identify the appropriate search space for core promoter motifs, we defined the approximate locations of transcription start sites (TSSs) for *P. infestans* genes. This involved mapping expressed sequence tags (ESTs) against all 17,797 gene models [16]. Of 74,135 available ESTs, 3,129 had 5^′^ termini that mapped upstream of the predicted start codon. A “High Confidence” promoter set was developed based on the 121 genes for which two or more ESTs had their 5^′^ ends upstream of the start codon and within two bases of each other; the upstream EST terminus was inferred to represent the TSS. The distance between these inferred TSSs and the start codon ranged from 32 to 144 nt, with a median of 50 nt. This is just slightly larger than a prior estimate [17]. An “Expanded” promoter set was also developed from 573 genes for which TSSs were predicted with lesser confidence. These represented cases where either only one EST was identified that terminated upstream of the start codon, or where several ESTs were detected but ended more than two bases from each other; the latter might be due to multiple start sites or premature termination of reverse transcription.

As described below, both the High Confidence and Expanded promoter sets were used to identify and preliminarily characterize core motifs. Analyses were later expanded to total promoters, most of which lack defined TSSs and may contain more erroneous gene models due in part to less EST support. A prior study found that as many as 15% of *P. infestans* gene models had incorrect 5^′^ termini, and thus improperly delineated promoters [18]. Total promoters may thus not represent the most sensitive set for motif searching. On the other hand, the ESTs used to define TSSs in the High Confidence and Expanded sets may be biased, even though they were derived from 20 different conditions of growth [19].

### Use of TSS datasets in *de novo* search for core promoter elements

Motifs were predicted by searching for over-represented sequences within 100 nt windows centered on the TSSs. This involved using the motif discovery tool MEME, which employs an expectation maximization technique [20], on the High Confidence and Expanded sets. A 100 nt search space was selected since core promoters typically extend up to 50 nt on either side of a TSS, and the 100 nt region would include the median 5^′^ untranslated region from *P. infestans.* Several rounds of searches were performed using MEME with parameters for motif width that ranged from 5 to 18 nt in different iterations.

MEME identified several candidate motifs including separate INR and FPR-like motifs (7 nt each), a 16 nt motif in which INR and FPR were separated by 2 nt, a new motif referred to later as DPEP (7 nt), and a motif similar to the eukaryotic CCAAT box [21]. The CCAAT box is not considered to be a core element, but does influence initiation and core motif recognition. Also detected was a motif matching the Kozak sequence around the start codon for translation, which was not analyzed further. Sequences corresponding to INR, FPR, and DPEP were more common in the forward orientation, which is consistent with their roles as core motifs.

**Figure 1:**
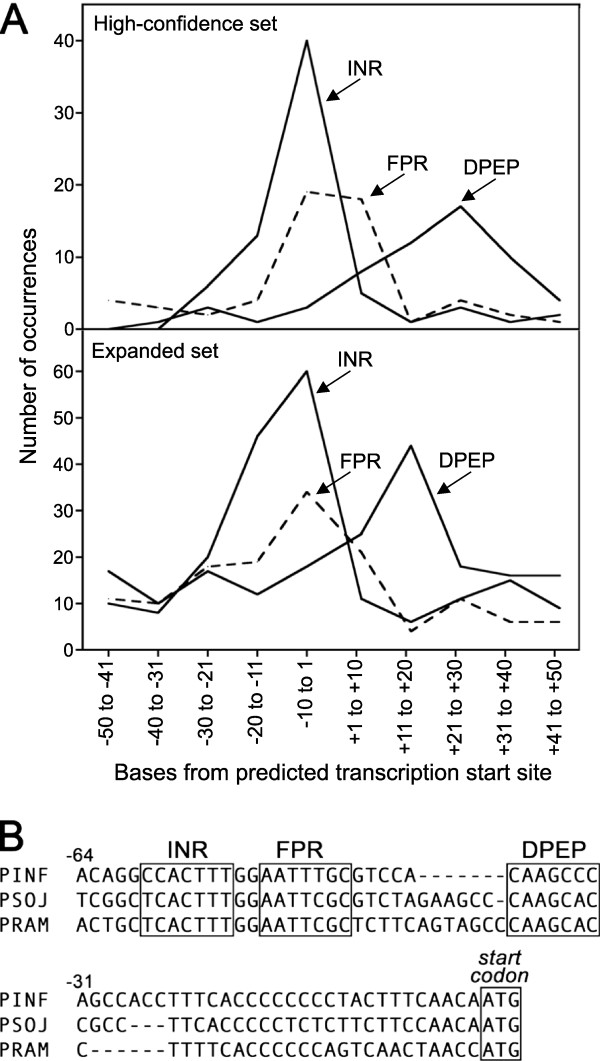
**Location of INR, FPR, and DPEP in *P. infestans *promoters.** (**A**) Positions of motifs relative to TSSs in the High Confidence set (top panel, 121 genes) and the Expanded set (bottom panel, 573 genes). Searches employed motif definitions of YCAYTYY for INR, MWTTTNC for FPR, and SAASMMS for DPEP. (**B**) Alignment of promoter from *P. infestans* gene PITG_10198 and its orthologs from *P. sojae* and *P. ramorum*. Shown in boxes are the three motifs, which are conserved within the orthologous promoters of the three species, and the ATG start codon.

Examination of the INR-like motifs returned by MEME suggested that the definition of the oomycete INR proposed previously, YCATTYY, was too narrow [12]. Our results instead suggested that INR was better-defined by YCAYTYY. This adjustment increased the number of hits near the TSSs in the 121-promoter High Confidence set from 35 to 53, with little increase in background (*p*-value for over-representation in that set of 10^-9^; *p*-value of 10^-23^ in the Expanded set). Systematic tests of variants of this pattern also suggested that YCAYTYY was optimal. As shown in Figure 1, INR mapped just slightly upstream of the TSS in both the High Confidence and Expanded promoter sets. This is consistent with data from mammals where transcription was shown to initiate usually at the adenine [2]. Our definition of INR is narrower than that of the mammalian consensus of YYA_+1_NWYY.

The output from MEME that resembled FPR suggested that the motif was best-defined by MWTTTNC. This is similar to the CAWTTTNYY proposed by McLeod et al. [12], but with more degeneracy and a reduction from 9 to 7 nt. As will be noted later, the shorter definition is also consistent with our results from total *P. infestans* promoters. As shown in Figure 1, FPR typically resided just downstream of the TSS in both the High Confidence and Expanded datasets. It was detected 27 times within the 100 nt window of the 121-promoter High Confidence set (*p*-value for over-representation of 10^-3^; *p*-value of 10^-13^ in the larger Expanded set).

Prior studies of *Phytophthora* promoters considered INR and FPR as adjoining components of an approximately 16 nt conserved block found in most genes, with INR located 5^′^ of FPR [12]. This is inconsistent with our findings, however. Only 16 of the 121 High Confidence promoters contained adjacent INR and FPR motifs, *i.e.* within a 16 nt block, in the 100 nt search window. Moreover, as shown in Figure 1, more matches were detected in the High Confidence dataset to INR than FPR (53 and 27 respectively); both values are well above the false discovery rate for each motif (about 11). This suggests that either our motif definitions are too stringent, or the motifs may be capable of operating separately. Independence of the motifs is consistent with results from a prior study that showed that some mutations in FPR only partially impaired transcription if INR was present, and *vice versa*[12].

As a consequence of our observation, if matches to INR and FPR are found at 5^′^ and 3^′^ ends of the same 16 nt window, we refer to this as the “INR+FPR” supramotif. If INR and FPR are found at more distant sites, these are referred to as separate INR and FPR motifs, *i.e.* INR alone and FPR alone elements.

Our search also identified a new putative core promoter element, which we name DPEP for Downstream Promoter Element Peronosporales, after the taxonomic order within the Oomycota that contains *Phytophthora*. DPEP is unrelated in sequence to the metazoan DPE. As shown in Figure 1, DPEP resides on average 26 nt downstream of the TSS. Unlike INR and FPR which are AT-rich, DPEP has the pattern SAASMMS which is slightly GC-rich. DPEP was detected downstream of the transcription start site in 37 of the 121 promoters in the High Confidence set (*p*-value for over-representation of 10^-2^; *p*-value of 10^-6^ in Expanded set). In only 24 cases was it present in the same promoter with an INR or FPR.

To illustrate the typical spacing between INR, FPR, and DPEP, a promoter containing all three motifs is shown in Figure 1B. Gene PITG_10198, which encodes adenosylhomocysteinase, contains a combined INR+FPR block spanning the transcription start site, followed after 5 bases by a DPEP. All three motifs are well-conserved in the orthologous promoters from *Phytophthora ramorum* and *Phytophthora sojae*. While the distance between INR and FPR is constant in the two species at 2 nt, the space between DPE and FPR ranges from 5 to 12 nt. Variable spacing of the DPE from the INR+FPR block was also noted in alignments of other genes (not shown). It should be noted that while the relative positions of the INR, FPR, and DPE shown in Figure 1B are typical, 46 of the 121 promoters in the High Confidence set lacked all three of the motifs.

### Refinement of element definitions through genome-wide analyses

The preliminary motif definitions from the High Confidence and Expanded sets were used to search upstream of all 17,797 *P. infestans* genes, and the resulting matches were used to establish position-specific probability matrices (PSPMs; provided in Additional file 1). A search of the total gene set was done out of concern that the preliminary definitions might be based on a biased group of promoters. Each PSPM was developed from matches within 200 nt of the translation start site, and then used to search upstream of each gene’s start codon using the FIMO program [20]. A statistical threshold was used to distinguish regions that contained the combined INR+FPR from those with the INR or FPR alone, as described in a following section.

**Figure 2:**
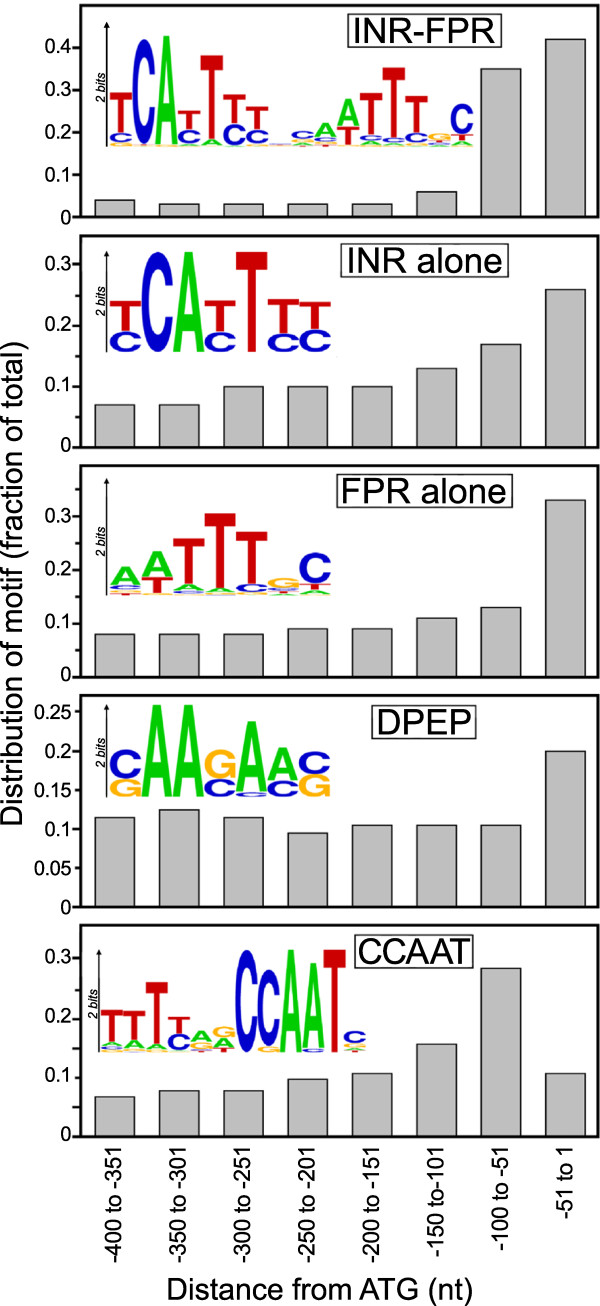
**Positional bias of motifs within total promoters.** Shown are the distributions of sequences matching INR+FPR, INR alone, FPR alone, DPEP, and CCAAT in total *P. infestans* promoters. A sequence logo for each motif, based on hits within 200 nt of the start codon, is also shown in each panel. Matches were recorded based on *p*-value thresholds in the FIMO program of 5×10^-5^ for INR+FPR and CCAAT, and 10^-3^ for the INR, FPR, and DPEP motifs, using weighted matrices reflected by each sequence logo. Values represent hits in the sense orientation for all motifs except CCAAT, for which both orientations are included.

The spatial distributions of INR+FPR, INR alone (*i.e.* without a downstream FPR), FPR alone (*i.e.* without an upstream INR), and DPEP are shown in Figure 2. The figure also presents a sequence logo for each motif. As expected for core promoter motifs, hits were most common at the 3^′^ end of each promoter, within 50-100 nt of the start codon. While the data for these motifs in Figure 2 reflect hits in the forward direction, we also calculated reverse matches to test for orientation bias, which is typical of core promoter elements. The ratios of forward to reverse matches within 100 nt of the start codon were 5.9, 2.9, 1.8, and 1.8 for INR+FPR, INR alone, FPR alone, and DPEP, respectively.

We estimate that 13, 18, 7, and 8% of *P. infestans* promoters harbor INR+FPR, INR alone, FPR alone, and DPEP, respectively, after correcting for false discovery. This is based on a *p*-value cutoff of 5 x 10^-5^ for the 16 nt INR+FPR motif, a threshold which ensures that both its INR and FPR components significantly match the motif in nearly all cases, 10^-3^ cutoffs for the other motifs, and a search space of 200 nt upstream of the translation start site. It should be noted that our estimates of motif occurrence depend on these parameters as well as the quality of the gene models. For example, if a 10^-4^ cutoff and a 500 nt search space was used for the combined INR+FPR motif, that sequence would be found in about 38% of promoters, which we believe might overestimate the number of functional sites.

Regardless of the search parameters employed, it seems that about half of *P. infestans* promoters lack a known core promoter motif. This compares to the situation in humans where about 46% of promoters lack the TATA box and INR, which were the first identified core elements [22]. The subsequent discovery of other elements raised the fraction of human promoters with known core promoter elements to above 95%, although this value may include false positives [4]. In human promoters lacking core elements, upstream enhancers were shown to recruit the pre-initiation complex [23].

It should be noted that “INR alone” is a simplified nomenclature for the motif in *P. infestans,* as is “FPR alone.” The former, for example, refers to cases where there is a strong match to the INR PSPM but a poor or no match of the downstream region to the FPR definition. This category could alternatively be termed “INR-strong, FPR weak/absent”. The issue of variation within INR+FPR sequences is addressed in the next section.

### Heterogeneity of the 16 nt INR+FPR block

**Figure 3:**
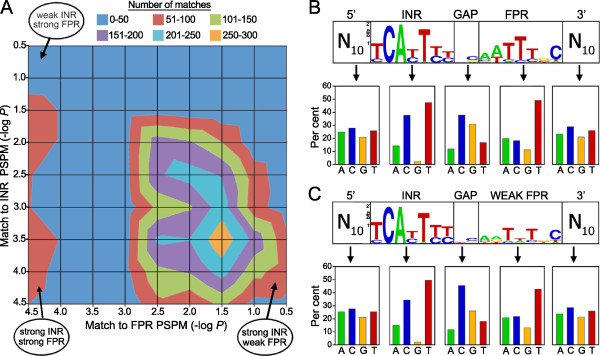
**Heterogeneity within INR+FPR motif.** (**A**) Contour chart showing strength of matches of INR and FPR regions from 5352 *P. infestans* promoters. Sequences within 1 kb of the translation start sites of total *P. infestans* promoters that matched the PSPM for INR+FPR were identified using a *p*-value threshold of 10^-4^ in FIMO. The putative INR and FPR components were then extracted and scored against their respective PSPMs. In the chart, X and Y-axes show the match of the putative INR+FPR region against PSPMs for FPR and INR, respectively, with the Z-axis indicating the number of promoters having each value based on the indicated color code. The lines in the chart connect interpolated points of equal value, similar to that of a two-dimensional topographic map. (**B**) Composition of all 5352 INR+FPR matches from panel A, showing a logo for the motif and base composition of INR and FPR, plus flanking and intervening regions. (**C**) Composition of 241 INR+FPR matches from panel A in which the correspondence to INR was strong (*p*<10^-4^) but FPR was weak (*p*>10^-2^).

Close examination of sequences shown by FIMO to match the PSPM for the combined INR+FPR motif, applying the program’s default *p*-value threshold of 10^-4^, revealed major variation within the motif. This analysis involved splitting each matched region into its 5^′^ and 3^′^ components, which were then scored separately using FIMO against separate PSPMs for INR and FPR, respectively. Of about 5,400 hits in *P. infestans* promoters, only 497 strongly matched (*p*<10^-3^) both the INR and FPR. This is illustrated by the contour chart shown in Figure 3A. As shown in the figure, it was more common to have a robust match against the INR PSPM and a weak match against FPR than the reverse (Figure 3A). An example is gene PITG_18342. It had a sequence matching the most frequent form of INR 64 nt upstream of the start codon (TCATTCT, *p*=7×10^-5^), followed after 2 nt by a weak match to FPR (AAATAGC, *p*=0.05). An inference is that counting occurrences of the INR+FPR motif based on the overall *p*-value or score may overestimate the frequency of the supramotif.

Our conclusion that the 16 nt region is not a well-conserved, monolithic motif may contradict previous interpretations, but is consistent with its likely evolutionary history. While FPR has only been described in oomycetes, INR is widely distributed throughout eukaryotes. Studies in model systems have shown that the main proteins that bind INR are the TAF1 and TAF2 subunits of TFIID, which are widely conserved; the *P. infestans* ortholog of TAF1 is PITG_02547 and there are two TAF2 proteins, PITG_14044 and PITG_18882. Other proteins that bind INR include well-conserved transcriptional regulators such as YY1 (*P. infestans* ortholog, PITG_19177) and taxon-specific proteins such as IBP39 of *Trichomonas* and USF-1 of metazoans [24-26]. We propose that FPR evolved to bind a factor that works with INR-interacting proteins in a subset of *P. infestans* genes. Since INR and FPR are nearly always separated by a 2 nt gap, this spacing is apparently optimal. In other eukaryotes, there are similar examples of synergy between core elements such as TATA and INR, or INR and MTE which must be separated by a certain distance for full effect [27,28].

It is very notable that INR and FPR of *P. infestans* are similar, with positions 3-6 of INR resembling positions 2-5 of FPR. The proteins that bind the two regions could be related and work synergistically. In contrast, in other species only a few genes with tandem INR-like core promoter sequences have been described [29,30].

We also considered the possibility that a sequence other than FPR might occur immediately 3^′^ of INR in some promoters. We therefore submitted to MEME 241 16-nt sequences that matched the INR well but only weakly matched the FPR. This only yielded a consensus that maintained the INR definition but had low information content in the downstream region (Figure 3C). Remarkably, this region maintained the base composition of the canonical FPR. Both are T-rich and G-poor, for example. This composition may aid transcription through mechanisms unrelated to the direct binding of the pre-initiation complex. In *C. elegans,* T-rich blocks within the core promoter were shown to enhance gene expression levels [31], and T-rich regions in *S. cerevisiae* tended to be depleted for nucleosomes and associated with higher rates of transcription [32]. It is notable that the regions upstream and downstream of INR and FPR, respectively, are also slightly pyrimidine-rich (53%) but not to the extreme of INR.It is also interesting that the 2 nt spacer between INR and FPR has the strong bias of 68% G+C. A high G+C content is associated with bendability of DNA [33]. This may facilitate the binding of proteins to both the INR and FPR regions of the core promoter.

### Searches for other core promoter elements

We also used MEME to search the total gene set for additional novel core elements, although none were identified. This included performing discriminative motif discovery by comparing promoters lacking INR+FPR, INR, FPR, and DPEP to a negative set of promoters having those motifs. Several over-represented motifs were identified, but they resided upstream of the TSS (75 to 300 nt) and lacked orientation bias. These are probably enhancers or proximal elements. We also used the High Confidence dataset to search for features near the TSS in promoters lacking an identified core motif. The regions just upstream of such TSSs were enriched for stretches of pyrimidines (43% more than in a randomized dataset, based on triplet counts), but not significantly more than promoters with core motifs.

We also searched the High Confidence, Expanded, and total promoter sets for core promoter elements detected before in metazoans, yeast, and plants. These included several variants of the widely distributed TATA box and motifs specific to plants (Y-patch, Motif 5, Motif 7; [6]) and animals (DPE, MTE, BRE, DRE; [2]). Any hits were not over-represented and not positionally biased, including potential matches to the TATA box, and are therefore unlikely to be authentic.

Despite the apparent absence of the TATA box, *P. infestans* is predicted to encode a TATA-binding protein (TBP) as the product of gene PITG_07312. This 251 amino acid protein shows 66% amino acid identity (83% similarity) to TBP of *S. cerevisiae,* with an *E*-value in BLASTP of 10^-83^*.* An alignment of the *P. infestans* protein with orthologs from *S. cerevisiae, A. thaliana,* human, and five oomycetes is shown in Additional file 2. The N-terminal domain of TBPs are known to be conserved poorly between eukaryotes [34], and this trend is also seen with the oomycete proteins. The remainder of the oomycete proteins are very similar to plant, fungal and human orthologs, with the exception of a novel 26 residue acidic C-terminal tail found only in the oomycete proteins. Within domains of the protein that are well-conserved, the oomycete proteins display several notable differences at sites known to interact with DNA [34]. Of 15 such residues that are totally conserved between *S. cerevisiae, A. thaliana,* and human, nine are different in all five oomycetes. For example, an alanine conserved in the three non-oomycetes (position 191 in *S. cerevisiae*) is changed to threonine in the oomycetes *P. infestans, Phytophthora sojae, Pythium ultimum,* and *Hyaloperonospora arabidopsidis.* Such changes may alter the DNA-binding specificity of the oomycete protein, and may help explain the apparent absence of a TATA box. In contrast, only 2 of 22 residues that contact other components of the preinitiation factor transcription factor complex (TFIIA, TFIIB, and NC2; [34]) are different in oomycetes.

### Relationship of CCAAT box to core promoter elements

We also developed a genome-wide PSPM for the CCAAT box and identified matches in total promoters. The motif had 1953 total hits based on a *p*-value cut-off of 10^-5^, with equal numbers of forward and reverse matches (984 and 969, respectively). Most were within 150 nt of the start codon (Figure 2, bottom panel). When both an INR or INR+FPR and CCAAT element were present, the latter was upstream of the INR 94% of the time at a median distance of 83 nt. This spacing resembles that seen in other eukaryotes [35]. However, while the CCAAT region of the motif is preceded by a GC-rich region in metazoan promoters (GGCCAATCT), that region is T-rich in *P. infestans.* This is consistent with the lack of CpG islands at the 5^′^ ends of *P. infestans* genes, due to an absence of cytosine methylation [36].

### Functional testing of DPEP

Due to the large evolutionary distance between oomycetes and systems in which transcription has been well-studied, it is not surprising that oomycetes contain a novel element such as DPEP. In model systems, new core motifs continued to be identified long after promoters were first examined in detail [22]. Nevertheless, we considered the possibility that DPEP was not an authentic core promoter element but instead a microsatellite since (ACA)_3_, (AAC)_3_, (CAA)_3_, (AGA)_3_, (AAG)_3_, and (GAA)_3_ would include the DPEP pattern. However, only 3% of DPEP hits within promoters fell within such sequences.

**Figure 4:**
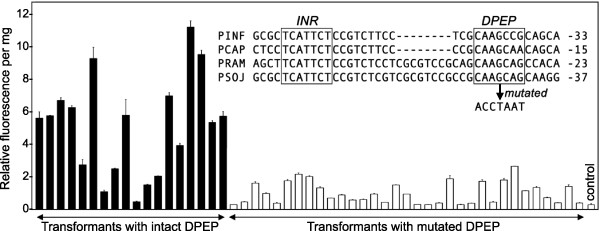
**Mutating DPEP reduces gene expression.** The promoter of PITG_10185 (black bars), or a version with a mutated DPEP (white bars), were fused to the GUS reporter gene and transformed into *P. infestans.* Stable transformants were selected using the *nptII* marker and assayed for GUS activity using a fluorometric assay. Also shown is an alignment of the region of the PITG_10185 promoter that contains DPEP with orthologous promoters from *P. capsici, P. ramorum,* and *P. sojae* (PCAP, PRAM, and PSOJ, respectively)*.* Numbers to the right of the alignment represent distances upstream of the start codon.

We also directly tested DPEP function by measuring the effect of mutating the element on gene expression. This involved tests of the promoter from PITG_10185, which contains a DPEP element 17 nt downstream from the TSS. As shown in Figure 4, this element is also seen in orthologous promoters from *P. capsici, P. ramorum,* and *P. sojae*. Stable transformants were obtained using a plasmid containing a 382 nt promoter fragment from PITG_10185, or a version in which the DPEP was mutated, fused to the GUS reporter gene. Quantitative assays indicated that the mutation reduced expression by an average of 5-fold (Figure 4). The difference was significant at *p*=10^-10^ using Student’s *T*-test, even though expression levels varied within each promoter class due to position effects which are common in *P. infestans*[37]. Mutation of DPEP did not totally eliminate GUS expression, possibly since the PITG_10185 promoter also contains an INR.

We attempted to perform a similar experiment using two promoters that contained DPEP but lacked INR or FPR. However, the intact promoters were too weak to allow the reliable quantification of GUS levels.

### Evolutionary conservation of core motifs from *P. infestans*

**Figure 5:**
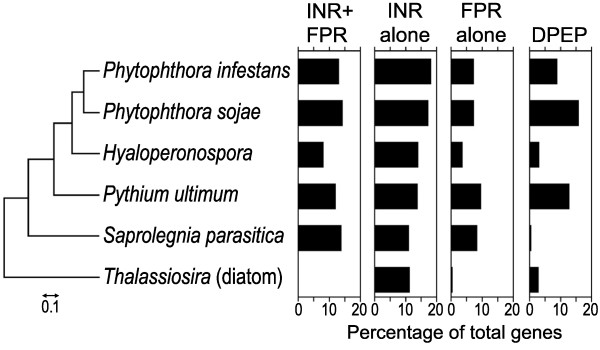
**Distribution of motifs in different species.** Searches for the indicated motifs were performed in five oomycetes (*P. infestans, P. sojae, H. arabidopsidis, Py. ultimum, S. parasitica*) and the diatom *T. pseudonana,* which is a more distant member of the Stramenopile kingdom. Bar graphs show the percent of promoters within each species that contain the motifs within 200 nt upstream of the start codon, corrected for false discovery rates. Values for INR+FPR, INR alone, and FPR alone are corrected to exclude overlap. The figure on the left is a neighbor-joining tree based on ribosomal RNA and internal transcribed spacer (ITS) sequences, which was developed from alignments using the implementation of MUSCLE within the SEAVIEW package [38]. GenBank accession numbers for the sequences are (top to bottom) JF834688.1, HQ643349.1, JF975614.1, JQ898478.1, JX045933.1, and EF208790.1.

Searching for the motifs in other species suggested that the INR+FPR combination was unique to oomycetes (Figure 5). This involved using the PSPMs developed for *P. infestans* to search the first 200 nt of promoters from the oomycetes *P. sojae, H. arabidopsidis, Py. ultimum, and Saprolegnia parasitica*, and several non-oomycetes. *S. parasitica* is a member of the Saprolegniales order, with the rest belonging to the Peronosporales; evolutionary relationships based on ribosomal RNA and internal transcribed spacer sequences are shown in Figure 5. Each oomycete contained promoters with the INR+FPR supramotif, as well as the INR alone or FPR alone motifs. The percentage of promoters with these motifs was similar between *P. infestans* and *P. sojae*, but more varied in the other oomycetes. For example, INR+FPR was less abundant in *H. arabidopsidis* (~8% of genes) than the other Peronosporales (12-14%)*.* This difference was not due to the use of the *P. infestans* PSPM, as similar results were obtained using one based on *H. arabidopsidis*.

Interestingly, INR+FPR PSPMs from each oomycete were nearly identical (Additional file 2). This may explain why two INR+FPR-containing promoters from the downy mildew *Bremia lactucae* have been shown to effectively drive transgene expression across several genera of oomycetes [39-44].

Oomycetes belong to the kingdom Stramenopila along with diatoms, and another stramenopile, the diatom *Thalassiosira pseudonana*, appeared to lack both the INR+FPR and FPR (Figure 5). These motifs also did not appear over-represented in the promoters of *A. thaliana* and *S. cerevisiae* (not shown).

DPEP was not present at significant levels in *S. parasitica*, and thus appeared specific to Peronosporales. DPEP was over-represented in promoters of *T. pseudonana* compared to randomized sequences*,* but these were questionable matches*.* All hits in the diatom involved A and C-containing versions of DPEP, compared to oomycetes where G and C were also common. A large fraction of the hits resided within microsatellites of ACA, CAA, or AAC*.* In addition, while DPEP in oomycetes occurred mostly in the forward orientation, this was not true in *T. pseudonana* after correcting for false discovery*.* While some microsatellites are known to concentrate near TSSs and influence transcription [45], we believe that the DPEP of oomycetes and its matches in *T. pseudonana* lack a common evolutionary history.

### Associations of motifs with expression pattern

Some core promoter elements in model systems have been associated with certain patterns of gene expression. In vertebrates, most housekeeping genes lack a TATA box and instead employ INR sequences near the TSS, while the TATA box is preferentially linked to tissue-specific expression [46]. Housekeeping functions are also associated with genes containing the T-block motif in *C. elegans*[31].

**Figure 6:**
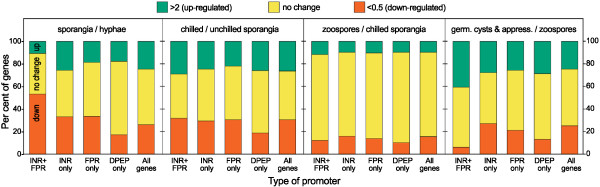
**Association of core promoter motifs with tissue-specific expression.** Shown for each class of genes are the percent that are up- or down-regulated by 2-fold or more at developmental transitions between hyphae and sporangia, sporangia and sporangia chilled to induce zoosporogenesis, chilled sporangia and swimming zoospores, and zoospores and germinated cysts forming appressoria. The values in the panel labeled “sporangia/hyphae”, for example, are based on the mRNA level in sporangia divided by hyphae. The gene classes contained in their promoters the INR+FPR supramotif without DPEP, only INR (*i.e.* INR with a weak or no FPR, and no DPEP), only FPR (*i.e.* an FPR with a weak or no INR, and no DPEP), or only DPEP.

To assess whether relationships existed between expression pattern and the *P. infestans* core motifs, we used data from a microarray study that measured mRNA at five sequential life stages [47]. These were nonsporulating (young) hyphae, sporangia, sporangia undergoing zoosporogenesis (each sporangium reorganizes into 6-10 biflagellated zoospores, triggered by chilling), swimming zoospores, and encysted zoospores forming germ tubes and the plant infection structures called appressoria. Genes were classified into groups that contained only the INR+FPR, INR, FPR, or DPEP. To reduce noise due to false positives, matches were counted only if present in the 125 nt upstream of the start codon for the first three motifs, and 100 nt for DPEP. These search spaces were selected based on studying the data used to generate Figure 2, as motif hit frequencies at more upstream distances were equal to the background. Associations between expression pattern and motif content are shown in Figure 6, which illustrates the fraction of *P. infestans* genes that are up- or down-regulated by 2-fold or more at each developmental transition.

Genes containing the combined INR+FPR in their promoters were much more likely to be differentially expressed. In contrast, genes containing only DPEP or FPR showed the smallest variation. The average percent variance in mRNA levels between each of the five life-stages was 53% higher than average for genes with INR+FPR, 12% higher for INR only-containing genes, and 8% lower for genes in the FPR and DPEP groups.

The most striking difference was observed during the transition from hyphae to sporangia (Figure 6). During this shift, 54% of INR+FPR genes were down-regulated, compared to only 33% of genes in the INR or FPR classes and 26% for all genes. Differences between the INR+FPR group and each of the other classes are statistically significant with *p*<10^-4^. This supports the premise that there is a functional distinction between the canonical INR+FPR supramotif and INR alone, *i.e.* INR with a weak or absent downstream FPR. Expression patterns of INR+FPR genes during the transition from zoospores to germinated cysts was also distinct from the other classes, as 40% were up-regulated compared to about 25% for the other groups. This difference between the INR+FPR class and each of the others is significant with *p*<10^-3^.

It is important to note that each pattern of transcription can be found for some genes within each core promoter class. This is because other transcription factor proteins, not those that bind core elements, likely play the dominant role in determining developmental regulation. For example, of the four genes comprising the *CesA* family of cellulose synthases, which are induced in the appressorium stage [48], only one contains a strong match to the INR+FPR supramotif (*CesA3*, PITG_17007). Moreover, even though relatively few INR+FPR promoters tend to be induced in sporangia, we have shown that inserting binding sites for sporulation-specific transcription factors upstream of a INR+FPR minimal promoter confers sporulation-specific expression [18].

**Figure 7:**
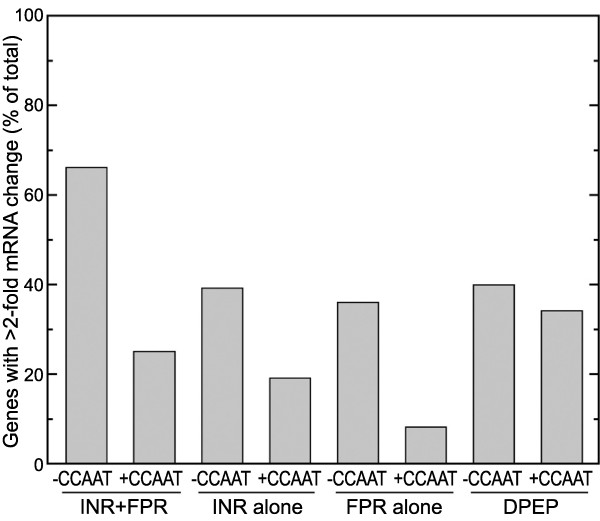
**CCAAT motif reduces tendency towards variable expression.** For each promoter class in Figure 6, genes were categorized based on the presence or absence of CCAAT within 250 nt of the translation start site. Shown are the percentage of total *P. infestans* genes that exhibit >2-fold increases or decreases in mRNA levels between hyphae and sporangia based on microarray analysis.

The presence of a CCAAT box decreased the tendency of genes to vary, regardless of which core element was present (Figure 7). The presence of CCAAT reduced the fraction of genes changing by 2-fold or more between life stages by 62% in the INR+FPR class, 51% in the INR-alone class, and 77% in the FPR-alone class; all of these differences are significant at *p*<10^-2^. This trend was even seen within the DPEP class, which overall showed little variation in mRNA levels during development. CCAAT boxes in model systems have historically also been associated with housekeeping genes, although tissue-specific promoters containing the motif are also known [49,50].

### Association of core promoter motifs with expression level

**Figure 8:**
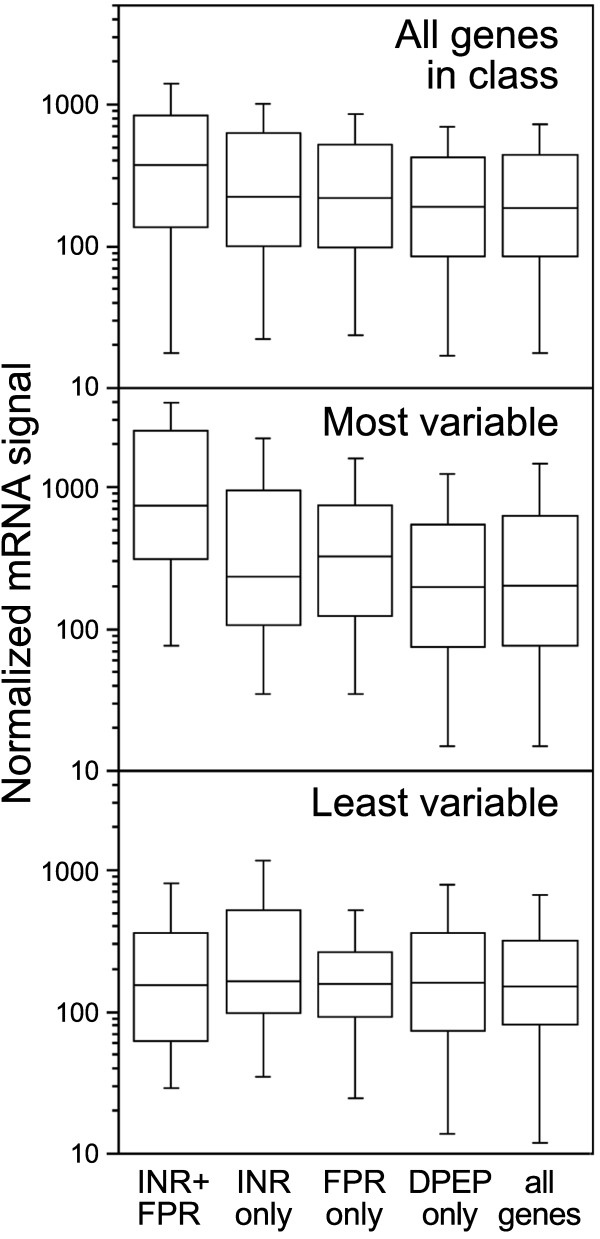
**Association of core promoter motifs with absolute mRNA levels.** Shown for each promoter class are box plots representing mRNA levels for *P. infestans* genes, which are based on the maximum normalized signal observed in microarray analysis of the five life-stages described in Figure 6. Outer whiskers represent datapoints within 1.5× interquartile distances of the 25% and 75% quartiles. The top panel shows all genes, the middle panel shows genes with high variation over the same five life-stages (variance >1.75 in per-gene normalized data), and the lower panel shows genes that vary little between life-stages (variance <0.2).

Correlations between absolute mRNA concentrations and motifs were also observed. This is shown in Figure 8, which illustrates the distribution of maximum expression levels over the five developmental stages. In particular, the median mRNA level of genes with INR+FPR promoters was 1.9 times higher than average (Figure 8, top panel). The distribution of mRNA levels for the INR+FPR genes was significantly different from the other genes (*p*<10^-3^).

One explanation for this finding is that the INR+FPR motif strongly enhances the efficiency of transcription initiation, even more so than INR alone. This is consistent with the observation of Juven-Gershon et al. [51] that combining core motifs in a metazoan promoter raised expression levels. Similarly, in *C. elegans* transcription increased with the number of T-block core promoter elements [31]. There is not a simple relationship between the presence of a core promoter element and mRNA level, however: factors binding the proximal promoter also play a role and the same core element can bind stimulatory or inhibitory factors [52].

The higher transcript levels of INR+FPR genes are partly related to the fact that mRNAs of developmentally regulated genes are more abundant than average. This was revealed by analyzing genes having high and low variation. These were identified by scoring the variance in per-gene normalized data, which discriminated better than Shannon entropy since data were available for only five tissue types [53]. In more constitutively expressed genes, *i.e.* having low variance with *s*^*2*^<0.2, median mRNA levels in each motif class were nearly identical (Figure 8, lower panel). In contrast, the more variable genes (*s*^*2*^>1.75) showed higher mRNA levels than average. This was especially true for the INR+FPR class; its mRNA levels were 3.7-fold higher than all promoter types combined, and were significantly different from the other classes with *p*<0.002.

The possibility was considered that the INR+FPR genes showed higher mRNA levels because more of those genes were expressed at peak levels in hyphae, where transcripts might have more time to accumulate. In contrast, most spore-related stages persist only for a few hours. This did not appear to be the case, however. Transcripts of INR+FPR genes were more abundant regardless of whether they were mostly up-regulated in hyphae (3.4-fold higher mRNA levels than average), sporangia (2.0-fold higher than average), chilled sporangia (2.2-fold), zoospores (2.5-fold), or germinated cysts (2.7-fold). INR+FPR therefore appears to be a special motif that not only is more associated with stage-specific expression, but also with higher absolute levels of transcripts.

### Assessment of promoter class functions using gene ontology terms

All motifs except INR were associated with over-represented Gene Ontology (GO) terms. As shown in Table 1 and Additional file 3, genes containing the INR+FPR supramotif in their promoters were enriched for several biological function and molecular process terms, most of which relate to pathogenesis. The most striking was GO:0044403, which represents symbiosis. INR+FPR genes in this class included RXLR proteins, which oomycetes employ to suppress host plant defenses [54], elicitins, which trigger necrosis in some plants, and elicitin-like proteins [55]. Also enriched was GO:0004650 which stands for polygalacturonase, an enzyme that degrades plant cell walls during infection, as well as GO:0005975 which includes other cell-wall degrading enzymes. These results are consistent with the fact that about half of INR+FPR genes are down-regulated in spores and up-regulated in germinating cysts with appressoria, which is the host penetration stage.

**Table 1:** GO terms over-represented in promoter classes

	**Biological Process**			**Molecular Function**		
Class	Term	Description	*P*	Term	Description	*P*
INR+FPR	GO:0044403	symbiosis	2e-32	GO:0004553	O-glycosyl hydrolase	8e-10
	GO:0005975	carbohydrate metabolism	3e-10	GO:0004650	polygalacturonase	1e-7
	GO:0007047	cell wall organization	8e-7	GO:0016491	oxidoreductase	5e-6
	GO:0055085	transmembrane transport	1e-5	GO:0048037	cofactor binding	7e-5
	GO:0006200	ATP catabolic process	6e-5	GO:0022857	transmembrane transport	7e-5
	GO:0046034	ATP metabolic process	7e-4			
INR alone	none			none		
FPR alone	GO:0044237	cellular metabolic process	1e-3	GO:0005515	protein binding	1e-3
	GO:0050790	regulation of catalytic activity	5e-3			
	GO:0044260	macromolecule metabolism	5e-3			
	GO:0044267	protein metabolic process	7e-3			
DPEP	GO:0044237	cellular metabolic process	1e-3	GO:0003735	structural constituent of ribosome	3e-4
	GO:0009165	nucleotide biosynthesis	1e-3			
CCAAT	GO:0006412	translation	8e-13	GO:0003735	structural constituent of ribosome	2e-18
	GO:0044249	cellular biosynthetic process	3e-12			
	GO:0010467	gene expression	2e-8			
	GO:0009058	biosynthetic process	6e-8			
	GO:0044237	cellular metabolic process	5e-6			
	GO:0006729	tetrahydrobiopterin biosynthesis	2e-3			

Genes containing the indicated motifs that are associated with the over-represented terms are listed in Additional File 3.

In contrast, over-represented terms for DPEP, FPR, and CCAAT were related to housekeeping functions. Examples include transcription (GO:0010467), translation (GO:0006412, GO:003735), and metabolism (GO:0044237, GO:50790, and others). This is consistent with the more constitutive patterns of expression displayed by promoters containing these motifs.

## Conclusions

Our results have led us to re-evaluate the prior belief that most oomycete promoters contain the 16 nt INR+FPR motif. Based on our genome-wide analyses, only a minority (8-15%) of genes in *Phytophthora, Pythium, Hyaloperonospora,* and *Saprolegnia* contain the supramotif. It is easy to understand why the earlier workers came to their conclusion about its prevalence: they had access to only 35 promoters, from a biased gene set that included multigene families [12]. More than half of their genes were inferred to have roles in pathogenesis and others were cloned expressly since they were transcribed at high levels. We have shown that INR+FPR genes are enriched for both traits. We acknowledge that if our searches had employed more liberal thresholds for matches, the number of INR+FPR hits would increase, but only up to about 30-40% of promoters in the oomycetes that were examined.

Regardless of the absolute number of genes containing INR+FPR, the supramotif is interesting since it has a potent effect on transcript levels. Both experimental and bioinformatic studies in other taxa have also shown that combining motifs that otherwise function independently can enhance transcription. For example, in *Drosophila* the TATA and MTE elements act synergistically within INR-containing promoters [27], and the same has been inferred for motif combinations in humans [56]. Combining motifs provides a simple way for organisms to tune expression levels.

The prevalence of INR+FPR in regulated genes, especially those depressed in sporangia relative to hyphae and related to pathogenesis, implies that the supramotif has evolved to interact with a specific subgroup of preinitiation complex-related proteins. In metazoans, the influence of core promoters on developmental regulation is believed to occur at least in part through their interaction with cell-type-specific TBP-associated factors (TAFs) and TBP-related factors (TRFs) [3]. *P. infestans* encodes multiple isoforms of some of these proteins, such as for INR-binding protein TAF2 (PITG_14044, PITG_18888), which could have distinct effects through the life cycle. It is also possible that a *P. infestans* transcription factor that does not participate in preinitiation complex formation has evolved to bind the INR+PFR element. This would resemble the case of transcription factor YY1 of metazoans, which is able to bind the Initiator element and interacts with various protein partners to regulate processes such as embryonic development and cell cycle progression [57].

Even including the new DPEP motif, about half of *P. infestans* genes lack a recognized core motif. It is possible that the pyrimidine-rich stretches common in many promoters may be sufficient to initiate transcription by inducing a particular DNA conformation, or favoring nucleosome eviction [31,32]. *P. infestans* promoters are compact with median intergenic regions of 430 nt, and most known transcription factor binding sites are within 75 to 150 nt of the TSS [58-60]. We hypothesize that in many promoters lacking obvious core elements, DNA is accessible to general transcription factors and nearby regulatory proteins that stimulate initiation. TBP may participate in this process, but not through binding a TATA-box which appears to be absent in oomycetes. TBP may have changed to bind a different sequence in oomycetes. It seems less likely that TBP solely plays a structural function in the oomycete TFIID complex since yeast and human TBP dynamically associate with TFIID, and TFIID integrity does not seem to rely on TBP binding [61].

## Methods

### Promoter extraction and TSS mapping

Genome sequences and GFF files were obtained from the Broad Institute of MIT and Harvard (http://www.broadinstitute.org) for *P. infestans* (v. 2) and *S. parasitica* (v.1)*,* Joint Genome Institute of the United States Department of Energy for *P. sojae* and *T. pseudonana* (each v.3, http://genome.jgi-psf.org)*,* Michigan State University (http://pythium.plantbiology.msu.edu) for *Py. ultimum,* and the Virginia Bioinformatics Institute for *H. arabidopsidis* (v.8.3). These were used to obtain promoter sequences and start codons for each species. To predict TSSs, ESTs from *P. infestans*[19] were aligned to the genome and mapped relative to start codons. A High Confidence set was assembled from 121 promoters where at least two ESTs terminated within 2 nt of each other, and an Expanded set from 573 promoters where a single EST suggested the TSS.

### Detection of motifs and classification of promoters

MEME (v. 4.3 and v. 4.8 [20]) was used to detect over-represented motifs in iterative rounds of searches performed using a range of motif sizes as noted in Results. Other parameters were 11 and 1 for default gap opening (wg) and extension costs (ws), respectively, “anr” for distribution of motifs model (mod), and five iterations. Minimum site values (minsites) of 5 to 10 were used for searches involving the High Confidence and Expanded sets, and 15 to 150 for total promoters which were searched in batches of 2000. In some searches discriminative or negative datasets were used to help find additional motifs, as described in Results.

Motifs from MEME or other sources were identified within promoter sets using PERL scripts developed in-house or FIMO using PSPMs for each motif [20]. *P*-values associated with the strength of motif matches were taken directly from FIMO. For estimating motif frequencies, we searched upstream from start codons, and estimated false discovery rates using same datasets randomized by three rounds of DNA shuffling using shuffleseq in EMBOSS or Shuffle DNA (http://www.bioinformatics.org). Fisher’s Exact Test was used to calculate *p*-values for over-representation of a motif within the searched space. For initial analyses of *P. infestans,* INR+FPR motifs (selected using a p<10^-4^ cut-off) were distinguished from INR and FPR-alone motifs by splitting the putative INR+FPR in half and searching each against INR and FPR PSPMs, respectively, using FIMO; matches of the INR or FPR components with *p*>0.05 were assumed to be weak or insignificant.

Searches for motifs across oomycete species (for Figure 5) were performed using FIMO with the PSPMs developed for *P. infestans.* After correcting for false discovery based on searches of shuffled datasets, similar results were obtained whether 200, 300, or 400 nt search windows had been used; the data in Results are based on using a 200 nt window. For determining whether a hit against the INR+FPR PSPM contained significant matches to both the INR and FPR components, it was found that results similar to the method described in the preceding paragraph (94% concordance) could be obtained by raising the *p*-value cut-off for the FIMO search to 5 x 10^-5^; matches with lower *p*-values were then analyzed separately for INR and FPR. Since the same location might match the INR and FPR definitions, addresses that recorded hits against both were reanalyzed to identify which motif was the closest match, and estimates of INR and FPR frequency were adjusted accordingly. On average, 23% of INR and FPR hits overlapped and required this correction. The observed DPEP frequencies were also adjusted by discarding potential microsatellite hits that were identified using the Microsatellite Repeats Finder program (http://insilico.ehu.es).

### Analysis of microarray data

Expression data were from a prior study that used Affymetrix microarrays to measure transcript levels during growth and development [47]. Robust expression calls were detected for 12,463 of the 15,650 sequences targeted by the arrays, which were designed based on data from partial genome sequencing and expressed sequence tags from isolate 88069. Since the microarrays predated the current draft genome which is based on strain T30-4, we used BLASTN to link the microarray sequences to the predicted T30-4 genes. By selecting the best hit with >97% identity, 7,862 T30-4 genes were matched to the array data. Expression calls from Affymetrix MAS 5.0 software were normalized before analysis, resulting in values that showed good concordance with data from SybrGreen reverse transcription-qPCR studies ([62]; Additional file 2). Analyses of changes during development and variance calculations used per-gene normalized data, set to a mean of 1.0. The significance of the association of a motif with a particular expression pattern was calculated using Fisher’s Exact Test.

### Gene ontology analysis

Annotations were obtained from the Broad Institute and supplemented with published data on effectors [63]. Genes in the INR+FPR, INR alone, FPR alone, and DPEP alone classes were identified as described in Results (*i.e.* based on hits in the 125 nt upstream of the start codon for the first three motifs, and 100 nt for DPEP) and then checked for over-represented GO terms using GOSTAT [64].

### Plasmid construction and analysis of *P. infestans* transformants

A 398 nt portion of DNA upstream of the PITG_10185 open reading frame was amplified by polymerase chain reaction using primers 10185F (5^′^-ATGGATCCGCGT CATGCTTGATCTG) and 10185R (5^′^-ATGAATTCGGTTGAAATTAGAAAAGG), which contain *Bam*HI and *Eco*RI sites at their 5^′^ end, respectively. The amplicon was digested with those enzymes and inserted upstream of the GUS gene in similarly-digested pNPGUS, which is a derivative of pOGUS [65]. A promoter mutated for DPEP was generated using the 10185F with primer 10185PRE (5^′^-TAGAATTCGGTTGAAATTA GAAAAGGAAGG AAGAGATTCTTGCTGATTAGGT).

Transformation of the resulting plasmids was achieved by electroporating zoospores with 30 μg of DNA in a 4-mm cuvette at 550 V, followed by selection on 7 μg/ml G418 [66]. Preliminary assays for GUS were performed by placing a tuft of mycelia at 37°C in 50 μl of 0.1% bromochloroindoyl-β-glucuronide, 50 mM NaPO_4_ pH 7.0, 5 mM K_4_Fe(CN)_6_, 5 mM K_3_Fe(CN)_6_, 0.1% Triton X-100. For quantitative assays, mycelia were grown on clarified rye sucrose media, collected after 5 days, dried on absorbent paper, and placed in a 2-ml tube with two 3.2-mm chrome steel beads and one 6.35-mm bead (Biospec Products, Bartlesville, OK, USA). Samples were frozen in liquid nitrogen, vortexed twice for 30-sec, mixed with 300 μl of extraction buffer (50 mM sodium phosphate pH 7.0, 10 mM EDTA, 10 mM β-mercaptoethanol, 0.1% sodium n-lauroylsarcosine, 0.1% Triton X-100), and clarified by centrifugation for 10 min. Supernatants were assayed for protein using the Bradford assay, and then 20 μg of protein were mixed with 100 μl of 2 mM 4-methylumbelliferyl glucuronide in extraction buffer. After 1 hour at 37°C, 25 μl was mixed with 250 μl of 200 mM sodium carbonate in a black 96-well plate, and fluorescence was measured in a Wallac Victor II plate reader (PerkinElmer Life Sciences, Boston, MA) set to 365 nm excitation and 455 nm emission.

## Competing interests

The authors declare that they have no competing interests.

## Authors’ contributions

SR performed bioinformatics analyses, LP performed functional tests of promoters, HJ planned the experiments and conducted additional bioinformatics analyses, and TJ helped in experimental design. SR and HJ wrote the manuscript. All authors read and approved the final manuscript.

## Supplementary Material

Additional file 1 **PSPMs for the *P. infestans *motifs.** Click here for file

Additional file 2 Comparison of oomycete and non-oomycete TATA-binding proteins, sequence logos of INR+FPR motif from different oomycetes, and correlation analysis of microarray and qRT-PCR data. Click here for file

Additional file 3 List of genes containing motifs associated with over-represented GO terms. Click here for file

## References

[B1] Ohler U Wassarman DA Promoting developmental transcription Development 2010 137 15 26 10.1242/dev.035493 20023156 PMC2796937

[B2] Smale ST Kadonaga JT The RNA polymerase II core promoter Ann Rev Biochem 2003 72 449 479 10.1146/annurev.biochem.72.121801.161520 12651739

[B3] Goodrich JA Tjian R Unexpected roles for core promoter recognition factors in cell-type-specific transcription and gene regulation Nature Rev Genet 2010 11 549 558 10.1038/ni0710-549 20628347 PMC2965628

[B4] Jin VX Singer GA Agosto-Perez FJ Liyanarachchi S Davuluri RV Genome-wide analysis of core promoter elements from conserved human and mouse orthologous pairs BMC Bioinformatics 2006 7 114 10.1186/1471-2105-7-114 16522199 PMC1475891

[B5] Whittington JE Delgadillo RF Attebury TJ Parkhurst LK Daugherty MA Parkhurst LJ TATA-binding protein recognition and bending of a consensus promoter are protein species dependent Biochemistry 2008 47 7264 7273 10.1021/bi800139w 18553934

[B6] Civan P Svec M Genome-wide analysis of rice (*Oryza sativa* L. subsp. *japonica*) TATA box and Y Patch promoter elements Genome 2009 52 294 297 10.1139/G09-001 19234558

[B7] Molina C Grotewold E Genome wide analysis of *Arabidopsis* core promoters BMC Genomics 2005 6 25 10.1186/1471-2164-6-25 15733318 PMC554773

[B8] Brick K Watanabe J Pizzi E Core promoters are predicted by their distinct physicochemical properties in the genome of *Plasmodium falciparum* Genome Biol 2008 9 R178 10.1186/gb-2008-9-12-r178 19094208 PMC2646282

[B9] Abeel T Saeys Y Bonnet E Rouze P Van de Peer Y Generic eukaryotic core promoter prediction using structural features of DNA Genome Res 2008 18 310 323 10.1101/gr.6991408 18096745 PMC2203629

[B10] Roger AJ Simpson AG Evolution: revisiting the root of the eukaryote tree Current Biol 2009 19 R165 167 10.1016/j.cub.2008.12.032 19243692

[B11] Judelson HS Tyler BM Michelmore RW Regulatory sequences for expressing genes in oomycete fungi Molec Gen Genet 1992 234 138 146 1495476 10.1007/BF00272355

[B12] McLeod A Smart CD Fry WE Core promoter structure in the oomycete *Phytophthora infestans* Eukaryot Cell 2004 3 91 99 10.1128/EC.3.1.91-99.2004 14871940 PMC329498

[B13] Pieterse CMJ Van West P Verbakel HM Brasse PWHM Van Den Berg-Velthuis GCM Govers F Structure and genomic organization of the *ipiB* and *ipiO* gene clusters of *Phytophthora infestans* Gene 1994 138 67 77 10.1016/0378-1119(94)90784-6 8125319

[B14] Yan HZ Liou RF Cloning and analysis of pp pg1, an inducible endopolygalacturonase gene from the oomycete plant pathogen *Phytophthora parasitica* Fungal Genet Biol 2005 42 339 350 10.1016/j.fgb.2005.01.003 15749053

[B15] Judelson HS Michelmore RW Structure and expression of a gene encoding heat-shock protein Hsp70 from the oomycete fungus *Bremia lactucae* Gene 1989 79 207 218 10.1016/0378-1119(89)90203-5 2792764

[B16] Haas BJ Kamoun S Zody MC Jiang RH Handsaker RE Cano LM Grabherr M Kodira CD Raffaele S Torto-Alalibo T Genome sequence and analysis of the Irish potato famine pathogen *Phytophthora infestans* Nature 2009 461 393 398 10.1038/nature08358 19741609

[B17] Win J Kanneganti T-D Torto-Alalibo T Kamoun S Computational and comparative analyses of 150 near full-length cDNA sequences from the oomycete plant pathogen *Phytophthora infestans* Fungal Genet Biol 2006 43 20 33 10.1016/j.fgb.2005.10.003 16380277

[B18] Roy S Kagda M Judelson HS Genome-wide prediction and functional validation of promoter motifs regulating gene expression in spore and infection stages of *Phytophthora infestans* PLoS Pathog 2013 in press 10.1371/journal.ppat.1003182 PMC3597505 23516354

[B19] Randall TA Dwyer RA Huitema E Beyer K Cvitanich C Kelkar H Ah Fong AMV Gates K Roberts S Yatzkan E Large-scale gene discovery in the oomycete *Phytophthora infestans* reveals likely components of phytopathogenicity shared with true fungi Molec Plant-Microbe Inter 2005 18 229 243 10.1094/MPMI-18-0229 15782637

[B20] Bailey TL Bodén M Buske FA Frith M Grant CE Clementi L Ren J Li WW Noble WS MEME SUITE: tools for motif discovery and searching Nucleic Acids Res 2009 37 W202 W208 10.1093/nar/gkp335 19458158 PMC2703892

[B21] Dolfini D Zambelli F Pavesi G Mantovani R A perspective of promoter architecture from the CCAAT box Cell Cycle 2009 8 4127 4137 10.4161/cc.8.24.10240 19946211

[B22] Yang C Bolotin E Jiang T Sladek FM Martinez E Prevalence of the initiator over the TATA box in human and yeast genes and identification of DNA motifs enriched in human TATA-less core promoters Gene 2007 389 52 65 10.1016/j.gene.2006.09.029 17123746 PMC1955227

[B23] George AA Sharma M Singh BN Sahoo NC Rao KV Transcription regulation from a TATA and INR-less promoter: spatial segregation of promoter function EMBO J 2006 25 811 821 10.1038/sj.emboj.7600966 16437157 PMC1383549

[B24] Smith AJ Chudnovsky L Simoes-Barbosa A Delgadillo-Correa MG Jonsson ZO Wohlschlegel JA Johnson PJ Novel core promoter elements and a cognate transcription factor in the divergent unicellular eukaryote *Trichomonas vaginalis* Molec Cell Biol 2011 31 1444 1458 10.1128/MCB.00745-10 21245378 PMC3135286

[B25] Fry CJ Farnham PJ Context-dependent transcriptional regulation J Biol Chem 1999 274 29583 29586 10.1074/jbc.274.42.29583 10514422

[B26] Du H Roy AL Roeder RG Human transcription factor USF stimulates transcription through the initiator elements of the HIV-1 and the Ad-ML promoters EMBO J 1993 12 501 511 8440240 10.1002/j.1460-2075.1993.tb05682.x PMC413233

[B27] Lim CY Santoso B Boulay T Dong E Ohler U Kadonaga JT The MTE, a new core promoter element for transcription by RNA polymerase II Genes Devel 2004 18 1606 1617 10.1101/gad.1193404 15231738 PMC443522

[B28] Emami KH Jain A Smale ST Mechanism of synergy between TATA and initiator: Synergistic binding of TFIID following a putative TFIIA-induced isomerization Genes Devel 1997 11 3007 3019 10.1101/gad.11.22.3007 9367983 PMC316697

[B29] Pelletier MR Hatada EN Scholz G Scheidereit C Efficient transcription of an immunoglobulin kappa promoter requires specific sequence elements overlapping with and downstream of the transcriptional start site Nucleic Acids Res 1997 25 3995 4003 10.1093/nar/25.20.3995 9321649 PMC147016

[B30] Bingle CD Craig RW Swales BM Singleton V Zhou P Whyte MK Exon skipping in Mcl-1 results in a bcl-2 homology domain 3 only gene product that promotes cell death J Biol Chem 2000 275 22136 22146 10.1074/jbc.M909572199 10766760

[B31] Grishkevich V Hashimshony T Yanai I Core promoter T-blocks correlate with gene expression levels in *C. elegans* Genome Res 2011 21 707 717 10.1101/gr.113381.110 21367940 PMC3083087

[B32] Lee W Tillo D Bray N Morse RH Davis RW Hughes TR Nislow C A high-resolution atlas of nucleosome occupancy in yeast Nat Genet 2007 39 1235 1244 10.1038/ng2117 17873876

[B33] Vinogradov AE DNA helix: the importance of being GC-rich Nucleic Acids Res 2003 31 1838 1844 10.1093/nar/gkg296 12654999 PMC152811

[B34] Nikolov DB Chen H Halay ED Hoffman A Roeder RG Burley SK Crystal structure of a human TATA box-binding protein/TATA element complex Proc Natl Acad Sci USA 1996 93 4862 4867 10.1073/pnas.93.10.4862 8643494 PMC39370

[B35] Mantovani R A survey of 178 NF-Y binding CCAAT boxes Nucleic Acids Res 1998 26 1135 1143 10.1093/nar/26.5.1135 9469818 PMC147377

[B36] Judelson HS Tani S Transgene-induced silencing of the zoosporogenesis-specific PiNIFC gene cluster of *Phytophthora infestans* involves chromatin alterations Eukaryot Cell 2007 6 1200 1209 10.1128/EC.00311-06 17483289 PMC1951104

[B37] Judelson HS Dudler R Pieterse CMJ Unkles SE Michelmore RW Expression and antisense inhibition of transgenes in *Phytophthora infestans* is modulated by choice of promoter and position effects Gene 1993 133 63 69 10.1016/0378-1119(93)90225-R 8224895

[B38] Gouy M Guindon S Gascuel O SeaView version 4: a multiplatform graphical user interface for sequence alignment and phylogenetic tree building Molec Biol Evol 2010 27 221 224 10.1093/molbev/msp259 19854763

[B39] Mort-Bontemps M Fevre M Transformation of the oomycete *Saprolegnia monoica* to hygromycin B resistance Current Genet 1997 31 272 275 10.1007/s002940050205 9065391

[B40] Horner NR Grenville-Briggs LJ van West P The oomycete *Pythium oligandrum* expresses putative effectors during mycoparasitism of *Phytophthora infestans* and is amenable to transformation Fungal Biol 2011 116 24 41 22208599 10.1016/j.funbio.2011.09.004

[B41] Weiland JJ Transformation of *Pythium aphanidermatum* to geneticin resistance Current Genet 2003 42 344 352 10.1007/s00294-002-0359-y 12612808

[B42] Judelson HS Tyler BM Michelmore RW Transformation of the oomycete pathogen, *Phytophthora infestans* Molec Plant-Microbe Inter 1991 4 602 607 10.1094/MPMI-4-602 1804404

[B43] Judelson HS Coffey MD Arredondo FR Tyler BM Transformation of the oomycete pathogen *Phytophthora megasperma* f. sp. *glycinea* occurs by DNA integration into single or multiple chromosomes Current Genet 1993 23 211 218 10.1007/BF00351498 8382110

[B44] Huitema E Smoker M Kamoun S A straightforward protocol for electro-transformation of *Phytophthora capsici* zoospores Methods Mol Biol 2011 712 129 135 10.1007/978-1-61737-998-7_11 21359805

[B45] Lu Q Wallrath LL Allan BD Glaser RL Lis JT Elgin SC Promoter sequence containing (CT)n. (GA)n repeats is critical for the formation of the DNase I hypersensitive sites in the Drosophila hsp26 gene J Molec Biol 1992 225 985 998 10.1016/0022-2836(92)90099-6 1377279

[B46] Schug J Schuller WP Kappen C Salbaum JM Bucan M Stoeckert CJ Jr Promoter features related to tissue specificity as measured by Shannon entropy Genome Biol 2005 6 R33 10.1186/gb-2005-6-4-r33 15833120 PMC1088961

[B47] Judelson HS Ah-Fong AM Aux G Avrova AO Bruce C Cakir C da Cunha L Grenville-Briggs L Latijnhouwers M Ligterink W Gene expression profiling during asexual development of the late blight pathogen *Phytophthora infestans* reveals a highly dynamic transcriptome Mol Plant Microbe Interact 2008 21 433 447 10.1094/MPMI-21-4-0433 18321189

[B48] Grenville-Briggs LJ Anderson VL Fugelstad J Avrova AO Bouzenzana J Williams A Wawra S Whisson SC Birch PR Bulone V Cellulose synthase in *Phytophthora infestans* is required for normal appressorium formation and successful infection of potato Plant Cell 2008 20 720 738 10.1105/tpc.107.052043 18349153 PMC2329931

[B49] Dylan W Promoters for housekeeping genes Trends Genet 1986 2 196 197

[B50] Laloum T De Mita S Gamas P Baudin M Niebel A CCAAT-box binding transcription factors in plants: Y so many Trends Plant Sci 2012 http://dx.doi.org/10.1016/j.tplants.2012.07.004. 10.1016/j.tplants.2012.07.004 22939172

[B51] Juven-Gershon T Cheng S Kadonaga JT Rational design of a super core promoter that enhances gene expression Nat Methods 2006 3 917 922 10.1038/nmeth937 17124735

[B52] Albert TK Grote K Boeing S Meisterernst M Basal core promoters control the equilibrium between negative cofactor 2 and preinitiation complexes in human cells Genome Biol 2010 11 R33 10.1186/gb-2010-11-3-r33 20230619 PMC2864573

[B53] Fuhrman S Cunningham MJ Wen X Zweiger G Seilhamer JJ Somogyi R The application of shannon entropy in the identification of putative drug targets Bio Systems 2000 55 5 14 10.1016/S0303-2647(99)00077-5 10745103

[B54] Stassen J Van den Ackerveken G How do oomycete effectors interfere with plant life? Curr Opin Plant Biol 2011 14 407 414 10.1016/j.pbi.2011.05.002 21641854

[B55] Jiang RH Tyler BM Whisson SC Hardham AR Govers F Ancient origin of elicitin gene clusters in *Phytophthora* genomes Molec Biol Evol 2005 23 338 351 10.1093/molbev/msj039 16237208

[B56] Gershenzon NI Ioshikhes IP Synergy of human Pol II core promoter elements revealed by statistical sequence analysis Bioinformatics 2005 21 1295 1300 10.1093/bioinformatics/bti172 15572469

[B57] Kim J Kim J YY1’s longer DNA-binding motifs Genomics 2009 93 152 158 10.1016/j.ygeno.2008.09.013 18950698 PMC2668202

[B58] Ah Fong A Xiang Q Judelson HS Architecture of the sporulation-specific Cdc14 promoter from the oomycete *Phytophthora infestans* Eukaryot Cell 2007 6 2222 2230 10.1128/EC.00328-07 17951514 PMC2168256

[B59] Xiang Q Kim KS Roy S Judelson HS A motif within a complex promoter from the oomycete *Phytophthora infestans* determines transcription during an intermediate stage of sporulation Fungal Genet Biol 2009 46 400 409 10.1016/j.fgb.2009.02.006 19250972

[B60] Tani S Judelson HS Activation of zoosporogenesis-specific genes in *Phytophthora infestans* involves a 7-nucleotide promoter motif and cold-induced membrane rigidity Eukaryot Cell 2006 5 745 752 10.1128/EC.5.4.745-752.2006 16607021 PMC1459674

[B61] Asturias FJ TFIID: a closer look highlights its complexity Structure 2009 17 1423 1424 10.1016/j.str.2009.10.004 19913474 PMC2789007

[B62] Judelson HS Shrivastava J Manson J Decay of genes encoding the oomycete flagellar proteome in the downy mildew *Hyaloperonospora arabidopsidis* PLoS One 2012 7 e47624 10.1371/journal.pone.0047624 23077652 PMC3471859

[B63] Raffaele S Win J Cano LM Kamoun S Analyses of genome architecture and gene expression reveal novel candidate virulence factors in the secretome of *Phytophthora infestans* BMC Genomics 2010 11 637 10.1186/1471-2164-11-637 21080964 PMC3091767

[B64] Beissbarth T Speed TP GOstat: find statistically overrepresented Gene Ontologies within a group of genes Bioinformatics 2004 20 1464 1465 10.1093/bioinformatics/bth088 14962934

[B65] Cvitanich C Judelson HS A gene expressed during sexual and asexual sporulation in *Phytophthora infestans* is a member of the Puf family of translational regulators Eukaryot Cell 2003 2 465 473 10.1128/EC.2.3.465-473.2003 12796291 PMC161445

[B66] Ah-Fong AM Bormann-Chung CA Judelson HS Optimization of transgene-mediated silencing in *Phytophthora infestans* and its association with small-interfering RNAs Fungal Genet Biol 2008 45 1197 1205 10.1016/j.fgb.2008.05.009 18599326

