# Supplementary material for: Novel core promoter elements in the oomycete pathogen Phytophthora infestans and their influence on expression detected by genome-wide analysis
Source: BMC Genomics. 2013 Feb 16;14:106. doi: 10.1186/1471-2164-14-106 (PMC3599244; doi:10.1186/1471-2164-14-106)
Supplement: Additional file 1 — PSPMs for the P. infestans motifs. [file 1471-2164-14-106-S1.pdf]

Position Specific Probability Matrices for *P. infestans* (Alphabet=ACGT)

<u>INR</u>

0.003717	0.163569	0.055762	0.776952
0.026022	0.955390	0.014870	0.003717
0.992565	0.000000	0.007435	0.000000
0.066914	0.375465	0.011152	0.546468
0.081784	0.007435	0.011152	0.899628
0.011825	0.484491	0.011341	0.492343
0.014165	0.398810	0.013040	0.573985

<u>FPR</u>

0.765000	0.150000	0.070000	0.015000
0.635000	0.000000	0.000000	0.365000
0.015000	0.050000	0.035000	0.900000
0.000000	0.005000	0.000000	0.995000
0.000000	0.100000	0.025000	0.875000
0.125000	0.210000	0.600000	0.065000
0.031372	0.823095	0.034622	0.110911

<u>INR+FPR</u>

0.003125	0.209375	0.084375	0.703125
0.015625	0.959375	0.012500	0.012500
0.965625	0.003125	0.031250	0.000000
0.103125	0.325000	0.009375	0.562500
0.106250	0.006250	0.015625	0.871875
0.021875	0.487500	0.000000	0.490625
0.006250	0.412500	0.021875	0.559375
0.134375	0.296875	0.315625	0.253125
0.184375	0.471875	0.259375	0.084375
0.600000	0.212500	0.087500	0.100000
0.606250	0.003125	0.050000	0.340625
0.125000	0.062500	0.006250	0.806250
0.059375	0.012500	0.037500	0.890625
0.028125	0.112500	0.046875	0.812500
0.115625	0.175000	0.534375	0.175000
0.100000	0.690625	0.043750	0.165625

DPE

0.000000	0.623616	0.376384	0.000000
1.000000	0.000000	0.000000	0.000000
1.000000	0.000000	0.000000	0.000000
0.000000	0.420664	0.579336	0.000000
0.774908	0.225092	0.000000	0.000000
0.594096	0.405904	0.000000	0.000000
0.000000	0.538745	0.461255	0.000000

CCAAT

0.036667	0.073333	0.123333	0.766667
0.216667	0.010000	0.096667	0.676667
0.063333	0.006667	0.026667	0.903333
0.013333	0.433333	0.003333	0.550000
0.553333	0.023333	0.406667	0.016667
0.260000	0.000000	0.630000	0.110000
0.000000	0.996667	0.003333	0.000000
0.000000	0.956667	0.043333	0.000000
1.000000	0.000000	0.000000	0.000000
0.940000	0.053333	0.006667	0.000000
0.000000	0.000000	0.000000	1.000000
0.063333	0.683333	0.183333	0.070000
