# Supplementary material for: Novel core promoter elements in the oomycete pathogen Phytophthora infestans and their influence on expression detected by genome-wide analysis
Source: BMC Genomics. 2013 Feb 16;14:106. doi: 10.1186/1471-2164-14-106 (PMC3599244; doi:10.1186/1471-2164-14-106)
Supplement: Additional file 2 — Comparison of oomycete and non-oomycete TATA-binding proteins, sequence logos of INR+FPR motif from different oomycetes, and correlation analysis of microarray and qRT-PCR data. [file 1471-2164-14-106-S2.pdf]

Comparison of eukaryotic TBPs

```
        1
                                                                                  oo
SC      ---------- ---------- ---------- ---------- --MADEERLK EFKEANKIV- --FDSNTRQV
AT      ---------- ---------- ---------- ---------- ---------- ---------- ----------
HS      MDQNNSLPPY AQGLASPQGA MTPGIPIFSP MMPYGTGLTP QPIQNTNSLS ILEEQQRQAT QGTSGQAPQL
PI      ---------- ---------- ---------- ---------M ----KK---- ---------- --ARTTSHTV
PS      ---------- ---------- ---------- -----MGKTP ----VA---- ---------- --TRSSARAV
HP      ---------- ---------- ---------- -----MSKTP ---AAS---- ---------- --TRSSSRTV
PU      ---------- ---------- ---------- -----MAPTP PAAAVH---- ---------- --VATRSRAV
SP      ---------- ---------- ---------- ---------- ---------- ---------- ----------
THAL    ---------- ---------- ---------- ---------- ---------- ---------- ----------
PHAE    ---------- ---------- ---------- ---------- ---------- ---------- ----------

       71
        ●                            oo      o o     ● ●   ● o  o o
SC      WE------NQ NRDGTKPATT FQSEEDIKRA APESEKDTSA TSGIVPTLQN IVATVTLGCR LDLKTVALHA
AT      MT------DQ GLEG------ ---------- -SNPVDLSKH PSGIVPTLQN IVSTVNLDCK LDLKAIALQA
HS      FHSQT-LTTA PLPGTTPLYP ---SPMTPMT PITPATPASE SSGIVPQLQN IVSTVNLGCK LDLKTIALRA
PI      KERQLALVEK P--------- GRDLQTVGAR ELQVDDDWEE RLNLEVKIMN VVGTVDLKTP LDLKTIALHA
PS      KERQLALVEE P--------- GRELQAVGSR ELQLDDDWEE RLNLQVKIMN VVGTVDLKTP LDLKTIALHA
HP      RERQLVLIDD P--------- RQYLKSVDAH ELQVDDDWEE RLNLQVKIMN VVGTVDLKTP LDLKVIALHA
PU      KERQLAIVED P--------- RRDLQALEDR EIQVDDDWED KLNLQVKIMN VVGTVDLKTP LELKTIALHA
SP      --------MA A--------- KESSLVAPER ALDVEEEWSQ E-RLPLHVKN VVGTIDVQVP LDLTTIALNA
THAL    ---------- ---------- ---------- ---------- ---------- ---TADLGVR LDLKQIALRC
PHAE    ---------- ---------- ---------- ---------- ----MIRIQN MSSTVNLGTR LDLKKIALKC

      141
                      o    o  o                              ● ●    o           o o
SC      RNAEYNPKRF AAVIMRIREP KTTALIFASG KMVVTGAKSE DDSKLASRKY ARIIQKIGF- AAKFTDFKIQ
AT      RNAEYNPKRF AAVIMRIREP KTTALIFASG KMVCTGAKSE DFSKMAARKY ARIVQKLGF- PAKFKDFKIQ
HS      RNAEYNPKRF AAVIMRIREP RTTALIFSSG KMVCTGAKSE EQSRLAARKY ARVVQKLGF- PAKFLDFKIQ
PI      RNAEYNPKRF SAVIMRLRDP KTTALIFGSG KIVITGGTSE DSCRLAARKF TRVIQKLNF- PAKFTEFKVR
PS      RNAEYNPKRF SAVIMRLRDP KTTALIFGSG KIVITGGTSE ESCRLAARKF TRVIQKLNF- PAKFTEFKVR
HP      RNAEYNPKRF SAVIMRLRDP KTTALIFGSG KIVITGGTSE ESCRLAARKF TRVIQKLNF- PAKFTEFKVR
PU      RNAEYNPKRF SAVIMRLRDP KTTALIFGSG KIVITGGTSE ESCRLAARKF TR---KLNF- PARFTEFKVR
SP      RNTEYNPRRF AAVIMRLRDP KTTALIFASG KIVITGATTE KNCRIAARKY CKVLQKLNF- PAKFEMFKIC
THAL    RNTEFNPRRF AAVIMRLREP RATALLFSSG RMVITGTKSS HNCSLATKKF AYILERVGFQ PAGFIDFKIQ
PHAE    RNTEFNPRRF GAVIMRLREP RATALIFSSG KMCVTGVKST HNANLAAKKF AYIVERVGFK PKENIDFKVQ

      211
        o   ●
SC      NIVGSCDVKF PIRLEGLAFS HGTFSSYEPE LFPGLIYRMV KPKIVLLIFV SGKIVLTGAK QREEIYQAFE
AT      NIVGSCDVKF PIRLEGLAYS HAAFSSYEPE LFPGLIYRMK VPKIVLLIFV SGKIVITGAK MRDETYKAFE
HS      NMVGSCDVKF PIRLEGLVLT HQQFSSYEPE LFPGLIYRMI KPRIVLLIFV SGKVVLTGAK VRAEIYEAFE
PI      NVMGTCDIRF PIRLEGLLND HARFSSYEPE LFPGLIYKLV EPKLTLLIFV SGKIVLCGAR DSNHLHQAID
PS      NVMGTCDIRF PIRLEGLLND HARFSSYEPE LFPGLIYKLV EPKLTLLIFV SGKIVLCGAR DSNHLHQAID
HP      NVMGTCDVRF PIRLEGLLND HARFSSYEPE LFPGLIYKLV EPKLTLLIFV SGKIVLCGAR DSNHLHQAID
PU      NVMGTCDIRF PIRLEGLLND HARFSSYEPE LFPGLIYKLV EPKLTLLIFV SGKIVLCGAR DSSHLNQAID
SP      NMMGTSDVRF PIRLEGLLND HSRFCTYEPE LFPGLIFKLV EPKLTFLIFV SGKLVICGAK ETSDMRLALE
THAL    NVVGTVDVGF PIRLEGVVYA HPTFSSYEPE LFPGLIYRLV QPRVVLLIFV SGKVVITGAK TEEQMVEALR
PHAE    NIVGTADVGF PIRLEGLVYA HSAFASYEPE LFPGLIYRFV NPRVVFLIFV SGKIVITGAK KEMDLAHALT

      281
SC      AIYPVLSEFR KM-------- ---------- ---------- ----
AT      NIYPVLSEFR KIQQ------ ---------- ---------- ----
HS      NIYPILKGFR KTT------- ---------- ---------- ----
PI      KMYPVLLQYR KMTAPPSLMT GK---PGYED SEDLDRLEPG ANV-
PS      KMYPVLLQYR KMTAPPSLMT GK---PGYED SEDLDRLEPG ANV-
HP      KMYPVLLQYR KMTAPPSLMT GK---RGYED SEDLDRLEAG QANV
PU      KMYPVLLQYR KMTAPPSLVT GK---PGYED TEDMDRLEPS AIV-
SP      KMHPLLLEYR KMAPAPSLES L-----DFEE DADIDQHKI- ----
THAL    KVYPVLVEFR ---------- ---------- ---------- ----
PHAE    KLFPVLMEFK KTHITTVPGV PAASLPPTGK TVDTESV--- ----
```

Alignments of TATA binding protein (TBP) orthologs, created using MUSCLE. Symbols above alignment denote DNA-interacting residues; filled circles are cases where the sequence is well conserved in humans, *Saccharomyces cerevisiae,* and *Arabidopsis thaliana,* but different in all oomycetes. Sequences are from *S. cerevisiae* (SC; GenBank AEP68421.1), *A. thaliana* (AT; NP_187953.1), human (HS; AAI09055.1), *Phytophthora infestans* (PI; EEY53626.1 [PITG_07312]), *Phytophthora sojae* (PS; EGZ26194.1), *Hyaloperonospora arabidopsidis* (HP; correction of gene model 813721), *Pythium ultimum* (PU; correction of model PYU1_G013989), *Saprolegnia parasitica* (SP; Broad Institute SPRG_03180.2), and the diatoms *Thalassiosira pseudonana* (THAE; XP_002293666.1 [lacks N-terminus]), and *Phaeodactylum tricornutum* (PHAE; XP_002186321.1). Numbers indicate location within the *S. cerevisiae* protein.

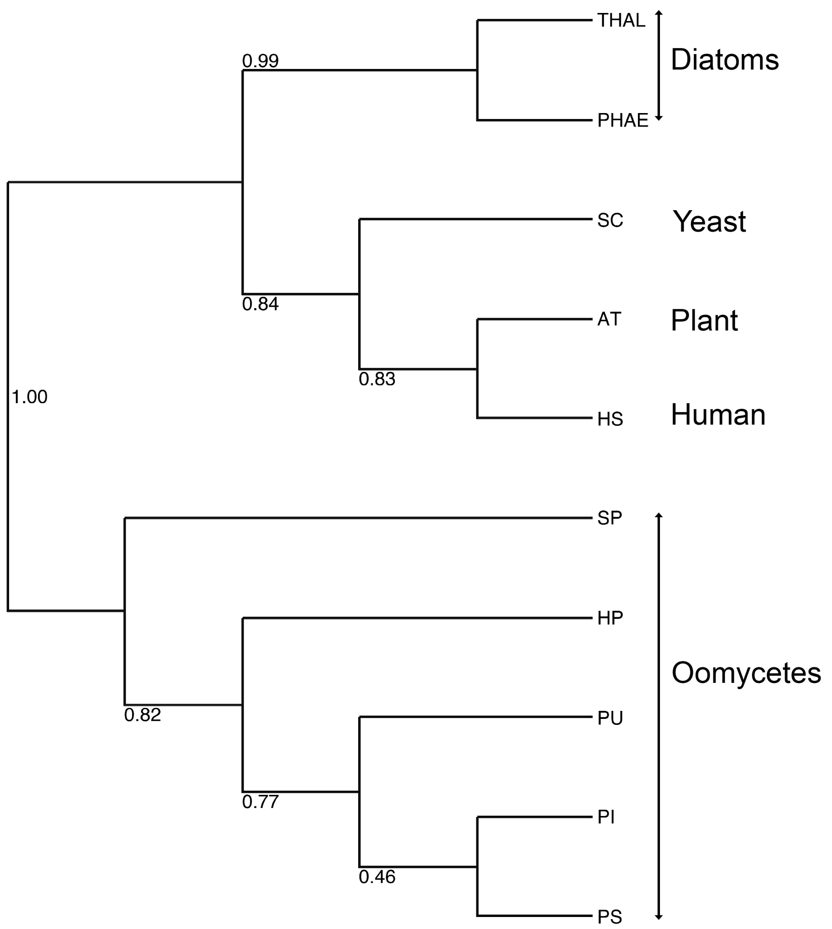

Cladogram showing relationships between TATA-binding proteins shown on the preceding page, made using the PhyML program. Branch support is indicated by aLRT values.

Sequence logos of INR+FPR supramotif from different oomycetes

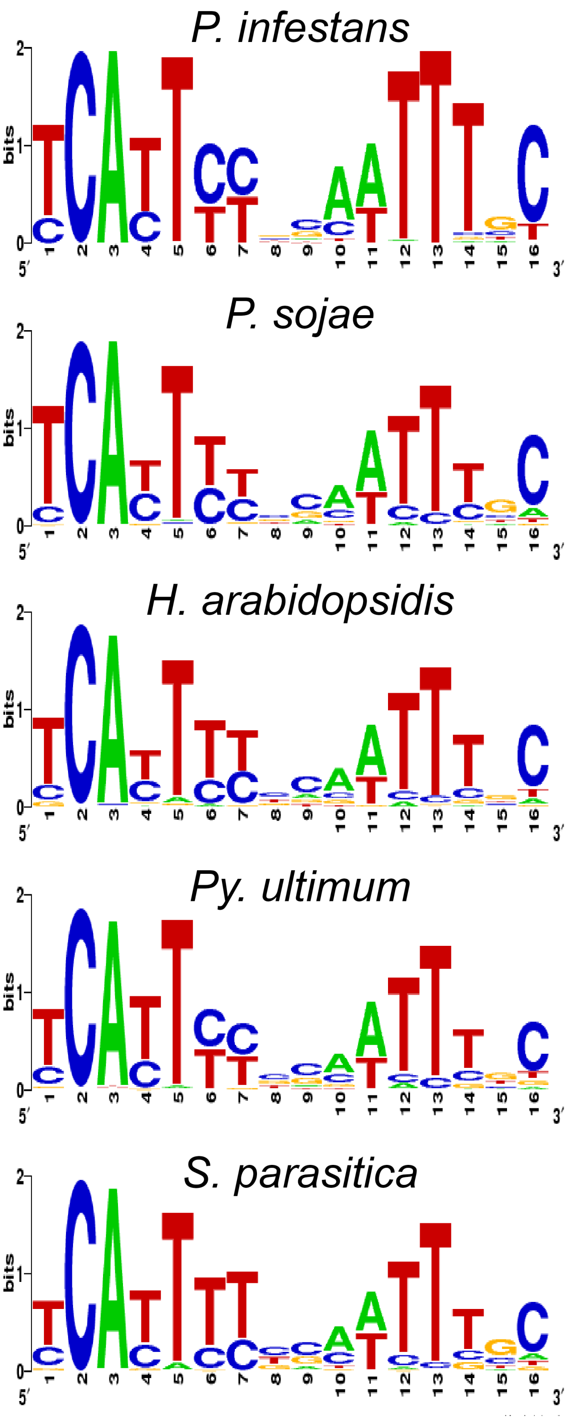

Correlation analysis of microarray and qRT-PCR data

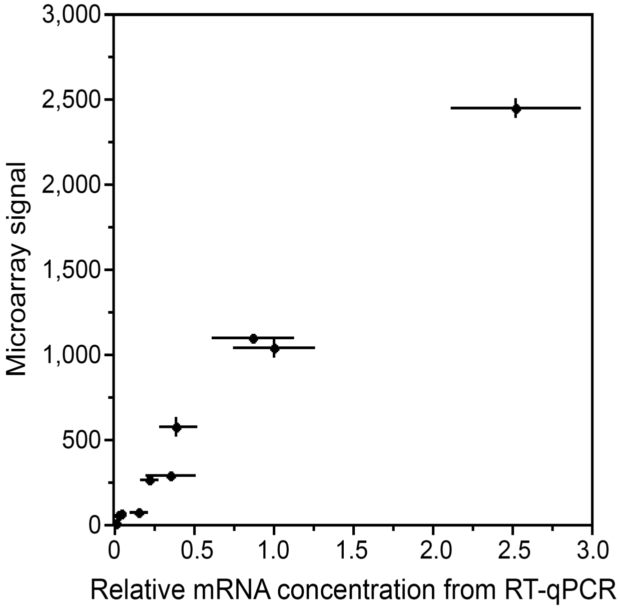

Expression values from Affymetrix arrays compared relative values determined by reverse transcription qPCR. Error bars reflect variation in array data (from duplicates) and qRT-PCR (from triplicates). Genes tested were PITG_00527, PITG_00805, PITG_00988, PITG_03546, PITG_03864, PITG_11913, PITG_15771, PITG_15786, and PITG_16528.
