# Supplementary material for: Novel core promoter elements in the oomycete pathogen Phytophthora infestans and their influence on expression detected by genome-wide analysis
Source: BMC Genomics. 2013 Feb 16;14:106. doi: 10.1186/1471-2164-14-106 (PMC3599244; doi:10.1186/1471-2164-14-106)
Supplement: Additional file 3 — List of genes containing motifs associated with over-represented GO terms. [file 1471-2164-14-106-S3.pdf]

Additional File 3. Genes in over or under GO categories with core promoter motifs.

Promoter class	Over or under-represented?	GO category and genes containing the indicated promoter motif. Functionally redundant categories excluded from Table 1 are included
INR+FPR	Over	GO:0044403<br>PITG_07482, PITG_14788, PITG_13529, PITG_23215, PITG_04378, PITG_09218, PITG_19800, PITG_13481, PITG_15162, PITG_02387, PITG_05068, PITG_06308, PITG_11429, PITG_14787, PITG_18221, PITG_13959, PITG_06050, PITG_22816, PITG_15679, PITG_00582, PITG_08399, PITG_22868, PITG_15166, PITG_22871, PITG_15728, PITG_08070, PITG_22926, PITG_22724, PITG_15255, PITG_16726, PITG_13452, PITG_15341, PITG_18325, PITG_22884, PITG_13093, PITG_22118, PITG_12561, PITG_23074, PITG_23193, PITG_13930, PITG_07597, PITG_16248, PITG_12046, PITG_13119, PITG_22900, PITG_12816, PITG_14783, PITG_23061, PITG_15278, PITG_16874, PITG_22847, PITG_16873, PITG_11484, PITG_13612, PITG_09109, PITG_16705, PITG_14986, PITG_16402, PITG_16243, PITG_22853, PITG_04355, PITG_09316, PITG_15142, PITG_11344, PITG_22547, PITG_04049, PITG_02843, PITG_23069, PITG_04351, PITG_06413, PITG_21778, PITG_16294, PITG_18880, PITG_12952, PITG_12851, PITG_04055, PITG_16872, PITG_23137, PITG_11507, PITG_14984, PITG_02525, PITG_07499, PITG_07555, PITG_05910, PITG_05096, PITG_09111, PITG_05095, PITG_14983, PITG_04148, PITG_16907, PITG_07500, PITG_22676, PITG_11953, PITG_12562, PITG_09216, PITG_09771<br><br>GO:0044419<br>PITG_07482, PITG_14788, PITG_13529, PITG_23215, PITG_04378, PITG_09218, PITG_19800, PITG_13481, PITG_15162, PITG_02387, PITG_05068, PITG_06308, PITG_11429, PITG_14787, PITG_18221, PITG_13959, PITG_06050, PITG_22816, PITG_15679, PITG_00582, PITG_08399, PITG_22868, PITG_15166, PITG_22871, PITG_15728, PITG_08070, PITG_22926, PITG_22724, PITG_15255, PITG_16726, PITG_13452, PITG_15341, PITG_18325, PITG_22884, PITG_13093, PITG_22118, PITG_12561, PITG_23074, PITG_23193, PITG_13930, PITG_07597, PITG_16248, PITG_12046, PITG_13119, PITG_22900, PITG_12816, PITG_14783, PITG_23061, PITG_15278, PITG_16874, PITG_22847, PITG_16873, PITG_11484, PITG_13612,

PITG_09109, PITG_16705, PITG_14986, PITG_16402, PITG_16243, PITG_22853, PITG_04355, PITG_09316, PITG_15142, PITG_11344, PITG_22547, PITG_04049, PITG_02843, PITG_23069, PITG_04351, PITG_06413, PITG_21778, PITG_16294, PITG_18880, PITG_12952, PITG_12851, PITG_04055, PITG_16872, PITG_23137, PITG_11507, PITG_14984, PITG_02525, PITG_07499, PITG_07555, PITG_05910, PITG_05096, PITG_09111, PITG_05095, PITG_14983, PITG_04148, PITG_16907, PITG_07500, PITG_22676, PITG_11953, PITG_12562, PITG_09216, PITG_09771

GO:0051704
PITG_07482, PITG_14788, PITG_13529, PITG_23215, PITG_04378, PITG_09218, PITG_19800, PITG_13481, PITG_15162, PITG_02387, PITG_05068, PITG_06308, PITG_11429, PITG_14787, PITG_18221, PITG_13959, PITG_06050, PITG_22816, PITG_15679, PITG_00582, PITG_08399, PITG_22868, PITG_15166, PITG_22871, PITG_15728, PITG_08070, PITG_22926, PITG_22724, PITG_15255, PITG_16726, PITG_13452, PITG_15341, PITG_18325, PITG_22884, PITG_13093, PITG_22118, PITG_12561, PITG_23074, PITG_23193, PITG_13930, PITG_07597, PITG_16248, PITG_12046, PITG_13119, PITG_22900, PITG_12816, PITG_14783, PITG_23061, PITG_15278, PITG_16874, PITG_22847, PITG_16873, PITG_11484, PITG_13612, PITG_09109, PITG_16705, PITG_14986, PITG_16402, PITG_16243, PITG_22853, PITG_04355, PITG_09316, PITG_15142, PITG_11344, PITG_22547, PITG_04049, PITG_02843, PITG_23069, PITG_04351, PITG_06413, PITG_21778, PITG_16294, PITG_18880, PITG_12952, PITG_12851, PITG_04055, PITG_16872, PITG_23137, PITG_11507, PITG_14984, PITG_02525, PITG_07499, PITG_07555, PITG_05910, PITG_05096, PITG_09111, PITG_05095, PITG_14983, PITG_04148, PITG_16907, PITG_07500, PITG_22676, PITG_11953, PITG_12562, PITG_09216, PITG_09771

GO:0051701
PITG_07482, PITG_14788, PITG_13529, PITG_23215, PITG_09218, PITG_19800, PITG_13481, PITG_15162, PITG_02387, PITG_05068, PITG_06308, PITG_11429, PITG_14787, PITG_18221, PITG_13959, PITG_06050, PITG_22816, PITG_15679, PITG_00582, PITG_08399, PITG_22868, PITG_15166, PITG_22871, PITG_15728, PITG_08070, PITG_22926, PITG_22724, PITG_15255, PITG_16726, PITG_13452, PITG_15341, PITG_22884, PITG_18325, PITG_13093, PITG_22118, PITG_23074,

PITG_23193, PITG_13930, PITG_07597, PITG_16248, PITG_12046, PITG_13119,
PITG_22900, PITG_12816, PITG_14783, PITG_23061, PITG_15278, PITG_11484,
PITG_13612, PITG_09109, PITG_16705, PITG_14986, PITG_16402, PITG_16243,
PITG_22853, PITG_04355, PITG_09316, PITG_11344, PITG_15142, PITG_04049,
PITG_22547, PITG_02843, PITG_06413, PITG_23069, PITG_21778, PITG_04351,
PITG_16294, PITG_18880, PITG_12952, PITG_12851, PITG_04055, PITG_23137,
PITG_11507, PITG_14984, PITG_07499, PITG_07555, PITG_05910, PITG_05096,
PITG_09111, PITG_05095, PITG_14983, PITG_04148, PITG_07500, PITG_22676,
PITG_11953, PITG_09216, PITG_09771

GO:0005975
PITG_18230, PITG_21117, PITG_06808, PITG_15791, PITG_15905, PITG_14243,
PITG_04065, PITG_19619, PITG_01269, PITG_17753, PITG_19634, PITG_19620,
PITG_19625, PITG_16991, PITG_18336, PITG_03441, PITG_06562, PITG_01963,
PITG_21248, PITG_03440, PITG_08612, PITG_19623, PITG_09798, PITG_14138,
PITG_19649, PITG_03335, PITG_14060, PITG_18337, PITG_20927, PITG_02335,
PITG_08000, PITG_04125, PITG_01938, PITG_13721, PITG_17497, PITG_16968,
PITG_19985, PITG_19636, PITG_23158, PITG_01395, PITG_19455, PITG_19624,
PITG_18335, PITG_18209, PITG_14141

GO:0007047
PITG_21248, PITG_08910, PITG_19623, PITG_19649, PITG_03335, PITG_19619,
PITG_08863, PITG_19634, PITG_14173, PITG_08911, PITG_19625, PITG_19620,
PITG_19985, PITG_19636, PITG_19624, PITG_19455

GO:0045229
PITG_21248, PITG_08910, PITG_19623, PITG_19649, PITG_03335, PITG_19619,
PITG_08863, PITG_19634, PITG_14173, PITG_08911, PITG_19625, PITG_19620,
PITG_19985, PITG_19636, PITG_19624, PITG_19455

GO:0055085
PITG_03417, PITG_01101, PITG_08780, PITG_20180, PITG_07711, PITG_21188,
PITG_11290, PITG_12998, PITG_13037, PITG_06157, PITG_07661, PITG_01050,

PITG_15276, PITG_03676, PITG_00103, PITG_16383, PITG_06973, PITG_07663, PITG_05385, PITG_21110, PITG_18652, PITG_18767, PITG_02200, PITG_08296, PITG_12311, PITG_05386, PITG_09386, PITG_11641, PITG_04320, PITG_07085, PITG_12215, PITG_15900, PITG_13020, PITG_04468, PITG_05902, PITG_22364, PITG_00773, PITG_01370, PITG_08297

GO:0006200
PITG_07116, PITG_09229, PITG_21022, PITG_17606, PITG_07716, PITG_06862, PITG_08976, PITG_13579, PITG_06863, PITG_02086, PITG_06109, PITG_13576, PITG_02085, PITG_11062, PITG_04149, PITG_15900, PITG_15731, PITG_13575, PITG_15276, PITG_01370, PITG_06860

GO:0046034
PITG_07116, PITG_09229, PITG_21022, PITG_17606, PITG_07716, PITG_06862, PITG_08976, PITG_13579, PITG_06863, PITG_02086, PITG_06109, PITG_13576, PITG_02085, PITG_11062, PITG_04149, PITG_13235, PITG_15900, PITG_15731, PITG_14230, PITG_13575, PITG_15276, PITG_01370, PITG_06860, PITG_12940

GO:0004553
PITG_18230, PITG_21248, PITG_06808, PITG_08612, PITG_19623, PITG_09798, PITG_15791, PITG_15905, PITG_19649, PITG_14243, PITG_18337, PITG_19619, PITG_14060, PITG_08000, PITG_19634, PITG_16968, PITG_19620, PITG_19625, PITG_19985, PITG_18336, PITG_16991, PITG_01395, PITG_06562, PITG_19636, PITG_19455, PITG_19624, PITG_18335, PITG_01963, PITG_18209

GO:0016798
PITG_18230, PITG_21248, PITG_06808, PITG_08612, PITG_19623, PITG_09798, PITG_15791, PITG_15905, PITG_19649, PITG_14243, PITG_18337, PITG_19619, PITG_14060, PITG_08000, PITG_19634, PITG_16968, PITG_19620, PITG_19625, PITG_19985, PITG_18336, PITG_16991, PITG_01395, PITG_06562, PITG_19636, PITG_19455, PITG_19624, PITG_18335, PITG_01963, PITG_18209

GO:0004650

PITG_21248, PITG_19634, PITG_19623, PITG_19985, PITG_19625, PITG_19620, PITG_19649, PITG_19636, PITG_19455, PITG_19624, PITG_19619

GO:0016491
PITG_02607, PITG_03730, PITG_00870, PITG_09454, PITG_18951, PITG_01165, PITG_16005, PITG_17834, PITG_12757, PITG_01253, PITG_17130, PITG_21744, PITG_04065, PITG_17753, PITG_06569, PITG_16072, PITG_15877, PITG_09807, PITG_14698, PITG_01172, PITG_20455, PITG_08845, PITG_11623, PITG_11581, PITG_17302, PITG_11926, PITG_00545, PITG_08178, PITG_08842, PITG_01110, PITG_18927, PITG_04661, PITG_05385, PITG_12037, PITG_16169, PITG_14722, PITG_15359, PITG_03464, PITG_00972, PITG_01407, PITG_03383, PITG_05386, PITG_16083, PITG_08844, PITG_06398, PITG_08843, PITG_18435, PITG_18972, PITG_02925, PITG_01938, PITG_18935, PITG_15878, PITG_07380, PITG_05486, PITG_23158, PITG_01233, PITG_09856, PITG_02935, PITG_08148, PITG_01408, PITG_19162, PITG_11719

GO:0048037
PITG_04661, PITG_03730, PITG_18951, PITG_11474, PITG_16169, PITG_14722, PITG_15359, PITG_01407, PITG_08445, PITG_03383, PITG_21744, PITG_04065, PITG_16083, PITG_17753, PITG_08844, PITG_02925, PITG_08843, PITG_06569, PITG_01188, PITG_01938, PITG_18935, PITG_16072, PITG_06427, PITG_05486, PITG_07380, PITG_08845, PITG_02935, PITG_01233, PITG_23158, PITG_01408, PITG_01110, PITG_08842

GO:0022857
PITG_04482, PITG_00831, PITG_02086, PITG_07711, PITG_21188, PITG_04149, PITG_13235, PITG_11290, PITG_12998, PITG_13037, PITG_06157, PITG_01050, PITG_15276, PITG_03676, PITG_00103, PITG_02175, PITG_16383, PITG_12940, PITG_06973, PITG_21022, PITG_05385, PITG_18652, PITG_17606, PITG_07716, PITG_18767, PITG_02200, PITG_08976, PITG_12311, PITG_05386, PITG_11641, PITG_02085, PITG_06398, PITG_09261, PITG_04320, PITG_15900, PITG_12215, PITG_00185, PITG_13020, PITG_14230, PITG_05902, PITG_22364, PITG_01370

INR+FPR Under

GO:0005515
PITG_18001, PITG_22801, PITG_13377, PITG_00979, PITG_17834, PITG_03428, PITG_14364, PITG_04065, PITG_12138, PITG_08436, PITG_11264, PITG_07452, PITG_14756, PITG_17349, PITG_05356, PITG_00416, PITG_13372, PITG_11856, PITG_22681, PITG_13196, PITG_00391, PITG_06350, PITG_17567, PITG_01692, PITG_07532, PITG_11853, PITG_14942, PITG_01787, PITG_13314, PITG_16311, PITG_21141, PITG_22936, PITG_00594, PITG_07848, PITG_22920, PITG_06393, PITG_07847, PITG_12990, PITG_07096, PITG_11899, PITG_03582, PITG_00185, PITG_12989, PITG_11223, PITG_01369, PITG_11246, PITG_00513, PITG_17384, PITG_16827

GO:0019538
PITG_00999, PITG_07548, PITG_22801, PITG_02580, PITG_05920, PITG_02559, PITG_00594, PITG_11525, PITG_07949, PITG_17153, PITG_18346, PITG_16339, PITG_03305, PITG_14993, PITG_06724, PITG_03582, PITG_00416, PITG_08784, PITG_16759, PITG_06326

GO:0044267
PITG_00999, PITG_07548, PITG_22801, PITG_02580, PITG_05920, PITG_02559, PITG_00594, PITG_11525, PITG_07949, PITG_17153, PITG_18346, PITG_16339, PITG_03305, PITG_14993, PITG_06724, PITG_03582, PITG_00416, PITG_08784, PITG_16759, PITG_06326

GO:0044260
PITG_00999, PITG_07548, PITG_22801, PITG_02580, PITG_05920, PITG_15791, PITG_02559, PITG_11525, PITG_00594, PITG_03335, PITG_07949, PITG_17153, PITG_18346, PITG_18337, PITG_16339, PITG_03305, PITG_14993, PITG_06724, PITG_03582, PITG_00416, PITG_08784, PITG_18336, PITG_16759, PITG_18335, PITG_06326

GO:0043283
PITG_00999, PITG_22801, PITG_15791, PITG_02559, PITG_14364, PITG_16438, PITG_05455, PITG_17153, PITG_16339, PITG_06724, PITG_00416, PITG_08199,

		PITG_16991, PITG_18336, PITG_13196, PITG_05675, PITG_17567, PITG_07548, PITG_03882, PITG_00433, PITG_05309, PITG_03335, PITG_18346, PITG_18337, PITG_14060, PITG_14993, PITG_17391, PITG_00308, PITG_11223, PITG_00513, PITG_03552, PITG_18335
		GO:0043412<br>PITG_02559, PITG_14993, PITG_00999, PITG_07548, PITG_22801, PITG_06724, PITG_18346, PITG_00416, PITG_16339
		GO:0044237<br>PITG_18078, PITG_21117, PITG_03730, PITG_15791, PITG_14364, PITG_08445, PITG_04065, PITG_17153, PITG_02086, PITG_17753, PITG_03305, PITG_13235, PITG_06427, PITG_09807, PITG_15276, PITG_16759, PITG_11926, PITG_12940, PITG_17567, PITG_02580, PITG_21022, PITG_09229, PITG_00433, PITG_03882, PITG_05920, PITG_03440, PITG_17606, PITG_07716, PITG_05309, PITG_00594, PITG_06862, PITG_03335, PITG_06863, PITG_18346, PITG_13579, PITG_06109, PITG_08000, PITG_01938, PITG_14173, PITG_00308, PITG_11223, PITG_18083, PITG_05486, PITG_00272, PITG_00513, PITG_18335, PITG_15903, PITG_06326, PITG_11719, PITG_17844, PITG_00999, PITG_22801, PITG_12598, PITG_00566, PITG_12462, PITG_04232, PITG_02559, PITG_17837, PITG_16438, PITG_11525, PITG_05455, PITG_13576, PITG_16339, PITG_02221, PITG_04149, PITG_06724, PITG_15731, PITG_05356, PITG_00416, PITG_18336, PITG_17845, PITG_08199, PITG_03441, PITG_13575, PITG_13196, PITG_13736, PITG_05675, PITG_21981, PITG_07116, PITG_07548, PITG_12037, PITG_07949, PITG_08976, PITG_18337, PITG_11062, PITG_14993, PITG_02085, PITG_18435, PITG_02925, PITG_01188, PITG_15900, PITG_03582, PITG_17391, PITG_08784, PITG_07380, PITG_14230, PITG_23158, PITG_03552, PITG_01370, PITG_06860
FPR	Over	GO:0005515<br>PITG_08947, PITG_13348, PITG_10310, PITG_07965, PITG_03238, PITG_05634, PITG_00141, PITG_09960, PITG_03907, PITG_10275, PITG_03106, PITG_10199, PITG_15609, PITG_01169, PITG_03887, PITG_10087, PITG_03433, PITG_03670, PITG_00181, PITG_07889, PITG_15062, PITG_20429, PITG_17350, PITG_06871,

PITG_02898, PITG_06121, PITG_02643, PITG_11424, PITG_10485, PITG_18301,
PITG_00762, PITG_11239, PITG_13064, PITG_12633, PITG_19017, PITG_23176,
PITG_18641, PITG_21695, PITG_18623, PITG_19851, PITG_06682, PITG_19220,
PITG_00445, PITG_20590, PITG_02021, PITG_08164, PITG_07024, PITG_12753,
PITG_04644, PITG_14462, PITG_03395, PITG_01643, PITG_07230, PITG_03310,
PITG_05611, PITG_06354, PITG_04470, PITG_00295, PITG_12114, PITG_11854,
PITG_14708, PITG_09709, PITG_15637, PITG_19601, PITG_03453, PITG_14846,
PITG_19179, PITG_20374, PITG_00342, PITG_22692, PITG_03673, PITG_12794,
PITG_06982, PITG_10939, PITG_01942, PITG_13397, PITG_17348, PITG_03401,
PITG_00140, PITG_13999, PITG_20077, PITG_00098, PITG_17763, PITG_18091,
PITG_05681, PITG_02135, PITG_02893, PITG_05134, PITG_01599, PITG_16666,
PITG_09410, PITG_10946, PITG_10668, PITG_11366, PITG_04483, PITG_00134,
PITG_21164, PITG_04664, PITG_02427, PITG_07814, PITG_03505, PITG_18795,
PITG_10343, PITG_01859, PITG_01615, PITG_03475, PITG_06757, PITG_03343,
PITG_06482, PITG_06829, PITG_19609, PITG_03784, PITG_18024, PITG_04709,
PITG_03363, PITG_10200, PITG_05562, PITG_08237, PITG_07324, PITG_01937,
PITG_07285, PITG_11369, PITG_19877, PITG_17072, PITG_08781, PITG_06744,
PITG_03902, PITG_06320, PITG_00626, PITG_10910, PITG_07033, PITG_02520,
PITG_15286, PITG_13779, PITG_11887, PITG_14973, PITG_01374, PITG_20593,
PITG_07868, PITG_11702, PITG_05494, PITG_19537, PITG_10974, PITG_00038,
PITG_18655, PITG_10954, PITG_15793, PITG_01996, PITG_05444, PITG_01283,
PITG_11063, PITG_12280, PITG_03436, PITG_03587, PITG_02568, PITG_21013,
PITG_03142, PITG_16381, PITG_15695, PITG_09241, PITG_12871, PITG_00842,
PITG_13766, PITG_12980, PITG_20038, PITG_00640, PITG_02339, PITG_02007,
PITG_03876, PITG_23191, PITG_16854, PITG_12949, PITG_01868, PITG_00676,
PITG_19146, PITG_07119, PITG_03032, PITG_14220, PITG_21601, PITG_05814,
PITG_11238, PITG_02748, PITG_16735, PITG_01836, PITG_01470, PITG_19203,
PITG_13171, PITG_00804, PITG_06917, PITG_10933, PITG_02620, PITG_17812,
PITG_01535, PITG_06646, PITG_08960, PITG_19590, PITG_13834, PITG_20381,
PITG_03702, PITG_02769, PITG_17273, PITG_03211, PITG_03011, PITG_17703,
PITG_18687, PITG_13918, PITG_12797, PITG_08723, PITG_02733, PITG_17139,
PITG_00983, PITG_01979, PITG_01786, PITG_02161, PITG_07724, PITG_07840,
PITG_04914, PITG_10900, PITG_16002, PITG_19640, PITG_02259, PITG_09752,

PITG_08346, PITG_10517, PITG_08410, PITG_02977, PITG_02855, PITG_19153,
PITG_15469, PITG_04539, PITG_05692, PITG_08394, PITG_11882, PITG_17879,
PITG_21351, PITG_13149, PITG_11750, PITG_01524, PITG_06776, PITG_00612,
PITG_17027, PITG_08996, PITG_12479, PITG_05358, PITG_01750, PITG_12891,
PITG_10830, PITG_00649, PITG_11029, PITG_07934, PITG_18072, PITG_13908,
PITG_01811, PITG_12223, PITG_09351, PITG_10256, PITG_17161, PITG_21171,
PITG_03734, PITG_03819, PITG_06214, PITG_02137, PITG_20197, PITG_08480,
PITG_11013, PITG_11699, PITG_12539, PITG_01228, PITG_03651, PITG_12497,
PITG_05275, PITG_19875, PITG_23160, PITG_07172, PITG_06963, PITG_06980,
PITG_10979, PITG_05670, PITG_19229, PITG_03458, PITG_05552, PITG_01476,
PITG_17982, PITG_13362, PITG_02793, PITG_09992, PITG_19418, PITG_13168,
PITG_14599, PITG_12345, PITG_03652, PITG_07284, PITG_19037, PITG_06378,
PITG_02126, PITG_11019, PITG_06492, PITG_15400, PITG_19118, PITG_00421,
PITG_03818, PITG_07856, PITG_18317, PITG_06793, PITG_13366, PITG_13133,
PITG_12143, PITG_09840, PITG_13562, PITG_00055, PITG_11536, PITG_05843,
PITG_03228, PITG_19205, PITG_00279, PITG_13772, PITG_13438, PITG_11242,
PITG_11857, PITG_13846, PITG_00314, PITG_01325, PITG_04571, PITG_01183,
PITG_01589, PITG_08576, PITG_04917, PITG_13771, PITG_08690, PITG_06802,
PITG_14024, PITG_17996, PITG_12676, PITG_07937, PITG_15876, PITG_18094,
PITG_08216, PITG_21178, PITG_12982, PITG_08755, PITG_20402, PITG_00946,
PITG_11297, PITG_00129, PITG_17249, PITG_16674, PITG_03361, PITG_10446,
PITG_15929, PITG_00330, PITG_01690, PITG_10664, PITG_10921, PITG_18861,
PITG_21843, PITG_12262, PITG_07887, PITG_05335, PITG_06555, PITG_06231,
PITG_05430, PITG_17963, PITG_01342, PITG_17767, PITG_02820, PITG_02522,
PITG_01794, PITG_20332, PITG_00215, PITG_05122, PITG_03390, PITG_14584,
PITG_02495, PITG_13065, PITG_12350, PITG_16223, PITG_03558, PITG_15459,
PITG_08873, PITG_04380, PITG_05631, PITG_15490, PITG_00641, PITG_12327,
PITG_17936, PITG_03939, PITG_13665, PITG_12131, PITG_05655, PITG_01809,
PITG_00728, PITG_01450, PITG_05429, PITG_03610, PITG_04427, PITG_20157,
PITG_14108, PITG_10728, PITG_12959, PITG_09482, PITG_02723, PITG_05224,
PITG_10934, PITG_14992, PITG_21557, PITG_03243, PITG_12110, PITG_11913,
PITG_13827, PITG_11214, PITG_16425, PITG_06521, PITG_02139, PITG_14385,
PITG_03737, PITG_11594, PITG_01623, PITG_00885, PITG_20611, PITG_01557,

PITG_13225, PITG_21598, PITG_20382, PITG_15098, PITG_00903, PITG_15779,
PITG_08711, PITG_14415, PITG_05185, PITG_06740, PITG_10845, PITG_19429,
PITG_06502, PITG_14231, PITG_01479, PITG_01136, PITG_17313, PITG_13146,
PITG_05656, PITG_06457, PITG_10648, PITG_05600, PITG_13321, PITG_12570,
PITG_01487, PITG_04464, PITG_10420, PITG_14847, PITG_12699, PITG_03423,
PITG_01978, PITG_23119, PITG_02822, PITG_15770, PITG_02567, PITG_11069,
PITG_06659, PITG_09464, PITG_16791, PITG_21299, PITG_23178, PITG_20803,
PITG_15069, PITG_14697, PITG_01181, PITG_03396, PITG_07627, PITG_00646,
PITG_19022, PITG_08552, PITG_06114, PITG_23032, PITG_10645, PITG_09556,
PITG_17409, PITG_13376, PITG_21802, PITG_11816, PITG_14889, PITG_03146,
PITG_16817, PITG_06656, PITG_17552, PITG_00964, PITG_00046, PITG_12527,
PITG_11562, PITG_13368, PITG_03548, PITG_15998, PITG_03163, PITG_03959,
PITG_06008, PITG_11735, PITG_15885, PITG_16801, PITG_03259, PITG_05328,
PITG_01652, PITG_17264, PITG_19461, PITG_07058, PITG_03984, PITG_20250,
PITG_03281, PITG_04836, PITG_08587, PITG_03346, PITG_03680, PITG_01755,
PITG_05569, PITG_10311, PITG_01744, PITG_06628, PITG_00053, PITG_10831,
PITG_12108, PITG_13433, PITG_12184, PITG_04529, PITG_12931, PITG_11170,
PITG_12667, PITG_07249, PITG_15924, PITG_00797, PITG_05442, PITG_03206,
PITG_03937, PITG_14656, PITG_00072, PITG_04878, PITG_22122, PITG_17250,
PITG_03655, PITG_17510, PITG_11403, PITG_13903, PITG_13837, PITG_01402,
PITG_14354, PITG_00597, PITG_18542, PITG_00916, PITG_13904, PITG_08189,
PITG_07296, PITG_16093, PITG_01086, PITG_22739, PITG_15480, PITG_01950,
PITG_13768, PITG_21596, PITG_11765, PITG_03856, PITG_06817, PITG_01528,
PITG_01317, PITG_11182, PITG_13079, PITG_12840, PITG_15834, PITG_05558,
PITG_14111, PITG_11020, PITG_09447, PITG_02088, PITG_15553, PITG_09209,
PITG_16742, PITG_07795, PITG_02662, PITG_11485, PITG_00299, PITG_01784,
PITG_10724, PITG_14970, PITG_19078, PITG_10054, PITG_06759, PITG_08316,
PITG_01108, PITG_04535, PITG_00648, PITG_06725, PITG_04857, PITG_00160,
PITG_10870, PITG_08676, PITG_18167, PITG_02597, PITG_08749, PITG_00496,
PITG_17073, PITG_09816, PITG_08459, PITG_03459, PITG_01219, PITG_07865,
PITG_13443, PITG_19607, PITG_05577, PITG_03592, PITG_04175, PITG_09845,
PITG_01953, PITG_03489, PITG_16469, PITG_05990, PITG_13237, PITG_09855,
PITG_13435, PITG_19158, PITG_10265, PITG_09471, PITG_18093, PITG_10168,

PITG_15735, PITG_05305, PITG_17492, PITG_03977, PITG_06344, PITG_03860,
PITG_06281, PITG_17867, PITG_07366, PITG_08559, PITG_10925, PITG_17175,
PITG_18936, PITG_12943, PITG_01640, PITG_13674, PITG_18328, PITG_05376,
PITG_18656, PITG_00714, PITG_02146, PITG_06160, PITG_16114, PITG_12994,
PITG_11206, PITG_11047, PITG_09555, PITG_00256, PITG_16199, PITG_03933,
PITG_10318, PITG_07850, PITG_04561, PITG_16038, PITG_12426, PITG_10969,
PITG_08654, PITG_16247, PITG_05740, PITG_11237, PITG_02550, PITG_20208,
PITG_09362, PITG_20984, PITG_07962, PITG_17975, PITG_11018, PITG_05778,
PITG_20558, PITG_00686, PITG_00808, PITG_08374, PITG_04242, PITG_16059,
PITG_12214, PITG_05635, PITG_14988, PITG_06353, PITG_15608, PITG_11710,
PITG_17607, PITG_00473, PITG_03017, PITG_05799, PITG_06510, PITG_18563,
PITG_12526, PITG_03359, PITG_03671, PITG_08737, PITG_20030, PITG_13292,
PITG_10916, PITG_11723, PITG_02162, PITG_18746, PITG_13441, PITG_10726,
PITG_02586, PITG_09782, PITG_02502, PITG_10761, PITG_18232, PITG_13379,
PITG_06899, PITG_13284, PITG_11416, PITG_00591, PITG_11540, PITG_04598,
PITG_10591, PITG_15477, PITG_04471, PITG_04053, PITG_09585, PITG_00446,
PITG_18129, PITG_17512, PITG_02541, PITG_10092, PITG_08984, PITG_19164,
PITG_13587, PITG_01252, PITG_07702, PITG_12870, PITG_02002, PITG_04511,
PITG_03898, PITG_08294, PITG_12326, PITG_03010, PITG_03744, PITG_13268,
PITG_02133, PITG_12111, PITG_09835, PITG_13396, PITG_06402, PITG_04678,
PITG_10860, PITG_05447, PITG_03252, PITG_15468, PITG_00207, PITG_01156,
PITG_02715, PITG_03240, PITG_17026, PITG_06587, PITG_10344, PITG_08234,
PITG_03803, PITG_18372, PITG_03773, PITG_03594, PITG_14399, PITG_10795,
PITG_08777, PITG_13267, PITG_10930, PITG_14253, PITG_03908, PITG_17647,
PITG_13311, PITG_02143, PITG_17965, PITG_00988, PITG_11080, PITG_15361,
PITG_09744, PITG_13298, PITG_14105, PITG_21528, PITG_10451, PITG_09564,
PITG_10285, PITG_01598, PITG_11123, PITG_01375, PITG_07017, PITG_11578,
PITG_00655, PITG_19055, PITG_19419, PITG_06149, PITG_11886, PITG_00390,
PITG_05338, PITG_16680, PITG_18049, PITG_11363, PITG_18070, PITG_07765,
PITG_13758, PITG_21311, PITG_12307, PITG_00424, PITG_18141, PITG_05495,
PITG_10991, PITG_02589, PITG_11273, PITG_06705, PITG_05691, PITG_12501,
PITG_04722, PITG_16322, PITG_02810, PITG_01550, PITG_18423, PITG_07321,
PITG_01067, PITG_07129, PITG_08441, PITG_00897, PITG_05928, PITG_16043,

PITG_07971, PITG_17329, PITG_01853, PITG_18962, PITG_15408, PITG_10912,
PITG_08540, PITG_17980, PITG_12048, PITG_02608, PITG_01382, PITG_21187,
PITG_20820, PITG_02840, PITG_02067, PITG_11993, PITG_08514, PITG_10193,
PITG_11021, PITG_10082, PITG_00180, PITG_10128, PITG_01756, PITG_18751,
PITG_21330, PITG_00530, PITG_16668, PITG_17154, PITG_02011, PITG_02401,
PITG_18037, PITG_05780, PITG_03320, PITG_02725, PITG_06367, PITG_04359,
PITG_01416, PITG_11476, PITG_19099, PITG_20556, PITG_16346, PITG_05762,
PITG_18457, PITG_08161, PITG_13815, PITG_13212, PITG_12663, PITG_06800,
PITG_19750, PITG_05653, PITG_18461, PITG_00982, PITG_15623, PITG_08437,
PITG_03288, PITG_10440, PITG_05855, PITG_10792, PITG_17559, PITG_07301,
PITG_15071, PITG_11402, PITG_13276, PITG_03770, PITG_05155, PITG_21181,
PITG_13319, PITG_09982, PITG_13405, PITG_08388, PITG_13795, PITG_11215,
PITG_13838, PITG_05525, PITG_17994, PITG_13440, PITG_11169, PITG_00931,
PITG_13745, PITG_15178, PITG_13373, PITG_02678, PITG_04453, PITG_02712,
PITG_08363, PITG_05642, PITG_12226, PITG_09820, PITG_05825, PITG_02591,
PITG_13103, PITG_00746, PITG_14466, PITG_09862, PITG_17452, PITG_15097,
PITG_02724, PITG_13756, PITG_06380, PITG_04700, PITG_06176, PITG_18106,
PITG_08396, PITG_21212, PITG_08607, PITG_10355, PITG_21597, PITG_15863,
PITG_09220, PITG_05317, PITG_16800, PITG_02646, PITG_16984, PITG_08295,
PITG_03645, PITG_09440, PITG_12169, PITG_06425, PITG_11158, PITG_10617,
PITG_13370, PITG_19428, PITG_14625, PITG_06849, PITG_05818, PITG_19114,
PITG_01444, PITG_11660, PITG_08481, PITG_03485, PITG_10321, PITG_16131,
PITG_09964, PITG_03829, PITG_05583, PITG_06588, PITG_13845, PITG_04023,
PITG_01664, PITG_12844, PITG_10068, PITG_23118, PITG_03608, PITG_13801,
PITG_22957, PITG_20689, PITG_00088, PITG_09294, PITG_17396, PITG_07282,
PITG_03103, PITG_05420, PITG_02479, PITG_00770, PITG_19175, PITG_10905,
PITG_13432, PITG_06288, PITG_03202, PITG_06347, PITG_11241, PITG_06405,
PITG_00349, PITG_20087, PITG_04627, PITG_11298, PITG_03812, PITG_14281,
PITG_03328, PITG_00506, PITG_19950, PITG_01663, PITG_13618, PITG_08593,
PITG_20086, PITG_11759, PITG_21988, PITG_18065, PITG_12981, PITG_05003,
PITG_07725, PITG_06919, PITG_09827, PITG_11538, PITG_11762, PITG_07857,
PITG_15959, PITG_14704, PITG_05757, PITG_02196, PITG_05278, PITG_21177,
PITG_02094, PITG_16532, PITG_02546, PITG_14259, PITG_03229, PITG_17292,

PITG_06748, PITG_08048, PITG_00016, PITG_17629, PITG_02573, PITG_22070,
PITG_02055, PITG_13080, PITG_11647, PITG_05448, PITG_09695, PITG_03111,
PITG_10848, PITG_08712, PITG_06833, PITG_07849, PITG_05340, PITG_11011,
PITG_07745, PITG_11966, PITG_16720, PITG_03926, PITG_11593, PITG_03555,
PITG_15748, PITG_00020, PITG_06013, PITG_08851, PITG_18539, PITG_03891,
PITG_13059, PITG_13052, PITG_03154, PITG_16068, PITG_02778, PITG_06945,
PITG_03562, PITG_08614, PITG_16041, PITG_17986, PITG_02531, PITG_19646,
PITG_17683, PITG_04000, PITG_17180, PITG_14463, PITG_02261, PITG_03998,
PITG_06895, PITG_02796, PITG_16090, PITG_03753, PITG_08819, PITG_11257,
PITG_04156, PITG_13414, PITG_17992, PITG_20249, PITG_04861, PITG_08305,
PITG_11877, PITG_13346, PITG_01003, PITG_02533, PITG_00659, PITG_02304,
PITG_00899, PITG_02113, PITG_19296, PITG_03842, PITG_15175, PITG_04048,
PITG_03207, PITG_17079, PITG_05227, PITG_18016, PITG_15567, PITG_05623,
PITG_11127, PITG_21321, PITG_08310, PITG_11587, PITG_11406, PITG_05989,
PITG_17935, PITG_12239, PITG_15043, PITG_07852, PITG_03014, PITG_12923,
PITG_07270, PITG_18352, PITG_10822, PITG_06384, PITG_05657, PITG_08299,
PITG_02073, PITG_12128, PITG_19213, PITG_07034, PITG_08630, PITG_06600,
PITG_17706, PITG_11531, PITG_15393, PITG_02211, PITG_06120, PITG_03491,
PITG_16671, PITG_04639, PITG_15472, PITG_15262, PITG_06264, PITG_03176,
PITG_17485, PITG_17017, PITG_15960, PITG_06718, PITG_00397, PITG_05480,
PITG_03075, PITG_14137, PITG_20541, PITG_13317, PITG_22989, PITG_22149,
PITG_19166, PITG_05299, PITG_21961, PITG_16818, PITG_02464, PITG_12472,
PITG_06881, PITG_07120, PITG_09664, PITG_17906, PITG_03394, PITG_16864,
PITG_02803, PITG_14991, PITG_09703, PITG_06632, PITG_00263, PITG_00052,
PITG_02789, PITG_03869, PITG_00026, PITG_15054, PITG_16376, PITG_05737,
PITG_21888, PITG_00474, PITG_08826, PITG_06259, PITG_07655, PITG_01603,
PITG_01815, PITG_17882, PITG_10094, PITG_19468, PITG_03683, PITG_10997,
PITG_20985, PITG_20727, PITG_02290, PITG_10287, PITG_03283, PITG_01567,
PITG_03457, PITG_18727, PITG_03547, PITG_07286, PITG_21665, PITG_10760

GO:0003735
PITG_03239, PITG_06636, PITG_14913, PITG_20798, PITG_03178, PITG_01217,
PITG_13371, PITG_06237, PITG_04337, PITG_15090, PITG_03294, PITG_16198,

PITG_03768, PITG_20116, PITG_11923, PITG_13681, PITG_00523, PITG_04843, PITG_01922, PITG_07300, PITG_10887, PITG_10263, PITG_08703, PITG_15069, PITG_14729, PITG_17261, PITG_08809, PITG_16008, PITG_07173, PITG_15722, PITG_15723, PITG_07269, PITG_10193, PITG_01833, PITG_13676, PITG_05009, PITG_12947, PITG_22323, PITG_00941, PITG_00631, PITG_06995, PITG_00443, PITG_19007, PITG_18054, PITG_10146, PITG_03486, PITG_19448, PITG_09442, PITG_14608, PITG_11734, PITG_03221, PITG_19669, PITG_08834, PITG_14850, PITG_03420, PITG_20795, PITG_11766, PITG_12697, PITG_20118, PITG_11045, PITG_04487, PITG_01943, PITG_06799, PITG_04334, PITG_19280, PITG_02694, PITG_09555, PITG_08959, PITG_20264, PITG_05171, PITG_17785, PITG_19121, PITG_05174, PITG_09540, PITG_13500, PITG_02053, PITG_19999, PITG_11099, PITG_10863, PITG_12745, PITG_03762

GO:0044237
PITG_09547, PITG_14835, PITG_20105, PITG_18296, PITG_11919, PITG_08440, PITG_03698, PITG_10310, PITG_10449, PITG_11460, PITG_11615, PITG_13917, PITG_21501, PITG_10401, PITG_07965, PITG_17673, PITG_02050, PITG_10284, PITG_17999, PITG_11923, PITG_13282, PITG_11603, PITG_16104, PITG_15267, PITG_10087, PITG_03433, PITG_04506, PITG_00218, PITG_03670, PITG_13404, PITG_08001, PITG_14668, PITG_06121, PITG_01553, PITG_03351, PITG_18259, PITG_17945, PITG_03514, PITG_02857, PITG_09884, PITG_07240, PITG_10929, PITG_17782, PITG_01065, PITG_23205, PITG_06421, PITG_19463, PITG_11239, PITG_11157, PITG_10804, PITG_17506, PITG_19851, PITG_21223, PITG_19220, PITG_12274, PITG_16913, PITG_10031, PITG_22523, PITG_10780, PITG_18633, PITG_10725, PITG_12105, PITG_17993, PITG_07024, PITG_02001, PITG_13158, PITG_03624, PITG_13737, PITG_03018, PITG_16213, PITG_04644, PITG_17407, PITG_03855, PITG_01804, PITG_07230, PITG_06354, PITG_12166, PITG_19669, PITG_09296, PITG_20795, PITG_07126, PITG_05307, PITG_11766, PITG_12697, PITG_13755, PITG_03453, PITG_18271, PITG_16446, PITG_04726, PITG_14609, PITG_10623, PITG_08314, PITG_06982, PITG_13397, PITG_03365, PITG_02087, PITG_12259, PITG_02381, PITG_15785, PITG_00098, PITG_03934, PITG_14913, PITG_06273, PITG_16741, PITG_22479, PITG_01217, PITG_05587, PITG_14346, PITG_16138, PITG_10711, PITG_03077, PITG_00273, PITG_17820, PITG_15552,

PITG_10210, PITG_03049, PITG_18788, PITG_05374, PITG_06021, PITG_09415,
PITG_05433, PITG_02867, PITG_21400, PITG_13775, PITG_02360, PITG_08751,
PITG_03043, PITG_03632, PITG_11038, PITG_17929, PITG_05991, PITG_06693,
PITG_11082, PITG_17582, PITG_06482, PITG_00614, PITG_10289, PITG_21057,
PITG_06480, PITG_03291, PITG_01314, PITG_11110, PITG_01006, PITG_04709,
PITG_19318, PITG_05009, PITG_05730, PITG_08669, PITG_11375, PITG_07154,
PITG_23007, PITG_10994, PITG_12160, PITG_11369, PITG_19877, PITG_09304,
PITG_09863, PITG_09243, PITG_05694, PITG_00631, PITG_13735, PITG_06320,
PITG_19550, PITG_18373, PITG_10146, PITG_07141, PITG_13511, PITG_18980,
PITG_14412, PITG_14577, PITG_02116, PITG_17295, PITG_20118, PITG_19537,
PITG_16113, PITG_10974, PITG_08766, PITG_00038, PITG_10922, PITG_10305,
PITG_09387, PITG_06406, PITG_00319, PITG_00254, PITG_02960, PITG_11630,
PITG_06280, PITG_23090, PITG_00715, PITG_21063, PITG_10777, PITG_08414,
PITG_17791, PITG_18701, PITG_12993, PITG_03915, PITG_08696, PITG_12186,
PITG_00683, PITG_20798, PITG_07851, PITG_21626, PITG_15695, PITG_10999,
PITG_17831, PITG_06873, PITG_16102, PITG_08584, PITG_13934, PITG_05302,
PITG_00640, PITG_06852, PITG_18105, PITG_17126, PITG_17501, PITG_02465,
PITG_18263, PITG_08900, PITG_12155, PITG_08383, PITG_07948, PITG_21507,
PITG_11036, PITG_07029, PITG_11238, PITG_10141, PITG_10430, PITG_15602,
PITG_20028, PITG_08760, PITG_16735, PITG_17286, PITG_12094, PITG_07637,
PITG_00804, PITG_04567, PITG_03067, PITG_20195, PITG_20808, PITG_20960,
PITG_08606, PITG_19374, PITG_06917, PITG_03900, PITG_22688, PITG_17678,
PITG_08960, PITG_00691, PITG_15603, PITG_20381, PITG_14885, PITG_02769,
PITG_04226, PITG_10972, PITG_08918, PITG_06428, PITG_07235, PITG_00443,
PITG_17273, PITG_05998, PITG_10163, PITG_17032, PITG_17638, PITG_09442,
PITG_11734, PITG_08753, PITG_17703, PITG_23274, PITG_03576, PITG_08435,
PITG_20607, PITG_10862, PITG_01746, PITG_18687, PITG_10138, PITG_13924,
PITG_03509, PITG_08969, PITG_22165, PITG_12509, PITG_12797, PITG_03306,
PITG_02733, PITG_08991, PITG_09508, PITG_00081, PITG_10003, PITG_22662,
PITG_02077, PITG_17719, PITG_07724, PITG_02053, PITG_02003, PITG_10488,
PITG_15140, PITG_03497, PITG_18056, PITG_03239, PITG_17070, PITG_19640,
PITG_09752, PITG_08410, PITG_20530, PITG_03802, PITG_19153, PITG_05692,
PITG_04539, PITG_21806, PITG_17879, PITG_03768, PITG_05851, PITG_05521,

PITG_17054, PITG_06776, PITG_11959, PITG_02565, PITG_05679, PITG_19535,
PITG_04843, PITG_10707, PITG_18278, PITG_05358, PITG_16556, PITG_02904,
PITG_17312, PITG_04452, PITG_18053, PITG_10830, PITG_10317, PITG_04037,
PITG_06679, PITG_11029, PITG_19780, PITG_07934, PITG_10813, PITG_01245,
PITG_05512, PITG_13908, PITG_18332, PITG_20221, PITG_06016, PITG_02044,
PITG_01619, PITG_10513, PITG_16457, PITG_04858, PITG_03731, PITG_05537,
PITG_00887, PITG_07308, PITG_05346, PITG_07156, PITG_06282, PITG_09669,
PITG_15652, PITG_03734, PITG_00910, PITG_16183, PITG_09413, PITG_12315,
PITG_12304, PITG_15831, PITG_01187, PITG_00187, PITG_11013, PITG_22323,
PITG_00941, PITG_03905, PITG_00912, PITG_08958, PITG_07031, PITG_01260,
PITG_10937, PITG_04614, PITG_09882, PITG_19875, PITG_18570, PITG_09898,
PITG_00352, PITG_23160, PITG_03899, PITG_03634, PITG_13097, PITG_07327,
PITG_19448, PITG_10979, PITG_05552, PITG_14828, PITG_11088, PITG_02786,
PITG_04146, PITG_07165, PITG_04003, PITG_01897, PITG_11093, PITG_20600,
PITG_01180, PITG_14771, PITG_12104, PITG_02527, PITG_02026, PITG_18863,
PITG_02198, PITG_19199, PITG_05483, PITG_10857, PITG_12516, PITG_00036,
PITG_07993, PITG_09400, PITG_16057, PITG_13154, PITG_05007, PITG_08806,
PITG_13024, PITG_06684, PITG_13569, PITG_15104, PITG_15244, PITG_03818,
PITG_15659, PITG_08881, PITG_13133, PITG_04823, PITG_07535, PITG_12143,
PITG_13562, PITG_03274, PITG_09477, PITG_00005, PITG_11536, PITG_00279,
PITG_05597, PITG_13457, PITG_11304, PITG_17009, PITG_18634, PITG_21401,
PITG_07481, PITG_20239, PITG_09375, PITG_02476, PITG_17410, PITG_16916,
PITG_06263, PITG_17948, PITG_03399, PITG_00783, PITG_08725, PITG_19813,
PITG_03398, PITG_13583, PITG_00401, PITG_11793, PITG_02303, PITG_16783,
PITG_09722, PITG_16517, PITG_07636, PITG_16203, PITG_08369, PITG_04992,
PITG_06719, PITG_04522, PITG_08375, PITG_12676, PITG_16794, PITG_02858,
PITG_10008, PITG_23108, PITG_15392, PITG_01215, PITG_07885, PITG_08755,
PITG_03201, PITG_07278, PITG_17919, PITG_16808, PITG_04613, PITG_04296,
PITG_10081, PITG_15003, PITG_00330, PITG_17897, PITG_08888, PITG_17571,
PITG_03020, PITG_10045, PITG_20115, PITG_11569, PITG_04487, PITG_21989,
PITG_02213, PITG_06231, PITG_07845, PITG_05162, PITG_17963, PITG_07233,
PITG_02327, PITG_07551, PITG_21333, PITG_19121, PITG_07823, PITG_13968,
PITG_01717, PITG_10847, PITG_03415, PITG_00215, PITG_13914, PITG_05122,

PITG_02529, PITG_04938, PITG_02495, PITG_14352, PITG_10836, PITG_22124,
PITG_15904, PITG_10454, PITG_15982, PITG_18237, PITG_03799, PITG_11803,
PITG_02697, PITG_22427, PITG_15256, PITG_08957, PITG_07349, PITG_21202,
PITG_09445, PITG_06607, PITG_12577, PITG_04679, PITG_10926, PITG_10104,
PITG_05636, PITG_01809, PITG_10334, PITG_01450, PITG_08703, PITG_16669,
PITG_10270, PITG_05429, PITG_14108, PITG_14729, PITG_14084, PITG_01876,
PITG_02917, PITG_02753, PITG_21557, PITG_14992, PITG_17359, PITG_13416,
PITG_18347, PITG_08809, PITG_02264, PITG_16210, PITG_02666, PITG_04527,
PITG_13761, PITG_13016, PITG_10524, PITG_07269, PITG_21615, PITG_04568,
PITG_02139, PITG_07671, PITG_08761, PITG_06926, PITG_15301, PITG_02423,
PITG_07056, PITG_10219, PITG_19527, PITG_22020, PITG_12947, PITG_21598,
PITG_06358, PITG_01762, PITG_20382, PITG_11159, PITG_15779, PITG_05685,
PITG_10990, PITG_00156, PITG_03486, PITG_05616, PITG_13041, PITG_15016,
PITG_14852, PITG_14380, PITG_08834, PITG_04698, PITG_09851, PITG_08572,
PITG_19429, PITG_15566, PITG_17092, PITG_06706, PITG_13322, PITG_10100,
PITG_04640, PITG_01002, PITG_16603, PITG_13146, PITG_18255, PITG_23065,
PITG_07145, PITG_16055, PITG_14966, PITG_07449, PITG_05171, PITG_13590,
PITG_03740, PITG_00172, PITG_17639, PITG_06546, PITG_06772, PITG_20204,
PITG_01296, PITG_05218, PITG_04464, PITG_02239, PITG_04941, PITG_04611,
PITG_02397, PITG_13680, PITG_03956, PITG_12877, PITG_12699, PITG_14315,
PITG_03423, PITG_03811, PITG_23319, PITG_07910, PITG_05865, PITG_11626,
PITG_07828, PITG_01832, PITG_08808, PITG_02567, PITG_15770, PITG_17585,
PITG_03516, PITG_06814, PITG_01798, PITG_17056, PITG_21299, PITG_12151,
PITG_04724, PITG_00077, PITG_20803, PITG_04261, PITG_02672, PITG_03293,
PITG_15069, PITG_10874, PITG_12471, PITG_09849, PITG_12514, PITG_05632,
PITG_01181, PITG_11116, PITG_14697, PITG_15216, PITG_07984, PITG_00646,
PITG_04910, PITG_02973, PITG_09726, PITG_00543, PITG_10645, PITG_10829,
PITG_22684, PITG_17047, PITG_00183, PITG_17925, PITG_15722, PITG_02210,
PITG_01975, PITG_14889, PITG_02255, PITG_09938, PITG_21504, PITG_00964,
PITG_17552, PITG_12050, PITG_09439, PITG_11562, PITG_12264, PITG_16698,
PITG_02707, PITG_07201, PITG_02921, PITG_01096, PITG_15885, PITG_14563,
PITG_16801, PITG_07701, PITG_10602, PITG_02292, PITG_14608, PITG_18868,
PITG_02291, PITG_19461, PITG_12584, PITG_10911, PITG_07058, PITG_20250,

PITG_23109, PITG_12124, PITG_09702, PITG_11999, PITG_10077, PITG_00028,
PITG_01166, PITG_15661, PITG_06900, PITG_10311, PITG_01744, PITG_10817,
PITG_00558, PITG_03158, PITG_12229, PITG_18099, PITG_05174, PITG_08159,
PITG_05456, PITG_01409, PITG_01290, PITG_07843, PITG_06911, PITG_10831,
PITG_10610, PITG_02498, PITG_03762, PITG_13433, PITG_20681, PITG_21284,
PITG_05696, PITG_12931, PITG_10899, PITG_14325, PITG_06195, PITG_19905,
PITG_01695, PITG_15335, PITG_08955, PITG_00523, PITG_01461, PITG_05318,
PITG_17790, PITG_10519, PITG_03764, PITG_18538, PITG_18303, PITG_14156,
PITG_02288, PITG_02561, PITG_23091, PITG_08599, PITG_21132, PITG_13252,
PITG_06701, PITG_16366, PITG_04618, PITG_04878, PITG_02431, PITG_13014,
PITG_17921, PITG_11437, PITG_04666, PITG_05251, PITG_10557, PITG_03522,
PITG_06950, PITG_16008, PITG_01044, PITG_22058, PITG_13336, PITG_13249,
PITG_01389, PITG_18257, PITG_03901, PITG_02795, PITG_01012, PITG_10778,
PITG_10324, PITG_15001, PITG_06708, PITG_00211, PITG_17021, PITG_10821,
PITG_18542, PITG_11111, PITG_00708, PITG_11121, PITG_05585, PITG_03615,
PITG_08189, PITG_17007, PITG_16526, PITG_06995, PITG_03409, PITG_07797,
PITG_01950, PITG_18054, PITG_17724, PITG_07916, PITG_03856, PITG_06817,
PITG_11907, PITG_01528, PITG_16184, PITG_13079, PITG_15413, PITG_12840,
PITG_07643, PITG_14850, PITG_06889, PITG_02574, PITG_02088, PITG_19458,
PITG_03295, PITG_01235, PITG_15553, PITG_14765, PITG_13277, PITG_00593,
PITG_12845, PITG_00299, PITG_17748, PITG_14970, PITG_06848, PITG_12194,
PITG_09540, PITG_09081, PITG_07731, PITG_03410, PITG_18377, PITG_07999,
PITG_04535, PITG_02905, PITG_02740, PITG_13153, PITG_03778, PITG_10198,
PITG_12454, PITG_07248, PITG_01694, PITG_06260, PITG_10951, PITG_01456,
PITG_20584, PITG_18279, PITG_00448, PITG_03296, PITG_07881, PITG_15892,
PITG_00625, PITG_02597, PITG_20491, PITG_06416, PITG_18007, PITG_09816,
PITG_05479, PITG_17930, PITG_08810, PITG_12140, PITG_19607, PITG_10861,
PITG_18025, PITG_13604, PITG_01848, PITG_08094, PITG_00220, PITG_13860,
PITG_10973, PITG_03681, PITG_08837, PITG_05990, PITG_19096, PITG_10049,
PITG_15053, PITG_14392, PITG_03076, PITG_07943, PITG_12122, PITG_01579,
PITG_12540, PITG_11733, PITG_19158, PITG_15735, PITG_02033, PITG_17492,
PITG_15817, PITG_06002, PITG_16337, PITG_03860, PITG_06688, PITG_10399,
PITG_09282, PITG_21607, PITG_04457, PITG_17057, PITG_07366, PITG_00079,

PITG_05731, PITG_08559, PITG_06379, PITG_09712, PITG_07809, PITG_17195,
PITG_10938, PITG_09412, PITG_18420, PITG_01271, PITG_13636, PITG_05376,
PITG_01514, PITG_02615, PITG_06481, PITG_07440, PITG_01356, PITG_17550,
PITG_04442, PITG_05487, PITG_07312, PITG_05520, PITG_08973, PITG_16114,
PITG_06160, PITG_10516, PITG_02049, PITG_01943, PITG_10903, PITG_11047,
PITG_13724, PITG_09555, PITG_10574, PITG_03933, PITG_03995, PITG_16527,
PITG_02115, PITG_03598, PITG_16038, PITG_12426, PITG_00132, PITG_17583,
PITG_10969, PITG_08654, PITG_00430, PITG_06636, PITG_20055, PITG_20272,
PITG_19314, PITG_11237, PITG_09310, PITG_19886, PITG_02550, PITG_15596,
PITG_15090, PITG_03056, PITG_03483, PITG_07962, PITG_08035, PITG_18297,
PITG_02354, PITG_14620, PITG_16479, PITG_23142, PITG_00686, PITG_02355,
PITG_11033, PITG_02384, PITG_01112, PITG_12933, PITG_19767, PITG_17351,
PITG_23177, PITG_02110, PITG_19679, PITG_06595, PITG_21189, PITG_15529,
PITG_16059, PITG_20465, PITG_12220, PITG_10601, PITG_14988, PITG_10887,
PITG_08553, PITG_02791, PITG_03512, PITG_11710, PITG_17500, PITG_04838,
PITG_18276, PITG_17607, PITG_02621, PITG_08549, PITG_21640, PITG_15382,
PITG_09596, PITG_10486, PITG_07173, PITG_11028, PITG_04110, PITG_10916,
PITG_02162, PITG_23044, PITG_02182, PITG_01833, PITG_14707, PITG_13441,
PITG_04405, PITG_12727, PITG_09701, PITG_01742, PITG_02502, PITG_06015,
PITG_01846, PITG_17091, PITG_14972, PITG_17187, PITG_01851, PITG_02017,
PITG_10195, PITG_01856, PITG_00249, PITG_03493, PITG_05815, PITG_15616,
PITG_09791, PITG_12077, PITG_01526, PITG_17135, PITG_04774, PITG_12275,
PITG_10239, PITG_05594, PITG_18718, PITG_03221, PITG_00296, PITG_15309,
PITG_15000, PITG_01204, PITG_17512, PITG_03554, PITG_03262, PITG_13315,
PITG_10092, PITG_08984, PITG_18553, PITG_13587, PITG_04922, PITG_19148,
PITG_06799, PITG_21397, PITG_21723, PITG_12106, PITG_03898, PITG_10186,
PITG_08959, PITG_20131, PITG_12326, PITG_17035, PITG_04225, PITG_21071,
PITG_12111, PITG_14969, PITG_01480, PITG_13268, PITG_14174, PITG_04678,
PITG_17926, PITG_13174, PITG_21243, PITG_10838, PITG_13551, PITG_02715,
PITG_05365, PITG_02581, PITG_04010, PITG_12646, PITG_01616, PITG_17727,
PITG_05373, PITG_02090, PITG_21214, PITG_12743, PITG_05586, PITG_14322,
PITG_02363, PITG_06587, PITG_08234, PITG_10344, PITG_05850, PITG_13615,
PITG_18372, PITG_07991, PITG_03773, PITG_13752, PITG_15890, PITG_11365,

PITG_14166, PITG_03290, PITG_19692, PITG_09751, PITG_10263, PITG_01769,
PITG_09393, PITG_03498, PITG_14253, PITG_10007, PITG_07157, PITG_17778,
PITG_02143, PITG_00988, PITG_13909, PITG_13658, PITG_06753, PITG_16813,
PITG_00561, PITG_09576, PITG_13298, PITG_14105, PITG_16665, PITG_05391,
PITG_05536, PITG_17071, PITG_07978, PITG_11760, PITG_06930, PITG_19087,
PITG_01624, PITG_07829, PITG_02493, PITG_03419, PITG_06886, PITG_17055,
PITG_17983, PITG_10062, PITG_02711, PITG_12291, PITG_00655, PITG_04747,
PITG_18333, PITG_10595, PITG_03584, PITG_15501, PITG_19055, PITG_00716,
PITG_16977, PITG_17998, PITG_10877, PITG_21568, PITG_05338, PITG_21153,
PITG_04500, PITG_16680, PITG_00080, PITG_18064, PITG_17365, PITG_19693,
PITG_08211, PITG_11045, PITG_17840, PITG_12444, PITG_02136, PITG_04610,
PITG_12916, PITG_13677, PITG_11273, PITG_04113, PITG_00911, PITG_05250,
PITG_07405, PITG_00578, PITG_03472, PITG_11099, PITG_05834, PITG_20970,
PITG_13971, PITG_11189, PITG_15906, PITG_11976, PITG_08570, PITG_03672,
PITG_10932, PITG_14216, PITG_18270, PITG_10652, PITG_13371, PITG_16601,
PITG_07846, PITG_16225, PITG_11468, PITG_08129, PITG_01229, PITG_16806,
PITG_20116, PITG_00896, PITG_13172, PITG_03617, PITG_11567, PITG_04975,
PITG_21395, PITG_14239, PITG_01353, PITG_05928, PITG_17574, PITG_01437,
PITG_20883, PITG_02357, PITG_10998, PITG_07792, PITG_07300, PITG_12864,
PITG_15408, PITG_10480, PITG_12459, PITG_08191, PITG_21148, PITG_14727,
PITG_17990, PITG_18867, PITG_13546, PITG_03678, PITG_18005, PITG_09305,
PITG_04436, PITG_10941, PITG_03838, PITG_06578, PITG_15696, PITG_10193,
PITG_13437, PITG_11607, PITG_03703, PITG_16116, PITG_20377, PITG_10884,
PITG_00180, PITG_16636, PITG_15776, PITG_06697, PITG_09932, PITG_06845,
PITG_05796, PITG_01756, PITG_05618, PITG_03277, PITG_14642, PITG_07099,
PITG_00530, PITG_19007, PITG_02401, PITG_06267, PITG_11142, PITG_06986,
PITG_07191, PITG_17651, PITG_17024, PITG_01498, PITG_14413, PITG_17357,
PITG_04418, PITG_06670, PITG_12293, PITG_17950, PITG_05762, PITG_10032,
PITG_17617, PITG_10139, PITG_05653, PITG_04334, PITG_19280, PITG_13245,
PITG_07839, PITG_21852, PITG_02282, PITG_04954, PITG_18258, PITG_21606,
PITG_20770, PITG_15623, PITG_16912, PITG_08073, PITG_05506, PITG_09789,
PITG_10863, PITG_12745, PITG_01583, PITG_10858, PITG_21582, PITG_10440,
PITG_15139, PITG_05855, PITG_07670, PITG_17559, PITG_17745, PITG_15486,

PITG_09407, PITG_16100, PITG_20661, PITG_01510, PITG_10437, PITG_04337,
PITG_17048, PITG_03101, PITG_11402, PITG_03770, PITG_00577, PITG_02852,
PITG_17516, PITG_08598, PITG_01103, PITG_06279, PITG_04498, PITG_06031,
PITG_11215, PITG_13176, PITG_18437, PITG_01895, PITG_14766, PITG_12675,
PITG_15981, PITG_01946, PITG_00661, PITG_21908, PITG_16018, PITG_19771,
PITG_08236, PITG_05115, PITG_13265, PITG_12226, PITG_13719, PITG_17261,
PITG_05953, PITG_05701, PITG_05825, PITG_03260, PITG_07182, PITG_09286,
PITG_17128, PITG_15097, PITG_02724, PITG_05079, PITG_04700, PITG_03807,
PITG_18518, PITG_18906, PITG_17733, PITG_08061, PITG_10828, PITG_14598,
PITG_04538, PITG_02452, PITG_02224, PITG_03322, PITG_03919, PITG_09220,
PITG_05317, PITG_02594, PITG_03553, PITG_16984, PITG_08295, PITG_06019,
PITG_18649, PITG_10108, PITG_14397, PITG_12053, PITG_18799, PITG_01087,
PITG_11158, PITG_03712, PITG_21393, PITG_06639, PITG_15569, PITG_14807,
PITG_08956, PITG_13486, PITG_13370, PITG_19428, PITG_09594, PITG_00761,
PITG_11619, PITG_18545, PITG_10309, PITG_05826, PITG_20264, PITG_20965,
PITG_02429, PITG_21673, PITG_07549, PITG_04683, PITG_13074, PITG_09964,
PITG_13845, PITG_02671, PITG_10866, PITG_13732, PITG_03608, PITG_16734,
PITG_08681, PITG_02186, PITG_20689, PITG_02032, PITG_16966, PITG_10971,
PITG_06204, PITG_12483, PITG_16333, PITG_11393, PITG_08724, PITG_13681,
PITG_20203, PITG_15057, PITG_00417, PITG_00063, PITG_02479, PITG_00770,
PITG_13432, PITG_17572, PITG_02400, PITG_11752, PITG_12588, PITG_05803,
PITG_19868, PITG_12285, PITG_13129, PITG_06288, PITG_21309, PITG_15774,
PITG_01922, PITG_05405, PITG_17586, PITG_11241, PITG_20087, PITG_21854,
PITG_06201, PITG_04589, PITG_05805, PITG_20086, PITG_14886, PITG_06118,
PITG_09504, PITG_00004, PITG_05240, PITG_07725, PITG_20759, PITG_14123,
PITG_07299, PITG_11674, PITG_16940, PITG_14696, PITG_00636, PITG_06749,
PITG_13676, PITG_04931, PITG_07217, PITG_20772, PITG_04721, PITG_07995,
PITG_00030, PITG_00570, PITG_10445, PITG_13920, PITG_18018, PITG_02949,
PITG_03098, PITG_17053, PITG_02442, PITG_21177, PITG_19939, PITG_09817,
PITG_01700, PITG_02094, PITG_15253, PITG_02546, PITG_06748, PITG_17292,
PITG_21765, PITG_06604, PITG_09778, PITG_03270, PITG_20725, PITG_20034,
PITG_02694, PITG_09260, PITG_02055, PITG_10482, PITG_02745, PITG_11647,
PITG_00178, PITG_03864, PITG_00331, PITG_06794, PITG_02821, PITG_13164,

PITG_00291, PITG_03724, PITG_17785, PITG_13500, PITG_09698, PITG_19999,
PITG_05217, PITG_20640, PITG_08712, PITG_20766, PITG_02054, PITG_08403,
PITG_06222, PITG_14310, PITG_07830, PITG_11966, PITG_05340, PITG_03178,
PITG_07211, PITG_15294, PITG_04663, PITG_16198, PITG_03796, PITG_19557,
PITG_08851, PITG_03936, PITG_00643, PITG_07552, PITG_05853, PITG_01343,
PITG_11204, PITG_02721, PITG_09635, PITG_17683, PITG_05204, PITG_07053,
PITG_02992, PITG_21127, PITG_00113, PITG_00221, PITG_14463, PITG_04851,
PITG_06895, PITG_05245, PITG_20587, PITG_19878, PITG_16904, PITG_03460,
PITG_12518, PITG_19482, PITG_10080, PITG_10443, PITG_05886, PITG_11452,
PITG_06702, PITG_20249, PITG_04861, PITG_08831, PITG_05543, PITG_03039,
PITG_07174, PITG_19932, PITG_19445, PITG_15611, PITG_07062, PITG_00899,
PITG_17165, PITG_14968, PITG_06896, PITG_13905, PITG_11812, PITG_03842,
PITG_02277, PITG_03276, PITG_15175, PITG_18853, PITG_03207, PITG_09131,
PITG_04665, PITG_06352, PITG_17495, PITG_00300, PITG_16276, PITG_14623,
PITG_08002, PITG_05369, PITG_00858, PITG_08765, PITG_12495, PITG_01824,
PITG_05989, PITG_16525, PITG_10112, PITG_10147, PITG_14228, PITG_09885,
PITG_19147, PITG_09550, PITG_00203, PITG_12489, PITG_00230, PITG_01447,
PITG_10822, PITG_13586, PITG_17110, PITG_09524, PITG_18251, PITG_16360,
PITG_14971, PITG_10192, PITG_19213, PITG_01262, PITG_21021, PITG_04011,
PITG_01072, PITG_04448, PITG_04646, PITG_15736, PITG_17706, PITG_15393,
PITG_00142, PITG_09251, PITG_11461, PITG_02211, PITG_14920, PITG_10398,
PITG_17114, PITG_06237, PITG_13165, PITG_03294, PITG_17345, PITG_18354,
PITG_08855, PITG_07275, PITG_00407, PITG_19459, PITG_20161, PITG_08890,
PITG_14566, PITG_21621, PITG_13831, PITG_17252, PITG_00704, PITG_14753,
PITG_09723, PITG_17089, PITG_17584, PITG_01203, PITG_09815, PITG_13398,
PITG_05588, PITG_03347, PITG_00397, PITG_01653, PITG_05992, PITG_01718,
PITG_03075, PITG_08368, PITG_07960, PITG_14137, PITG_18891, PITG_22989,
PITG_12090, PITG_10666, PITG_09588, PITG_10106, PITG_01201, PITG_23006,
PITG_15606, PITG_16495, PITG_03045, PITG_15723, PITG_09664, PITG_12465,
PITG_08014, PITG_19379, PITG_15625, PITG_19718, PITG_14915, PITG_20560,
PITG_15778, PITG_14994, PITG_05548, PITG_22210, PITG_20672, PITG_03016,
PITG_00293, PITG_07210, PITG_13015, PITG_16530, PITG_01867, PITG_16376,
PITG_07866, PITG_00474, PITG_20285, PITG_13040, PITG_04477, PITG_06259,

PITG_03420, PITG_02177, PITG_09926, PITG_00569, PITG_00471, PITG_10119, PITG_02212, PITG_20727, PITG_02797, PITG_03336, PITG_00685, PITG_03941, PITG_10287, PITG_14557, PITG_06274, PITG_03837, PITG_22892, PITG_06783, PITG_10935, PITG_18727, PITG_15884, PITG_07286, PITG_19589, PITG_03696, PITG_00440, PITG_10760, PITG_18360

GO:0050790
PITG_09281, PITG_12676, PITG_03142, PITG_13348, PITG_19886, PITG_07017, PITG_10726, PITG_04634, PITG_13368, PITG_15173, PITG_15924, PITG_16671, PITG_14007, PITG_13320, PITG_16668, PITG_06272, PITG_08711, PITG_01703, PITG_03240, PITG_02487, PITG_06963, PITG_21590, PITG_01640, PITG_05928, PITG_03474, PITG_20152, PITG_08876, PITG_02531, PITG_03161, PITG_09962, PITG_18129, PITG_16680, PITG_13666, PITG_17994, PITG_18731, PITG_17882, PITG_01821, PITG_04878, PITG_18959, PITG_01755, PITG_03489, PITG_00299, PITG_18461, PITG_03831, PITG_13546, PITG_03111, PITG_19150, PITG_08514, PITG_00385, PITG_13386, PITG_01105, PITG_08654, PITG_03081, PITG_17926, PITG_01954, PITG_06833

GO:0044260
PITG_14835, PITG_02905, PITG_11919, PITG_11460, PITG_21501, PITG_13917, PITG_10401, PITG_12454, PITG_07248, PITG_07965, PITG_01694, PITG_02050, PITG_20584, PITG_10284, PITG_18279, PITG_11923, PITG_15892, PITG_11603, PITG_02597, PITG_10087, PITG_03433, PITG_04506, PITG_00218, PITG_05479, PITG_12140, PITG_01848, PITG_14668, PITG_01553, PITG_06121, PITG_18259, PITG_17945, PITG_07240, PITG_13860, PITG_10049, PITG_10929, PITG_14392, PITG_19463, PITG_12122, PITG_19158, PITG_15735, PITG_17492, PITG_21223, PITG_15817, PITG_03860, PITG_06688, PITG_10399, PITG_22523, PITG_10780, PITG_12105, PITG_21607, PITG_17057, PITG_04457, PITG_06379, PITG_16213, PITG_07809, PITG_10938, PITG_18420, PITG_01271, PITG_13636, PITG_06481, PITG_07230, PITG_17550, PITG_06354, PITG_19669, PITG_05487, PITG_11766, PITG_20795, PITG_12697, PITG_06160, PITG_10516, PITG_01943, PITG_18271, PITG_16446, PITG_04726, PITG_11047, PITG_13724, PITG_09555, PITG_14609, PITG_10623, PITG_13397, PITG_17583, PITG_12259, PITG_00098, PITG_06636,

PITG_03934, PITG_14913, PITG_06273, PITG_16741, PITG_22479, PITG_01217,
PITG_15596, PITG_03056, PITG_05587, PITG_15090, PITG_10711, PITG_03077,
PITG_00273, PITG_14620, PITG_16479, PITG_23142, PITG_11033, PITG_02384,
PITG_01112, PITG_19767, PITG_03049, PITG_18788, PITG_19679, PITG_02110,
PITG_21189, PITG_02867, PITG_12220, PITG_21400, PITG_14988, PITG_10887,
PITG_08751, PITG_08553, PITG_04838, PITG_18276, PITG_17607, PITG_08549,
PITG_17929, PITG_15382, PITG_05991, PITG_17582, PITG_00614, PITG_06482,
PITG_06480, PITG_07173, PITG_01314, PITG_11110, PITG_10916, PITG_01006,
PITG_02162, PITG_23044, PITG_01833, PITG_14707, PITG_19318, PITG_05009,
PITG_04405, PITG_07154, PITG_10994, PITG_12160, PITG_05694, PITG_02502,
PITG_00631, PITG_01846, PITG_06320, PITG_13735, PITG_14972, PITG_02017,
PITG_01856, PITG_10146, PITG_15616, PITG_09791, PITG_12077, PITG_01526,
PITG_07141, PITG_04774, PITG_05594, PITG_10239, PITG_03221, PITG_18980,
PITG_00296, PITG_15000, PITG_14577, PITG_02116, PITG_17512, PITG_13315,
PITG_20118, PITG_19537, PITG_10092, PITG_08984, PITG_10974, PITG_04922,
PITG_06799, PITG_03898, PITG_09387, PITG_06406, PITG_00319, PITG_00254,
PITG_02960, PITG_08959, PITG_11630, PITG_06280, PITG_23090, PITG_04225,
PITG_14969, PITG_21071, PITG_01480, PITG_08414, PITG_17791, PITG_18701,
PITG_12993, PITG_03915, PITG_12186, PITG_04678, PITG_17926, PITG_13174,
PITG_10838, PITG_21243, PITG_20798, PITG_07851, PITG_15695, PITG_05365,
PITG_17831, PITG_02581, PITG_06873, PITG_17727, PITG_01616, PITG_12646,
PITG_13934, PITG_00640, PITG_18105, PITG_06587, PITG_08234, PITG_17126,
PITG_13752, PITG_03773, PITG_02465, PITG_15890, PITG_18263, PITG_08900,
PITG_12155, PITG_09751, PITG_10263, PITG_07029, PITG_03498, PITG_07157,
PITG_17778, PITG_08760, PITG_16735, PITG_13658, PITG_12094, PITG_13298,
PITG_07637, PITG_00804, PITG_20808, PITG_20960, PITG_06917, PITG_07978,
PITG_02493, PITG_00691, PITG_15603, PITG_17055, PITG_20381, PITG_10062,
PITG_02711, PITG_02769, PITG_04226, PITG_00655, PITG_06428, PITG_04747,
PITG_07235, PITG_00443, PITG_18333, PITG_15501, PITG_03584, PITG_19055,
PITG_09442, PITG_16977, PITG_11734, PITG_08753, PITG_17703, PITG_23274,
PITG_10877, PITG_05338, PITG_08435, PITG_10862, PITG_01746, PITG_18687,
PITG_18064, PITG_11045, PITG_17840, PITG_12509, PITG_04610, PITG_08991,
PITG_09508, PITG_00081, PITG_10003, PITG_12916, PITG_11273, PITG_22662,

PITG_02077, PITG_07724, PITG_02053, PITG_03472, PITG_11099, PITG_10488,
PITG_15140, PITG_03497, PITG_11976, PITG_03239, PITG_19640, PITG_09752,
PITG_08570, PITG_03672, PITG_10932, PITG_18270, PITG_03802, PITG_10652,
PITG_13371, PITG_16601, PITG_04539, PITG_07846, PITG_16225, PITG_11468,
PITG_17879, PITG_20116, PITG_03768, PITG_13172, PITG_03617, PITG_11567,
PITG_04975, PITG_21395, PITG_05521, PITG_17054, PITG_06776, PITG_02565,
PITG_19535, PITG_04843, PITG_10707, PITG_17574, PITG_18278, PITG_05358,
PITG_16556, PITG_02904, PITG_17312, PITG_10998, PITG_18053, PITG_07300,
PITG_12864, PITG_10830, PITG_10317, PITG_10480, PITG_12459, PITG_21148,
PITG_11029, PITG_19780, PITG_10813, PITG_14727, PITG_17990, PITG_18332,
PITG_01619, PITG_10513, PITG_10941, PITG_00887, PITG_10193, PITG_13437,
PITG_11607, PITG_03703, PITG_06282, PITG_07156, PITG_03734, PITG_10884,
PITG_00910, PITG_16636, PITG_15776, PITG_09932, PITG_01756, PITG_00187,
PITG_00941, PITG_22323, PITG_07099, PITG_07031, PITG_01260, PITG_02401,
PITG_19007, PITG_19875, PITG_00352, PITG_11142, PITG_19448, PITG_10979,
PITG_07191, PITG_17651, PITG_17024, PITG_05552, PITG_14413, PITG_17357,
PITG_04418, PITG_12293, PITG_07165, PITG_05762, PITG_04003, PITG_01897,
PITG_01180, PITG_20600, PITG_05653, PITG_04334, PITG_19280, PITG_19199,
PITG_05483, PITG_21852, PITG_07839, PITG_18258, PITG_10857, PITG_12516,
PITG_21606, PITG_09789, PITG_05007, PITG_10863, PITG_08806, PITG_12745,
PITG_13024, PITG_06684, PITG_10440, PITG_05855, PITG_07670, PITG_09407,
PITG_07535, PITG_04337, PITG_03274, PITG_13562, PITG_00577, PITG_03770,
PITG_02852, PITG_00005, PITG_00279, PITG_06279, PITG_04498, PITG_11304,
PITG_17009, PITG_11215, PITG_07481, PITG_09375, PITG_17410, PITG_16916,
PITG_17948, PITG_03399, PITG_00783, PITG_08725, PITG_03398, PITG_15981,
PITG_00661, PITG_00401, PITG_11793, PITG_21908, PITG_19771, PITG_16783,
PITG_05115, PITG_07636, PITG_17261, PITG_08369, PITG_04992, PITG_07182,
PITG_04522, PITG_08375, PITG_02724, PITG_16794, PITG_10008, PITG_05079,
PITG_15392, PITG_07885, PITG_03807, PITG_17733, PITG_18518, PITG_14598,
PITG_10828, PITG_02452, PITG_07278, PITG_02224, PITG_03322, PITG_15003,
PITG_16984, PITG_17897, PITG_08888, PITG_18649, PITG_18799, PITG_17571,
PITG_01087, PITG_03020, PITG_03712, PITG_21393, PITG_10045, PITG_15569,
PITG_14807, PITG_11569, PITG_04487, PITG_21989, PITG_13370, PITG_06231,

PITG_02213, PITG_05162, PITG_07845, PITG_00761, PITG_11619, PITG_20264,
PITG_21333, PITG_19121, PITG_02429, PITG_07549, PITG_21673, PITG_10847,
PITG_03415, PITG_13914, PITG_09964, PITG_13074, PITG_02529, PITG_04938,
PITG_10836, PITG_02671, PITG_13732, PITG_16734, PITG_03608, PITG_08681,
PITG_02186, PITG_20689, PITG_10971, PITG_12483, PITG_20203, PITG_13681,
PITG_02479, PITG_00063, PITG_03799, PITG_15256, PITG_22427, PITG_00770,
PITG_08957, PITG_17572, PITG_02400, PITG_11752, PITG_12588, PITG_05803,
PITG_06607, PITG_06288, PITG_21309, PITG_15774, PITG_01922, PITG_05405,
PITG_17586, PITG_01809, PITG_10334, PITG_08703, PITG_01450, PITG_20087,
PITG_16669, PITG_14729, PITG_21854, PITG_04589, PITG_20086, PITG_14886,
PITG_02917, PITG_09504, PITG_21557, PITG_00004, PITG_14992, PITG_05240,
PITG_17359, PITG_18347, PITG_08809, PITG_02264, PITG_07725, PITG_16210,
PITG_02666, PITG_11674, PITG_10524, PITG_07269, PITG_04568, PITG_21615,
PITG_06926, PITG_13676, PITG_15301, PITG_02423, PITG_07217, PITG_22020,
PITG_12947, PITG_20772, PITG_06358, PITG_01762, PITG_20382, PITG_07995,
PITG_00030, PITG_18018, PITG_15779, PITG_02442, PITG_05685, PITG_03486,
PITG_00156, PITG_13041, PITG_02094, PITG_14380, PITG_08834, PITG_02546,
PITG_08572, PITG_09851, PITG_15566, PITG_17092, PITG_17292, PITG_10100,
PITG_04640, PITG_01002, PITG_16603, PITG_02694, PITG_18255, PITG_09260,
PITG_02055, PITG_00178, PITG_16055, PITG_00331, PITG_02821, PITG_13164,
PITG_05171, PITG_17785, PITG_13500, PITG_19999, PITG_08712, PITG_20766,
PITG_00172, PITG_06222, PITG_07830, PITG_11966, PITG_05340, PITG_20204,
PITG_03178, PITG_04611, PITG_02397, PITG_13680, PITG_03956, PITG_16198,
PITG_03796, PITG_19557, PITG_12699, PITG_08851, PITG_00643, PITG_23319,
PITG_05865, PITG_07910, PITG_11626, PITG_07828, PITG_01832, PITG_01343,
PITG_11204, PITG_02721, PITG_02567, PITG_17585, PITG_17683, PITG_01798,
PITG_12151, PITG_21299, PITG_17056, PITG_04724, PITG_00077, PITG_02992,
PITG_02672, PITG_03293, PITG_15069, PITG_10874, PITG_14463, PITG_05632,
PITG_15216, PITG_20587, PITG_00646, PITG_19878, PITG_04910, PITG_16904,
PITG_10645, PITG_22684, PITG_10829, PITG_03460, PITG_15722, PITG_05886,
PITG_20249, PITG_09938, PITG_21504, PITG_19445, PITG_15611, PITG_07062,
PITG_00899, PITG_17165, PITG_14968, PITG_02707, PITG_06896, PITG_02921,
PITG_07201, PITG_03842, PITG_03276, PITG_17495, PITG_02292, PITG_16276,

PITG_14608, PITG_02291, PITG_19461, PITG_20250, PITG_23109, PITG_12124, PITG_00858, PITG_01824, PITG_11999, PITG_00028, PITG_10147, PITG_14228, PITG_06900, PITG_00203, PITG_00230, PITG_01447, PITG_18099, PITG_10822, PITG_05174, PITG_01409, PITG_18251, PITG_01290, PITG_06911, PITG_16360, PITG_14971, PITG_10831, PITG_10192, PITG_19213, PITG_01262, PITG_04011, PITG_03762, PITG_04448, PITG_21284, PITG_15393, PITG_09251, PITG_11461, PITG_02211, PITG_10899, PITG_06237, PITG_06195, PITG_19905, PITG_03294, PITG_01695, PITG_00407, PITG_19459, PITG_20161, PITG_00523, PITG_08890, PITG_21621, PITG_13831, PITG_17252, PITG_00704, PITG_17584, PITG_01203, PITG_18303, PITG_13398, PITG_00397, PITG_01653, PITG_14156, PITG_02288, PITG_02561, PITG_03075, PITG_08599, PITG_08368, PITG_07960, PITG_14137, PITG_06701, PITG_16366, PITG_02431, PITG_22989, PITG_12090, PITG_13014, PITG_10666, PITG_05251, PITG_03522, PITG_23006, PITG_16008, PITG_22058, PITG_01389, PITG_18257, PITG_15723, PITG_03901, PITG_09664, PITG_10778, PITG_01012, PITG_15001, PITG_06708, PITG_10821, PITG_08014, PITG_11111, PITG_20560, PITG_14994, PITG_05548, PITG_17007, PITG_06995, PITG_00293, PITG_03409, PITG_07210, PITG_07797, PITG_16530, PITG_01950, PITG_18054, PITG_07916, PITG_03856, PITG_11907, PITG_06817, PITG_16184, PITG_15413, PITG_13079, PITG_07643, PITG_06889, PITG_14850, PITG_04477, PITG_06259, PITG_03420, PITG_02177, PITG_10119, PITG_02212, PITG_14765, PITG_13277, PITG_12845, PITG_03941, PITG_17748, PITG_14970, PITG_14557, PITG_06274, PITG_03837, PITG_12194, PITG_22892, PITG_09540, PITG_07731, PITG_19589, PITG_03696, PITG_10760, PITG_07999, PITG_18360

GO:0043283
PITG_14835, PITG_02905, PITG_11919, PITG_08440, PITG_10310, PITG_03778, PITG_21501, PITG_13917, PITG_10401, PITG_07248, PITG_01694, PITG_01456, PITG_17673, PITG_02050, PITG_20584, PITG_18279, PITG_15892, PITG_11603, PITG_16104, PITG_09816, PITG_10087, PITG_06416, PITG_03433, PITG_04506, PITG_05479, PITG_03670, PITG_17930, PITG_13404, PITG_10861, PITG_22758, PITG_01848, PITG_14668, PITG_01553, PITG_03351, PITG_18259, PITG_00220, PITG_17945, PITG_03514, PITG_07240, PITG_10973, PITG_13860, PITG_05990, PITG_10049, PITG_10929, PITG_15053, PITG_06421, PITG_03076, PITG_12122,

PITG_01579, PITG_19158, PITG_15735, PITG_17492, PITG_19851, PITG_21223,
PITG_19220, PITG_06002, PITG_16337, PITG_03860, PITG_06688, PITG_10399,
PITG_10780, PITG_18633, PITG_10725, PITG_12105, PITG_07366, PITG_05731,
PITG_08559, PITG_13158, PITG_03624, PITG_06379, PITG_09712, PITG_13737,
PITG_10938, PITG_07809, PITG_16213, PITG_18420, PITG_04644, PITG_09412,
PITG_17407, PITG_01271, PITG_03855, PITG_06481, PITG_07440, PITG_07230,
PITG_06354, PITG_12166, PITG_04442, PITG_07312, PITG_05487, PITG_09296,
PITG_20795, PITG_06160, PITG_16114, PITG_10516, PITG_03453, PITG_18271,
PITG_16446, PITG_10574, PITG_10623, PITG_06982, PITG_16527, PITG_03995,
PITG_16038, PITG_13397, PITG_12426, PITG_17583, PITG_02087, PITG_10969,
PITG_08654, PITG_02381, PITG_15785, PITG_00098, PITG_03934, PITG_06273,
PITG_19314, PITG_16741, PITG_22479, PITG_02550, PITG_15596, PITG_03056,
PITG_05587, PITG_16138, PITG_03483, PITG_08035, PITG_10711, PITG_03077,
PITG_14620, PITG_16479, PITG_17820, PITG_00686, PITG_23142, PITG_15552,
PITG_02384, PITG_01112, PITG_17351, PITG_23177, PITG_03049, PITG_02110,
PITG_19679, PITG_02867, PITG_15529, PITG_05433, PITG_21189, PITG_16059,
PITG_13775, PITG_21400, PITG_08553, PITG_03043, PITG_08751, PITG_02791,
PITG_11710, PITG_17607, PITG_18276, PITG_08549, PITG_11038, PITG_17929,
PITG_21640, PITG_05991, PITG_15382, PITG_16995, PITG_11082, PITG_17582,
PITG_00614, PITG_10289, PITG_21057, PITG_06480, PITG_10486, PITG_01314,
PITG_01006, PITG_04709, PITG_14707, PITG_19318, PITG_04405, PITG_12160,
PITG_11369, PITG_09243, PITG_09701, PITG_01742, PITG_05694, PITG_17091,
PITG_14972, PITG_02017, PITG_01856, PITG_15616, PITG_09791, PITG_01526,
PITG_07141, PITG_10239, PITG_13511, PITG_18980, PITG_00296, PITG_15000,
PITG_15309, PITG_01204, PITG_02116, PITG_17512, PITG_13315, PITG_10092,
PITG_08984, PITG_10974, PITG_13587, PITG_00038, PITG_04922, PITG_06406,
PITG_00254, PITG_02960, PITG_10186, PITG_06280, PITG_23090, PITG_12326,
PITG_04225, PITG_12111, PITG_14969, PITG_21063, PITG_21071, PITG_01480,
PITG_17791, PITG_12993, PITG_12186, PITG_14174, PITG_04678, PITG_17926,
PITG_00683, PITG_10838, PITG_07851, PITG_15695, PITG_05365, PITG_17831,
PITG_02581, PITG_06873, PITG_17727, PITG_12646, PITG_12743, PITG_08584,
PITG_05586, PITG_13934, PITG_05302, PITG_00640, PITG_18105, PITG_13615,
PITG_17126, PITG_03773, PITG_02465, PITG_15890, PITG_18263, PITG_08900,

PITG_14166, PITG_11365, PITG_12155, PITG_03290, PITG_08383, PITG_09751,
PITG_21507, PITG_07029, PITG_03498, PITG_15602, PITG_17778, PITG_02143,
PITG_20028, PITG_00988, PITG_06753, PITG_12094, PITG_07637, PITG_13298,
PITG_20808, PITG_20195, PITG_04567, PITG_14105, PITG_08606, PITG_20960,
PITG_06917, PITG_05391, PITG_05536, PITG_07978, PITG_11760, PITG_19087,
PITG_01624, PITG_07829, PITG_06886, PITG_20381, PITG_17055, PITG_15603,
PITG_08960, PITG_10062, PITG_12291, PITG_14885, PITG_04226, PITG_07235,
PITG_06428, PITG_18333, PITG_03584, PITG_15501, PITG_17273, PITG_05998,
PITG_10163, PITG_17638, PITG_16977, PITG_08753, PITG_23274, PITG_21568,
PITG_10877, PITG_05338, PITG_08435, PITG_04500, PITG_01746, PITG_16680,
PITG_18687, PITG_18064, PITG_17365, PITG_08211, PITG_22165, PITG_17840,
PITG_12797, PITG_03306, PITG_12444, PITG_02733, PITG_04610, PITG_09508,
PITG_00081, PITG_10003, PITG_13677, PITG_11273, PITG_00911, PITG_22662,
PITG_02077, PITG_05250, PITG_07724, PITG_03472, PITG_10488, PITG_15140,
PITG_13971, PITG_15906, PITG_11976, PITG_17070, PITG_19640, PITG_03672,
PITG_08410, PITG_18270, PITG_10652, PITG_19153, PITG_07846, PITG_16225,
PITG_01229, PITG_17879, PITG_00896, PITG_13172, PITG_03617, PITG_11567,
PITG_21395, PITG_05521, PITG_17054, PITG_06776, PITG_11959, PITG_14239,
PITG_01353, PITG_02565, PITG_05679, PITG_05928, PITG_19535, PITG_10707,
PITG_17574, PITG_18278, PITG_01437, PITG_05358, PITG_16556, PITG_02904,
PITG_17312, PITG_10998, PITG_18053, PITG_12864, PITG_10830, PITG_04037,
PITG_12459, PITG_07934, PITG_14727, PITG_17990, PITG_05512, PITG_18867,
PITG_13908, PITG_18332, PITG_02044, PITG_09305, PITG_04436, PITG_10513,
PITG_03838, PITG_10941, PITG_04858, PITG_15696, PITG_07308, PITG_00887,
PITG_03703, PITG_06282, PITG_16116, PITG_20377, PITG_15652, PITG_10884,
PITG_00910, PITG_00180, PITG_16183, PITG_16636, PITG_09413, PITG_15776,
PITG_12304, PITG_09932, PITG_05618, PITG_01756, PITG_00187, PITG_07099,
PITG_03905, PITG_07031, PITG_01260, PITG_02401, PITG_04614, PITG_19875,
PITG_18570, PITG_00352, PITG_23160, PITG_03899, PITG_11142, PITG_13097,
PITG_07327, PITG_10979, PITG_07191, PITG_17024, PITG_14828, PITG_14413,
PITG_11088, PITG_17357, PITG_04418, PITG_12293, PITG_05762, PITG_04003,
PITG_01897, PITG_20600, PITG_17617, PITG_12104, PITG_02527, PITG_05653,
PITG_10169, PITG_19199, PITG_05483, PITG_21852, PITG_18258, PITG_10857,

PITG_12516, PITG_00036, PITG_15623, PITG_13154, PITG_05007, PITG_13024,
PITG_01583, PITG_06684, PITG_15104, PITG_05855, PITG_03818, PITG_07670,
PITG_17559, PITG_09407, PITG_15486, PITG_13133, PITG_16100, PITG_01510,
PITG_20661, PITG_07535, PITG_12143, PITG_11402, PITG_03274, PITG_00005,
PITG_11536, PITG_00279, PITG_06279, PITG_13457, PITG_11304, PITG_17009,
PITG_18634, PITG_21401, PITG_09375, PITG_02476, PITG_17410, PITG_16916,
PITG_06263, PITG_17948, PITG_03399, PITG_00783, PITG_14766, PITG_08725,
PITG_19813, PITG_03398, PITG_00661, PITG_21908, PITG_16783, PITG_08236,
PITG_05115, PITG_16517, PITG_13719, PITG_17261, PITG_05825, PITG_08369,
PITG_04992, PITG_04522, PITG_09286, PITG_03081, PITG_08375, PITG_02724,
PITG_12676, PITG_15097, PITG_16794, PITG_10008, PITG_05079, PITG_04700,
PITG_23108, PITG_01215, PITG_03807, PITG_17733, PITG_08755, PITG_04538,
PITG_03201, PITG_02452, PITG_02224, PITG_07278, PITG_04613, PITG_03322,
PITG_05317, PITG_10081, PITG_00330, PITG_15003, PITG_03553, PITG_16984,
PITG_08295, PITG_08888, PITG_17897, PITG_14397, PITG_18799, PITG_01087,
PITG_17571, PITG_11158, PITG_03712, PITG_18731, PITG_10045, PITG_21989,
PITG_08956, PITG_06231, PITG_07845, PITG_09594, PITG_00761, PITG_17963,
PITG_11619, PITG_05826, PITG_02327, PITG_02429, PITG_21333, PITG_07549,
PITG_07823, PITG_10847, PITG_00215, PITG_09964, PITG_13914, PITG_04938,
PITG_02529, PITG_05122, PITG_13845, PITG_10836, PITG_02671, PITG_13732,
PITG_03608, PITG_16734, PITG_02186, PITG_08681, PITG_10971, PITG_06204,
PITG_08724, PITG_20203, PITG_18237, PITG_00063, PITG_11803, PITG_15256,
PITG_22427, PITG_00770, PITG_08957, PITG_13432, PITG_17572, PITG_02400,
PITG_11752, PITG_12588, PITG_19868, PITG_12285, PITG_06607, PITG_06288,
PITG_21309, PITG_15774, PITG_05405, PITG_17586, PITG_01809, PITG_10334,
PITG_01450, PITG_20087, PITG_16669, PITG_14108, PITG_21854, PITG_04589,
PITG_06201, PITG_20086, PITG_02753, PITG_14886, PITG_22202, PITG_00004,
PITG_14992, PITG_05240, PITG_17359, PITG_18347, PITG_02264, PITG_07725,
PITG_16210, PITG_02666, PITG_04527, PITG_07299, PITG_11674, PITG_13761,
PITG_13016, PITG_04568, PITG_02139, PITG_15301, PITG_19527, PITG_04931,
PITG_07217, PITG_20772, PITG_21598, PITG_06358, PITG_01762, PITG_07995,
PITG_10445, PITG_13920, PITG_02949, PITG_05685, PITG_21177, PITG_03486,
PITG_13041, PITG_15016, PITG_02546, PITG_08572, PITG_17092, PITG_06748,

PITG_06706, PITG_17292, PITG_04640, PITG_01002, PITG_16603, PITG_20725,
PITG_13146, PITG_18255, PITG_09260, PITG_23065, PITG_10482, PITG_11647,
PITG_00178, PITG_07449, PITG_00291, PITG_20766, PITG_02054, PITG_00172,
PITG_08403, PITG_17639, PITG_06546, PITG_06222, PITG_07830, PITG_05340,
PITG_20204, PITG_07211, PITG_04464, PITG_04941, PITG_04611, PITG_04663,
PITG_02397, PITG_12877, PITG_19557, PITG_12699, PITG_00643, PITG_05865,
PITG_07910, PITG_11626, PITG_01832, PITG_11204, PITG_02721, PITG_15770,
PITG_17585, PITG_03516, PITG_21299, PITG_12151, PITG_00077, PITG_20803,
PITG_07053, PITG_02992, PITG_02672, PITG_03293, PITG_10874, PITG_14463,
PITG_09849, PITG_05632, PITG_15216, PITG_12185, PITG_06895, PITG_07984,
PITG_00646, PITG_19878, PITG_04910, PITG_16904, PITG_10645, PITG_22684,
PITG_10829, PITG_03460, PITG_05886, PITG_20249, PITG_04861, PITG_01975,
PITG_08831, PITG_02255, PITG_09938, PITG_21504, PITG_00964, PITG_17552,
PITG_05543, PITG_12050, PITG_07174, PITG_11562, PITG_15611, PITG_17165,
PITG_16698, PITG_14968, PITG_02921, PITG_11812, PITG_07201, PITG_03276,
PITG_15175, PITG_03207, PITG_15885, PITG_09131, PITG_16801, PITG_17495,
PITG_10602, PITG_02292, PITG_18868, PITG_14623, PITG_02291, PITG_12584,
PITG_10911, PITG_05369, PITG_07058, PITG_20250, PITG_23109, PITG_12124,
PITG_00858, PITG_08765, PITG_09702, PITG_01824, PITG_05989, PITG_11999,
PITG_16525, PITG_10147, PITG_10311, PITG_10817, PITG_01744, PITG_03158,
PITG_00558, PITG_00230, PITG_12229, PITG_17110, PITG_05456, PITG_08159,
PITG_09524, PITG_01409, PITG_01290, PITG_14971, PITG_10831, PITG_19213,
PITG_10192, PITG_01262, PITG_04011, PITG_13433, PITG_04448, PITG_17706,
PITG_15393, PITG_11461, PITG_02211, PITG_12931, PITG_10899, PITG_10398,
PITG_17114, PITG_17345, PITG_19905, PITG_16671, PITG_01695, PITG_00407,
PITG_19459, PITG_08890, PITG_14566, PITG_13831, PITG_17252, PITG_00704,
PITG_14753, PITG_03764, PITG_17089, PITG_17584, PITG_01203, PITG_18303,
PITG_13398, PITG_01797, PITG_01653, PITG_14156, PITG_02288, PITG_02561,
PITG_01718, PITG_03075, PITG_08368, PITG_14137, PITG_13252, PITG_18891,
PITG_06701, PITG_16366, PITG_04878, PITG_22989, PITG_02431, PITG_12090,
PITG_04666, PITG_05251, PITG_10557, PITG_09588, PITG_01201, PITG_23006,
PITG_06950, PITG_01044, PITG_22058, PITG_15606, PITG_13336, PITG_01389,
PITG_18257, PITG_02795, PITG_03901, PITG_09664, PITG_10778, PITG_01012,

PITG_15001, PITG_06708, PITG_17021, PITG_08014, PITG_10821, PITG_18542,
PITG_11111, PITG_20560, PITG_05585, PITG_14994, PITG_08189, PITG_17007,
PITG_16526, PITG_03016, PITG_07210, PITG_07797, PITG_13015, PITG_16530,
PITG_01950, PITG_17724, PITG_01867, PITG_07916, PITG_16376, PITG_03856,
PITG_11907, PITG_06817, PITG_16184, PITG_01528, PITG_13079, PITG_12840,
PITG_13040, PITG_04477, PITG_06259, PITG_02574, PITG_02088, PITG_00569,
PITG_10119, PITG_02212, PITG_14765, PITG_20727, PITG_13277, PITG_00685,
PITG_03941, PITG_17748, PITG_14970, PITG_06274, PITG_12194, PITG_22892,
PITG_06848, PITG_07731, PITG_10935, PITG_18727, PITG_07286, PITG_19589,
PITG_03696, PITG_10760, PITG_18360

GO:0044267
PITG_14835, PITG_02905, PITG_11919, PITG_11460, PITG_21501, PITG_10401,
PITG_12454, PITG_07248, PITG_07965, PITG_01694, PITG_20584, PITG_10284,
PITG_18279, PITG_11923, PITG_15892, PITG_11603, PITG_02597, PITG_10087,
PITG_03433, PITG_04506, PITG_00218, PITG_05479, PITG_12140, PITG_01848,
PITG_14668, PITG_01553, PITG_06121, PITG_18259, PITG_13860, PITG_10929,
PITG_10049, PITG_19463, PITG_14392, PITG_12122, PITG_19158, PITG_15735,
PITG_17492, PITG_21223, PITG_15817, PITG_03860, PITG_06688, PITG_10399,
PITG_22523, PITG_10780, PITG_12105, PITG_21607, PITG_17057, PITG_04457,
PITG_06379, PITG_16213, PITG_07809, PITG_10938, PITG_18420, PITG_01271,
PITG_13636, PITG_06481, PITG_07230, PITG_17550, PITG_06354, PITG_19669,
PITG_05487, PITG_11766, PITG_20795, PITG_12697, PITG_06160, PITG_10516,
PITG_01943, PITG_18271, PITG_16446, PITG_04726, PITG_11047, PITG_13724,
PITG_09555, PITG_14609, PITG_10623, PITG_13397, PITG_17583, PITG_12259,
PITG_00098, PITG_06636, PITG_03934, PITG_14913, PITG_06273, PITG_16741,
PITG_22479, PITG_01217, PITG_15596, PITG_03056, PITG_05587, PITG_15090,
PITG_10711, PITG_03077, PITG_00273, PITG_14620, PITG_16479, PITG_23142,
PITG_11033, PITG_02384, PITG_19767, PITG_03049, PITG_18788, PITG_19679,
PITG_02110, PITG_21189, PITG_02867, PITG_12220, PITG_21400, PITG_14988,
PITG_10887, PITG_08751, PITG_08553, PITG_04838, PITG_18276, PITG_17607,
PITG_08549, PITG_17929, PITG_15382, PITG_05991, PITG_17582, PITG_00614,
PITG_06482, PITG_06480, PITG_07173, PITG_11110, PITG_10916, PITG_01314,

PITG_02162, PITG_01006, PITG_23044, PITG_01833, PITG_14707, PITG_19318,
PITG_05009, PITG_04405, PITG_07154, PITG_10994, PITG_12160, PITG_05694,
PITG_02502, PITG_00631, PITG_01846, PITG_06320, PITG_13735, PITG_14972,
PITG_02017, PITG_01856, PITG_10146, PITG_15616, PITG_09791, PITG_12077,
PITG_01526, PITG_07141, PITG_04774, PITG_05594, PITG_10239, PITG_03221,
PITG_18980, PITG_00296, PITG_15000, PITG_14577, PITG_02116, PITG_17512,
PITG_13315, PITG_20118, PITG_19537, PITG_10092, PITG_08984, PITG_10974,
PITG_04922, PITG_06799, PITG_03898, PITG_09387, PITG_06406, PITG_00319,
PITG_00254, PITG_02960, PITG_08959, PITG_11630, PITG_06280, PITG_23090,
PITG_04225, PITG_14969, PITG_21071, PITG_01480, PITG_08414, PITG_17791,
PITG_18701, PITG_12993, PITG_03915, PITG_12186, PITG_04678, PITG_17926,
PITG_13174, PITG_10838, PITG_21243, PITG_20798, PITG_07851, PITG_15695,
PITG_05365, PITG_17831, PITG_02581, PITG_06873, PITG_17727, PITG_01616,
PITG_12646, PITG_13934, PITG_00640, PITG_18105, PITG_06587, PITG_08234,
PITG_17126, PITG_13752, PITG_03773, PITG_02465, PITG_15890, PITG_18263,
PITG_08900, PITG_12155, PITG_09751, PITG_10263, PITG_07029, PITG_03498,
PITG_07157, PITG_17778, PITG_08760, PITG_16735, PITG_13658, PITG_12094,
PITG_13298, PITG_07637, PITG_00804, PITG_20808, PITG_20960, PITG_06917,
PITG_07978, PITG_02493, PITG_00691, PITG_15603, PITG_20381, PITG_10062,
PITG_02711, PITG_02769, PITG_04226, PITG_00655, PITG_04747, PITG_07235,
PITG_00443, PITG_15501, PITG_03584, PITG_19055, PITG_16977, PITG_09442,
PITG_08753, PITG_11734, PITG_23274, PITG_17703, PITG_10877, PITG_05338,
PITG_08435, PITG_10862, PITG_01746, PITG_18687, PITG_18064, PITG_11045,
PITG_17840, PITG_12509, PITG_04610, PITG_08991, PITG_09508, PITG_00081,
PITG_10003, PITG_12916, PITG_11273, PITG_22662, PITG_02077, PITG_07724,
PITG_02053, PITG_11099, PITG_10488, PITG_15140, PITG_03497, PITG_03239,
PITG_19640, PITG_09752, PITG_08570, PITG_03672, PITG_10932, PITG_18270,
PITG_03802, PITG_10652, PITG_13371, PITG_16601, PITG_04539, PITG_07846,
PITG_16225, PITG_11468, PITG_17879, PITG_20116, PITG_03768, PITG_13172,
PITG_03617, PITG_11567, PITG_04975, PITG_21395, PITG_05521, PITG_06776,
PITG_02565, PITG_19535, PITG_04843, PITG_10707, PITG_17574, PITG_18278,
PITG_05358, PITG_16556, PITG_02904, PITG_17312, PITG_10998, PITG_18053,
PITG_07300, PITG_12864, PITG_10830, PITG_10317, PITG_10480, PITG_12459,

PITG_21148, PITG_11029, PITG_19780, PITG_10813, PITG_17990, PITG_01619,
PITG_10513, PITG_10941, PITG_00887, PITG_10193, PITG_13437, PITG_11607,
PITG_03703, PITG_06282, PITG_07156, PITG_03734, PITG_10884, PITG_00910,
PITG_16636, PITG_15776, PITG_09932, PITG_01756, PITG_00187, PITG_00941,
PITG_22323, PITG_07099, PITG_07031, PITG_01260, PITG_02401, PITG_19007,
PITG_19875, PITG_00352, PITG_11142, PITG_19448, PITG_10979, PITG_07191,
PITG_17651, PITG_17024, PITG_05552, PITG_14413, PITG_17357, PITG_04418,
PITG_12293, PITG_07165, PITG_05762, PITG_01897, PITG_01180, PITG_20600,
PITG_05653, PITG_04334, PITG_19280, PITG_19199, PITG_05483, PITG_21852,
PITG_07839, PITG_18258, PITG_10857, PITG_12516, PITG_21606, PITG_09789,
PITG_05007, PITG_10863, PITG_08806, PITG_12745, PITG_13024, PITG_06684,
PITG_10440, PITG_05855, PITG_07670, PITG_09407, PITG_07535, PITG_04337,
PITG_03274, PITG_13562, PITG_00577, PITG_03770, PITG_02852, PITG_00005,
PITG_00279, PITG_06279, PITG_04498, PITG_11304, PITG_17009, PITG_11215,
PITG_07481, PITG_09375, PITG_17410, PITG_16916, PITG_17948, PITG_03399,
PITG_00783, PITG_08725, PITG_15981, PITG_03398, PITG_00661, PITG_00401,
PITG_11793, PITG_21908, PITG_19771, PITG_16783, PITG_05115, PITG_07636,
PITG_17261, PITG_08369, PITG_04992, PITG_07182, PITG_04522, PITG_08375,
PITG_02724, PITG_10008, PITG_15392, PITG_07885, PITG_03807, PITG_17733,
PITG_18518, PITG_14598, PITG_10828, PITG_02452, PITG_07278, PITG_02224,
PITG_03322, PITG_15003, PITG_17897, PITG_08888, PITG_18649, PITG_18799,
PITG_17571, PITG_01087, PITG_03020, PITG_03712, PITG_21393, PITG_10045,
PITG_15569, PITG_14807, PITG_11569, PITG_04487, PITG_21989, PITG_13370,
PITG_06231, PITG_02213, PITG_05162, PITG_07845, PITG_00761, PITG_11619,
PITG_20264, PITG_21333, PITG_19121, PITG_02429, PITG_07549, PITG_21673,
PITG_10847, PITG_03415, PITG_13914, PITG_13074, PITG_02529, PITG_04938,
PITG_10836, PITG_02671, PITG_13732, PITG_16734, PITG_03608, PITG_08681,
PITG_02186, PITG_20689, PITG_10971, PITG_12483, PITG_20203, PITG_13681,
PITG_02479, PITG_00063, PITG_03799, PITG_00770, PITG_22427, PITG_15256,
PITG_08957, PITG_02400, PITG_17572, PITG_11752, PITG_12588, PITG_05803,
PITG_06288, PITG_06607, PITG_15774, PITG_21309, PITG_17586, PITG_05405,
PITG_01922, PITG_01809, PITG_10334, PITG_16669, PITG_08703, PITG_01450,
PITG_20087, PITG_14729, PITG_21854, PITG_04589, PITG_20086, PITG_14886,

PITG_02917, PITG_09504, PITG_21557, PITG_00004, PITG_14992, PITG_05240,
PITG_17359, PITG_18347, PITG_08809, PITG_02264, PITG_07725, PITG_16210,
PITG_02666, PITG_11674, PITG_10524, PITG_07269, PITG_04568, PITG_21615,
PITG_06926, PITG_13676, PITG_15301, PITG_02423, PITG_07217, PITG_22020,
PITG_12947, PITG_20772, PITG_01762, PITG_20382, PITG_07995, PITG_00030,
PITG_18018, PITG_15779, PITG_02442, PITG_05685, PITG_03486, PITG_00156,
PITG_13041, PITG_02094, PITG_14380, PITG_08834, PITG_02546, PITG_08572,
PITG_09851, PITG_15566, PITG_17092, PITG_17292, PITG_10100, PITG_04640,
PITG_01002, PITG_16603, PITG_02694, PITG_18255, PITG_09260, PITG_02055,
PITG_00178, PITG_16055, PITG_00331, PITG_02821, PITG_13164, PITG_05171,
PITG_17785, PITG_13500, PITG_19999, PITG_08712, PITG_20766, PITG_00172,
PITG_06222, PITG_07830, PITG_11966, PITG_05340, PITG_20204, PITG_03178,
PITG_04611, PITG_02397, PITG_13680, PITG_03956, PITG_16198, PITG_03796,
PITG_19557, PITG_12699, PITG_08851, PITG_00643, PITG_23319, PITG_05865,
PITG_07910, PITG_11626, PITG_07828, PITG_01832, PITG_01343, PITG_11204,
PITG_02721, PITG_02567, PITG_17585, PITG_17683, PITG_01798, PITG_12151,
PITG_21299, PITG_17056, PITG_04724, PITG_00077, PITG_02992, PITG_02672,
PITG_03293, PITG_15069, PITG_10874, PITG_14463, PITG_05632, PITG_15216,
PITG_20587, PITG_00646, PITG_19878, PITG_04910, PITG_16904, PITG_10645,
PITG_22684, PITG_10829, PITG_03460, PITG_15722, PITG_05886, PITG_20249,
PITG_09938, PITG_21504, PITG_19445, PITG_15611, PITG_07062, PITG_00899,
PITG_17165, PITG_14968, PITG_02707, PITG_06896, PITG_02921, PITG_07201,
PITG_03842, PITG_03276, PITG_17495, PITG_02292, PITG_16276, PITG_14608,
PITG_02291, PITG_19461, PITG_20250, PITG_23109, PITG_12124, PITG_00858,
PITG_01824, PITG_11999, PITG_00028, PITG_10147, PITG_14228, PITG_06900,
PITG_00203, PITG_00230, PITG_01447, PITG_18099, PITG_10822, PITG_05174,
PITG_01409, PITG_18251, PITG_01290, PITG_06911, PITG_16360, PITG_14971,
PITG_10831, PITG_10192, PITG_19213, PITG_01262, PITG_03762, PITG_04448,
PITG_21284, PITG_15393, PITG_09251, PITG_11461, PITG_02211, PITG_10899,
PITG_06237, PITG_06195, PITG_19905, PITG_03294, PITG_01695, PITG_00407,
PITG_19459, PITG_20161, PITG_00523, PITG_08890, PITG_13831, PITG_17252,
PITG_21621, PITG_00704, PITG_17584, PITG_01203, PITG_18303, PITG_13398,
PITG_00397, PITG_01653, PITG_14156, PITG_02288, PITG_02561, PITG_03075,

PITG_08599, PITG_08368, PITG_07960, PITG_14137, PITG_06701, PITG_16366, PITG_02431, PITG_22989, PITG_12090, PITG_13014, PITG_10666, PITG_05251, PITG_03522, PITG_23006, PITG_16008, PITG_22058, PITG_01389, PITG_18257, PITG_15723, PITG_03901, PITG_09664, PITG_10778, PITG_01012, PITG_15001, PITG_06708, PITG_10821, PITG_08014, PITG_11111, PITG_20560, PITG_14994, PITG_05548, PITG_06995, PITG_00293, PITG_03409, PITG_07210, PITG_07797, PITG_16530, PITG_01950, PITG_18054, PITG_07916, PITG_03856, PITG_11907, PITG_06817, PITG_16184, PITG_15413, PITG_13079, PITG_07643, PITG_06889, PITG_14850, PITG_04477, PITG_06259, PITG_03420, PITG_02177, PITG_10119, PITG_02212, PITG_14765, PITG_13277, PITG_12845, PITG_03941, PITG_17748, PITG_14970, PITG_14557, PITG_06274, PITG_03837, PITG_12194, PITG_22892, PITG_09540, PITG_07731, PITG_19589, PITG_03696, PITG_10760, PITG_07999, PITG_18360

Under GO:0044403

PITG_17309, PITG_23088, PITG_22717, PITG_15318, PITG_16592, PITG_16623, PITG_04266, PITG_18685, PITG_14432, PITG_03192, PITG_19655, PITG_12646, PITG_07634, PITG_14434, PITG_13536, PITG_16282, PITG_22880, PITG_21190, PITG_06246, PITG_15032, PITG_19588, PITG_09586, PITG_18133, PITG_14309, PITG_14932, PITG_22925, PITG_16615, PITG_18986, PITG_15038, PITG_04353, PITG_05146, PITG_04367, PITG_10244, PITG_06432, PITG_15114, PITG_10339, PITG_13045, PITG_04165, PITG_12626, PITG_15930, PITG_16622, PITG_14965, PITG_22828, PITG_08150, PITG_23024, PITG_10640, PITG_13628, PITG_12094, PITG_15556, PITG_14662, PITG_16575, PITG_13593, PITG_12644, PITG_19992, PITG_05133, PITG_07501, PITG_04326, PITG_14955, PITG_12619, PITG_22845, PITG_13306, PITG_22683, PITG_09838, PITG_15287, PITG_10248, PITG_15225, PITG_13535, PITG_22675, PITG_04329, PITG_16598, PITG_12609, PITG_15235, PITG_19803, PITG_07533, PITG_14960, PITG_22754, PITG_12642, PITG_17185, PITG_19528, PITG_04749, PITG_09935, PITG_21739, PITG_16613, PITG_22818, PITG_01905, PITG_10673, PITG_22256, PITG_04279, PITG_05076, PITG_08317, PITG_05771, PITG_04203, PITG_15757, PITG_08193, PITG_17889, PITG_10808, PITG_08624, PITG_19604, PITG_16428, PITG_00707, PITG_04178, PITG_02897, PITG_05043, PITG_06478, PITG_13047, PITG_22929, PITG_21238, PITG_18554,

PITG_08074, PITG_05841, PITG_09622, PITG_22745, PITG_14954, PITG_20171, PITG_22998, PITG_13537, PITG_10639, PITG_23016, PITG_10773, PITG_23087, PITG_19571, PITG_01024, PITG_20413, PITG_11952, PITG_19307, PITG_10341, PITG_10347, PITG_10232, PITG_15297, PITG_23042, PITG_07947, PITG_16844, PITG_09047, PITG_14673, PITG_21034, PITG_23096, PITG_04856, PITG_19373, PITG_09052, PITG_19565, PITG_23175, PITG_10227, PITG_12090, PITG_18823, PITG_23165, PITG_16285, PITG_17670, PITG_01020, PITG_07558, PITG_18144, PITG_16584, PITG_16597, PITG_16515, PITG_15226, PITG_11350, PITG_19318, PITG_13534, PITG_16636, PITG_20997, PITG_20170, PITG_10654, PITG_23117, PITG_07630, PITG_16614, PITG_09213, PITG_15109, PITG_09837, PITG_07773, PITG_16581, PITG_18148, PITG_09915, PITG_04164, PITG_22017, PITG_22856, PITG_18833, PITG_12551, PITG_23095, PITG_14962, PITG_14443, PITG_18670, PITG_03155, PITG_20940, PITG_12721, PITG_10116, PITG_09585, PITG_22820, PITG_12719, PITG_06030, PITG_23132, PITG_08143, PITG_09223, PITG_10540, PITG_16845, PITG_16195, PITG_10396, PITG_12599, PITG_17316, PITG_16619, PITG_16603, PITG_06485, PITG_07587, PITG_10818, PITG_14961, PITG_12074, PITG_12645, PITG_21388, PITG_10348, PITG_22766, PITG_06099, PITG_18820, PITG_15315, PITG_04314, PITG_23125, PITG_08949, PITG_16144, PITG_23000, PITG_09689, PITG_23236, PITG_22804, PITG_14343, PITG_14959, PITG_19589, PITG_01023, PITG_22999, PITG_10772

GO:0044419
PITG_17309, PITG_23088, PITG_22717, PITG_15318, PITG_16592, PITG_16623, PITG_04266, PITG_18685, PITG_14432, PITG_03192, PITG_19655, PITG_12646, PITG_07634, PITG_14434, PITG_13536, PITG_16282, PITG_22880, PITG_21190, PITG_06246, PITG_15032, PITG_19588, PITG_09586, PITG_18133, PITG_14309, PITG_14932, PITG_22925, PITG_16615, PITG_18986, PITG_15038, PITG_04353, PITG_05146, PITG_04367, PITG_10244, PITG_06432, PITG_15114, PITG_10339, PITG_13045, PITG_04165, PITG_12626, PITG_15930, PITG_16622, PITG_14965, PITG_22828, PITG_08150, PITG_23024, PITG_10640, PITG_13628, PITG_12094, PITG_15556, PITG_14662, PITG_16575, PITG_13593, PITG_12644, PITG_19992, PITG_05133, PITG_07501, PITG_04326, PITG_14955, PITG_12619, PITG_22845, PITG_13306, PITG_22683, PITG_09838, PITG_15287, PITG_10248, PITG_15225,

PITG_13535, PITG_22675, PITG_04329, PITG_16598, PITG_12609, PITG_15235, PITG_19803, PITG_07533, PITG_14960, PITG_22754, PITG_12642, PITG_17185, PITG_19528, PITG_04749, PITG_09935, PITG_21739, PITG_16613, PITG_22818, PITG_01905, PITG_10673, PITG_22256, PITG_04279, PITG_05076, PITG_08317, PITG_05771, PITG_04203, PITG_15757, PITG_08193, PITG_17889, PITG_10808, PITG_08624, PITG_19604, PITG_16428, PITG_00707, PITG_04178, PITG_02897, PITG_05043, PITG_06478, PITG_13047, PITG_22929, PITG_21238, PITG_18554, PITG_08074, PITG_05841, PITG_09622, PITG_22745, PITG_14954, PITG_20171, PITG_22998, PITG_13537, PITG_10639, PITG_23016, PITG_10773, PITG_23087, PITG_19571, PITG_01024, PITG_20413, PITG_11952, PITG_19307, PITG_10341, PITG_10347, PITG_10232, PITG_15297, PITG_23042, PITG_07947, PITG_16844, PITG_09047, PITG_14673, PITG_21034, PITG_23096, PITG_04856, PITG_19373, PITG_09052, PITG_19565, PITG_23175, PITG_10227, PITG_12090, PITG_18823, PITG_23165, PITG_16285, PITG_17670, PITG_01020, PITG_07558, PITG_18144, PITG_16584, PITG_16597, PITG_16515, PITG_15226, PITG_11350, PITG_19318, PITG_13534, PITG_16636, PITG_20997, PITG_20170, PITG_10654, PITG_23117, PITG_07630, PITG_16614, PITG_09213, PITG_15109, PITG_09837, PITG_07773, PITG_16581, PITG_18148, PITG_09915, PITG_04164, PITG_22017, PITG_22856, PITG_18833, PITG_12551, PITG_23095, PITG_14962, PITG_14443, PITG_18670, PITG_03155, PITG_20940, PITG_12721, PITG_10116, PITG_09585, PITG_22820, PITG_12719, PITG_06030, PITG_23132, PITG_08143, PITG_09223, PITG_10540, PITG_16845, PITG_16195, PITG_10396, PITG_12599, PITG_17316, PITG_16619, PITG_16603, PITG_06485, PITG_07587, PITG_10818, PITG_14961, PITG_12074, PITG_12645, PITG_21388, PITG_10348, PITG_22766, PITG_06099, PITG_18820, PITG_15315, PITG_04314, PITG_23125, PITG_08949, PITG_16144, PITG_23000, PITG_09689, PITG_23236, PITG_22804, PITG_14343, PITG_14959, PITG_19589, PITG_01023, PITG_22999, PITG_10772

GO:0051704
PITG_17309, PITG_23088, PITG_22717, PITG_15318, PITG_16592, PITG_16623, PITG_04266, PITG_18685, PITG_14432, PITG_03192, PITG_19655, PITG_12646, PITG_07634, PITG_14434, PITG_13536, PITG_16282, PITG_22880, PITG_21190, PITG_06246, PITG_15032, PITG_19588, PITG_09586, PITG_18133, PITG_14309,

PITG_14932, PITG_22925, PITG_16615, PITG_18986, PITG_15038, PITG_04353,
PITG_05146, PITG_04367, PITG_10244, PITG_06432, PITG_15114, PITG_10339,
PITG_13045, PITG_04165, PITG_12626, PITG_15930, PITG_16622, PITG_14965,
PITG_22828, PITG_08150, PITG_23024, PITG_10640, PITG_13628, PITG_12094,
PITG_15556, PITG_14662, PITG_16575, PITG_13593, PITG_12644, PITG_19992,
PITG_05133, PITG_07501, PITG_04326, PITG_14955, PITG_12619, PITG_22845,
PITG_13306, PITG_22683, PITG_09838, PITG_15287, PITG_10248, PITG_15225,
PITG_13535, PITG_22675, PITG_04329, PITG_16598, PITG_12609, PITG_15235,
PITG_19803, PITG_07533, PITG_14960, PITG_22754, PITG_12642, PITG_17185,
PITG_19528, PITG_04749, PITG_09935, PITG_21739, PITG_16613, PITG_22818,
PITG_01905, PITG_10673, PITG_22256, PITG_04279, PITG_05076, PITG_08317,
PITG_05771, PITG_04203, PITG_15757, PITG_08193, PITG_17889, PITG_10808,
PITG_08624, PITG_19604, PITG_16428, PITG_00707, PITG_04178, PITG_02897,
PITG_05043, PITG_06478, PITG_13047, PITG_22929, PITG_21238, PITG_18554,
PITG_08074, PITG_05841, PITG_09622, PITG_22745, PITG_14954, PITG_20171,
PITG_22998, PITG_13537, PITG_10639, PITG_23016, PITG_10773, PITG_23087,
PITG_19571, PITG_01024, PITG_20413, PITG_11952, PITG_19307, PITG_10341,
PITG_10347, PITG_10232, PITG_15297, PITG_23042, PITG_07947, PITG_16844,
PITG_09047, PITG_14673, PITG_21034, PITG_23096, PITG_04856, PITG_19373,
PITG_09052, PITG_19565, PITG_23175, PITG_10227, PITG_12090, PITG_18823,
PITG_23165, PITG_16285, PITG_10695, PITG_17670, PITG_01020, PITG_07558,
PITG_18144, PITG_16584, PITG_16597, PITG_16515, PITG_15226, PITG_11350,
PITG_19318, PITG_13534, PITG_16636, PITG_20997, PITG_20170, PITG_10654,
PITG_23117, PITG_07630, PITG_16614, PITG_09213, PITG_15109, PITG_09837,
PITG_07773, PITG_16581, PITG_18148, PITG_09915, PITG_04164, PITG_22017,
PITG_22856, PITG_18833, PITG_12551, PITG_23095, PITG_14962, PITG_14443,
PITG_18670, PITG_03155, PITG_20940, PITG_12721, PITG_10116, PITG_09585,
PITG_22820, PITG_12719, PITG_06030, PITG_23132, PITG_08143, PITG_09223,
PITG_10540, PITG_16845, PITG_16195, PITG_10396, PITG_12599, PITG_17316,
PITG_16619, PITG_16603, PITG_06485, PITG_07587, PITG_10818, PITG_14961,
PITG_12074, PITG_12645, PITG_21388, PITG_10348, PITG_22766, PITG_06099,
PITG_18820, PITG_15315, PITG_04314, PITG_23125, PITG_08949, PITG_16144,
PITG_23000, PITG_09689, PITG_23236, PITG_22804, PITG_14343, PITG_14959,

PITG_19589, PITG_01023, PITG_22999, PITG_10772

GO:0051701
PITG_17309, PITG_23088, PITG_15318, PITG_16592, PITG_16623, PITG_04266, PITG_18685, PITG_14432, PITG_03192, PITG_19655, PITG_12646, PITG_07634, PITG_14434, PITG_13536, PITG_16282, PITG_22880, PITG_21190, PITG_06246, PITG_15032, PITG_19588, PITG_09586, PITG_18133, PITG_14309, PITG_14932, PITG_22925, PITG_16615, PITG_18986, PITG_15038, PITG_04353, PITG_05146, PITG_04367, PITG_10244, PITG_06432, PITG_15114, PITG_10339, PITG_13045, PITG_04165, PITG_12626, PITG_15930, PITG_16622, PITG_14965, PITG_22828, PITG_08150, PITG_23024, PITG_10640, PITG_13628, PITG_12094, PITG_15556, PITG_14662, PITG_16575, PITG_13593, PITG_12644, PITG_19992, PITG_05133, PITG_07501, PITG_04326, PITG_14955, PITG_12619, PITG_13306, PITG_22683, PITG_09838, PITG_15287, PITG_10248, PITG_15225, PITG_22675, PITG_13535, PITG_04329, PITG_16598, PITG_12609, PITG_15235, PITG_19803, PITG_07533, PITG_14960, PITG_22754, PITG_12642, PITG_17185, PITG_19528, PITG_04749, PITG_09935, PITG_21739, PITG_16613, PITG_22818, PITG_01905, PITG_10673, PITG_22256, PITG_04279, PITG_05076, PITG_08317, PITG_05771, PITG_04203, PITG_15757, PITG_17889, PITG_10808, PITG_08624, PITG_16428, PITG_00707, PITG_04178, PITG_02897, PITG_05043, PITG_06478, PITG_13047, PITG_22929, PITG_21238, PITG_18554, PITG_08074, PITG_05841, PITG_09622, PITG_14954, PITG_20171, PITG_22998, PITG_13537, PITG_10639, PITG_23016, PITG_23087, PITG_19571, PITG_11952, PITG_19307, PITG_10341, PITG_10232, PITG_10347, PITG_15297, PITG_23042, PITG_07947, PITG_16844, PITG_09047, PITG_21034, PITG_14673, PITG_04856, PITG_19373, PITG_09052, PITG_19565, PITG_23175, PITG_10227, PITG_12090, PITG_18823, PITG_23165, PITG_16285, PITG_17670, PITG_07558, PITG_18144, PITG_16584, PITG_16597, PITG_16515, PITG_15226, PITG_11350, PITG_19318, PITG_13534, PITG_16636, PITG_20997, PITG_20170, PITG_10654, PITG_23117, PITG_07630, PITG_16614, PITG_09213, PITG_15109, PITG_09837, PITG_07773, PITG_16581, PITG_18148, PITG_09915, PITG_04164, PITG_22017, PITG_22856, PITG_18833, PITG_14962, PITG_14443, PITG_18670, PITG_03155, PITG_20940, PITG_12721, PITG_10116, PITG_09585, PITG_22820, PITG_12719, PITG_06030, PITG_23132, PITG_09223, PITG_10540, PITG_16845,

PITG_16195, PITG_10396, PITG_17316, PITG_16619, PITG_16603, PITG_06485, PITG_07587, PITG_10818, PITG_14961, PITG_12074, PITG_12645, PITG_21388, PITG_10348, PITG_22766, PITG_06099, PITG_18820, PITG_15315, PITG_04314, PITG_23125, PITG_08949, PITG_16144, PITG_23000, PITG_09689, PITG_22804, PITG_14343, PITG_14959, PITG_19589, PITG_22999

GO:0007047
PITG_13569, PITG_03554, PITG_05079, PITG_19637, PITG_13322, PITG_03512, PITG_03509, PITG_17055, PITG_10999, PITG_02715, PITG_06358, PITG_10255, PITG_14727, PITG_06428, PITG_17945, PITG_16825, PITG_17054, PITG_03472

GO:0045229
PITG_13569, PITG_03554, PITG_05079, PITG_19637, PITG_13322, PITG_03512, PITG_03509, PITG_17055, PITG_10999, PITG_02715, PITG_06358, PITG_10255, PITG_14727, PITG_06428, PITG_17945, PITG_16825, PITG_17054, PITG_03472

GO:0005975
PITG_19484, PITG_03698, PITG_13483, PITG_08881, PITG_13917, PITG_14717, PITG_10951, PITG_02050, PITG_08590, PITG_10284, PITG_17999, PITG_08598, PITG_20005, PITG_09907, PITG_17501, PITG_01471, PITG_13727, PITG_01462, PITG_12675, PITG_21507, PITG_08001, PITG_22758, PITG_13722, PITG_16965, PITG_09393, PITG_16018, PITG_10255, PITG_17945, PITG_13523, PITG_07240, PITG_10553, PITG_05701, PITG_07310, PITG_09476, PITG_09799, PITG_11452, PITG_12339, PITG_17506, PITG_16794, PITG_01391, PITG_05079, PITG_12274, PITG_17055, PITG_10031, PITG_16404, PITG_11767, PITG_06428, PITG_18069, PITG_09872, PITG_18333, PITG_03919, PITG_04665, PITG_09412, PITG_16984, PITG_04077, PITG_06019, PITG_17998, PITG_10846, PITG_02122, PITG_08002, PITG_20607, PITG_21153, PITG_09296, PITG_05307, PITG_03411, PITG_02049, PITG_02575, PITG_03213, PITG_07405, PITG_03598, PITG_16553, PITG_00132, PITG_03472, PITG_08999, PITG_20970, PITG_09964, PITG_04011, PITG_06476, PITG_11189, PITG_11976, PITG_05696, PITG_15093, PITG_09310, PITG_09185, PITG_08610, PITG_09906, PITG_11459, PITG_16966, PITG_18354, PITG_10204, PITG_17054, PITG_10218, PITG_17592, PITG_10210, PITG_01353, PITG_01112,

PITG_11112, PITG_02038, PITG_06021, PITG_15478, PITG_05636, PITG_19637, PITG_21132, PITG_15980, PITG_08191, PITG_17500, PITG_01431, PITG_09389, PITG_14727, PITG_22202, PITG_16995, PITG_21289, PITG_18332, PITG_06016, PITG_19174, PITG_03141, PITG_11993, PITG_09413, PITG_12315, PITG_07056, PITG_17007, PITG_19774, PITG_09304, PITG_03277, PITG_04272, PITG_06358, PITG_06015, PITG_17546, PITG_13867, PITG_01267, PITG_00912, PITG_01484, PITG_19939, PITG_05705, PITG_01700, PITG_17496, PITG_12275, PITG_03996, PITG_14139, PITG_02786, PITG_17512, PITG_04003, PITG_10032, PITG_11093, PITG_06604, PITG_13296, PITG_08027, PITG_04123, PITG_02026, PITG_13245, PITG_02368, PITG_03979, PITG_16825, PITG_09400, PITG_08073, PITG_01949, PITG_16057, PITG_05526

GO:0004553
PITG_13483, PITG_09185, PITG_08610, PITG_09906, PITG_10149, PITG_10284, PITG_20005, PITG_10204, PITG_17054, PITG_17592, PITG_10218, PITG_11112, PITG_09907, PITG_17501, PITG_01471, PITG_01462, PITG_15478, PITG_08001, PITG_22758, PITG_19637, PITG_13722, PITG_16965, PITG_08191, PITG_01431, PITG_17500, PITG_10255, PITG_17945, PITG_22202, PITG_16995, PITG_10553, PITG_18332, PITG_09476, PITG_03141, PITG_11993, PITG_09799, PITG_01855, PITG_16794, PITG_17506, PITG_07365, PITG_01391, PITG_17055, PITG_10031, PITG_04272, PITG_16404, PITG_17546, PITG_13867, PITG_18069, PITG_18333, PITG_09872, PITG_01484, PITG_19939, PITG_04077, PITG_05705, PITG_03996, PITG_08002, PITG_10846, PITG_11374, PITG_03411, PITG_04123, PITG_08027, PITG_17868, PITG_16825, PITG_03213, PITG_01949, PITG_16553, PITG_06476

GO:0016798
PITG_13483, PITG_07211, PITG_09185, PITG_08610, PITG_09906, PITG_10149, PITG_10284, PITG_20005, PITG_10204, PITG_17054, PITG_17592, PITG_10218, PITG_09907, PITG_11112, PITG_17501, PITG_01471, PITG_01462, PITG_15478, PITG_08001, PITG_22758, PITG_19637, PITG_13722, PITG_18891, PITG_16965, PITG_08191, PITG_01431, PITG_17500, PITG_10255, PITG_17945, PITG_22202, PITG_16995, PITG_18332, PITG_10553, PITG_09476, PITG_03141, PITG_11993, PITG_09799, PITG_01855, PITG_16794, PITG_17506, PITG_07365, PITG_01391,

		PITG_17055, PITG_10031, PITG_04272, PITG_16404, PITG_17546, PITG_13867, PITG_18069, PITG_18333, PITG_09872, PITG_01484, PITG_19939, PITG_04077, PITG_05705, PITG_03996, PITG_08002, PITG_10846, PITG_11374, PITG_03143, PITG_03411, PITG_04123, PITG_08027, PITG_17868, PITG_16825, PITG_03213, PITG_16553, PITG_01949, PITG_06476
DPE	Over	GO:0044237<br>PITG_15244, PITG_06929, PITG_07670, PITG_02359, PITG_08440, PITG_11919, PITG_09780, PITG_19041, PITG_17999, PITG_17516, PITG_11603, PITG_04506, PITG_20239, PITG_08358, PITG_12140, PITG_05149, PITG_18025, PITG_08022, PITG_13558, PITG_00571, PITG_15697, PITG_09722, PITG_18392, PITG_02663, PITG_12863, PITG_17261, PITG_01065, PITG_05701, PITG_14392, PITG_00272, PITG_13671, PITG_03845, PITG_01711, PITG_00573, PITG_10008, PITG_02858, PITG_13139, PITG_07055, PITG_17251, PITG_12105, PITG_04457, PITG_19362, PITG_07278, PITG_01318, PITG_03919, PITG_03018, PITG_03553, PITG_05376, PITG_18998, PITG_06707, PITG_20386, PITG_03020, PITG_03660, PITG_20795, PITG_12697, PITG_11569, PITG_16446, PITG_04726, PITG_00761, PITG_04342, PITG_10623, PITG_15100, PITG_07551, PITG_06982, PITG_20965, PITG_19121, PITG_01717, PITG_03234, PITG_00215, PITG_03415, PITG_13074, PITG_18298, PITG_11457, PITG_10866, PITG_09310, PITG_14936, PITG_15057, PITG_07289, PITG_07251, PITG_13235, PITG_01791, PITG_01922, PITG_12220, PITG_03513, PITG_15790, PITG_21022, PITG_10270, PITG_11710, PITG_02621, PITG_08549, PITG_14084, PITG_17929, PITG_17483, PITG_14992, PITG_04289, PITG_02264, PITG_07036, PITG_01146, PITG_00145, PITG_13016, PITG_13980, PITG_14696, PITG_00395, PITG_06749, PITG_16537, PITG_08669, PITG_10219, PITG_04405, PITG_21598, PITG_20760, PITG_18373, PITG_06819, PITG_15616, PITG_01700, PITG_04701, PITG_07841, PITG_08572, PITG_17512, PITG_01045, PITG_15644, PITG_06596, PITG_03262, PITG_06748, PITG_10442, PITG_08823, PITG_12106, PITG_11126, PITG_00254, PITG_06794, PITG_15417, PITG_01300, PITG_11630, PITG_05171, PITG_03724, PITG_17785, PITG_17035, PITG_13500, PITG_10777, PITG_03740, PITG_14639, PITG_01813, PITG_08696, PITG_00172, PITG_04678, PITG_17639, PITG_00693, PITG_07830, PITG_20204, PITG_10999, PITG_04611, PITG_07968, PITG_01616, PITG_21214, PITG_03796, PITG_07022, PITG_01580,

PITG_08900, PITG_06356, PITG_06979, PITG_21127, PITG_07053, PITG_02672, PITG_20103, PITG_05353, PITG_06895, PITG_21308, PITG_00646, PITG_10518, PITG_09891, PITG_03611, PITG_01195, PITG_06753, PITG_04910, PITG_05485, PITG_02210, PITG_18063, PITG_16646, PITG_15786, PITG_04861, PITG_09439, PITG_02493, PITG_18231, PITG_18706, PITG_00952, PITG_13716, PITG_07201, PITG_03614, PITG_08032, PITG_17578, PITG_16801, PITG_17032, PITG_02757, PITG_03793, PITG_12124, PITG_07714, PITG_00080, PITG_02080, PITG_10147, PITG_14228, PITG_15661, PITG_02733, PITG_18337, PITG_02925, PITG_11273, PITG_07400, PITG_14976, PITG_12229, PITG_01290, PITG_11099, PITG_21021, PITG_11189, PITG_04448, PITG_08183, PITG_17706, PITG_08410, PITG_15393, PITG_17395, PITG_02623, PITG_00208, PITG_03961, PITG_21806, PITG_08445, PITG_13997, PITG_17345, PITG_03294, PITG_03768, PITG_03599, PITG_03389, PITG_08736, PITG_14239, PITG_02497, PITG_17942, PITG_11969, PITG_01653, PITG_02114, PITG_20527, PITG_01019, PITG_01955, PITG_12749, PITG_08025, PITG_13014, PITG_14727, PITG_01913, PITG_05381, PITG_07644, PITG_01619, PITG_13249, PITG_01389, PITG_05463, PITG_09664, PITG_06738, PITG_16116, PITG_06708, PITG_20377, PITG_00708, PITG_12315, PITG_03615, PITG_14001, PITG_17508, PITG_05796, PITG_09666, PITG_02401, PITG_00352, PITG_11588, PITG_01453, PITG_01142, PITG_01920, PITG_03295, PITG_01768, PITG_12104, PITG_11139, PITG_13245, PITG_08206, PITG_03511, PITG_18258, PITG_00593, PITG_10287, PITG_12745, PITG_07731, PITG_01583, PITG_18377, PITG_00588, PITG_18360

GO:0009165
PITG_00693, PITG_06749, PITG_21022, PITG_11457, PITG_02359, PITG_10219, PITG_06979, PITG_03615, PITG_03295, PITG_02621, PITG_00571, PITG_11139, PITG_01955, PITG_14936, PITG_01913, PITG_08032, PITG_13235, PITG_18373, PITG_10287, PITG_03740, PITG_21021, PITG_18377

GO:0044238
PITG_15244, PITG_07670, PITG_02359, PITG_08440, PITG_11919, PITG_09780, PITG_19041, PITG_17999, PITG_17516, PITG_11603, PITG_04506, PITG_20239, PITG_08358, PITG_12140, PITG_05149, PITG_18025, PITG_08022, PITG_13558,

PITG_00571, PITG_15697, PITG_18392, PITG_17261, PITG_05701, PITG_14392,
PITG_03845, PITG_01711, PITG_13671, PITG_10008, PITG_02858, PITG_13139,
PITG_07055, PITG_17251, PITG_12105, PITG_04457, PITG_19362, PITG_07278,
PITG_01318, PITG_03919, PITG_03018, PITG_03553, PITG_18998, PITG_06707,
PITG_20386, PITG_03020, PITG_04333, PITG_03660, PITG_20795, PITG_12697,
PITG_11569, PITG_16446, PITG_04726, PITG_00761, PITG_04342, PITG_10623,
PITG_15100, PITG_06982, PITG_19121, PITG_20965, PITG_01717, PITG_03234,
PITG_03415, PITG_00215, PITG_13074, PITG_11457, PITG_15093, PITG_10866,
PITG_09310, PITG_14936, PITG_05413, PITG_15057, PITG_07289, PITG_07251,
PITG_13235, PITG_01922, PITG_12220, PITG_03513, PITG_15790, PITG_21022,
PITG_10270, PITG_11710, PITG_01431, PITG_02621, PITG_08549, PITG_17929,
PITG_17483, PITG_14992, PITG_01266, PITG_04289, PITG_02264, PITG_07036,
PITG_03141, PITG_13016, PITG_13980, PITG_14696, PITG_00395, PITG_06749,
PITG_16537, PITG_10219, PITG_04405, PITG_21598, PITG_01267, PITG_20760,
PITG_18373, PITG_06819, PITG_15616, PITG_01700, PITG_04701, PITG_07841,
PITG_08572, PITG_02332, PITG_17512, PITG_01045, PITG_15644, PITG_06596,
PITG_06748, PITG_10442, PITG_08823, PITG_12106, PITG_06788, PITG_11126,
PITG_15417, PITG_00254, PITG_01300, PITG_05171, PITG_11630, PITG_17785,
PITG_17035, PITG_13500, PITG_10777, PITG_03740, PITG_14639, PITG_01813,
PITG_00172, PITG_04678, PITG_17639, PITG_00693, PITG_07830, PITG_20204,
PITG_04611, PITG_07968, PITG_01616, PITG_21214, PITG_08590, PITG_03796,
PITG_01860, PITG_07022, PITG_01580, PITG_08900, PITG_06356, PITG_06979,
PITG_21127, PITG_07053, PITG_02672, PITG_20103, PITG_05353, PITG_06895,
PITG_00646, PITG_10518, PITG_03611, PITG_01195, PITG_06753, PITG_04910,
PITG_05485, PITG_07310, PITG_02210, PITG_16646, PITG_15786, PITG_04861,
PITG_06892, PITG_09439, PITG_02493, PITG_18231, PITG_18414, PITG_18706,
PITG_00952, PITG_14237, PITG_07201, PITG_03614, PITG_08032, PITG_16801,
PITG_17578, PITG_17032, PITG_02757, PITG_12124, PITG_07714, PITG_02080,
PITG_10147, PITG_14228, PITG_15661, PITG_02733, PITG_18337, PITG_02925,
PITG_11273, PITG_07400, PITG_12229, PITG_01290, PITG_11099, PITG_21021,
PITG_11189, PITG_04448, PITG_08183, PITG_17706, PITG_08410, PITG_15393,
PITG_02623, PITG_00208, PITG_03961, PITG_21806, PITG_08445, PITG_13997,
PITG_17345, PITG_03294, PITG_03768, PITG_03599, PITG_03389, PITG_08736,

PITG_14239, PITG_02497, PITG_17942, PITG_11969, PITG_01653, PITG_02114, PITG_20527, PITG_01019, PITG_01955, PITG_12749, PITG_08025, PITG_13014, PITG_14727, PITG_01913, PITG_05381, PITG_07644, PITG_01619, PITG_01389, PITG_05463, PITG_09664, PITG_06738, PITG_16116, PITG_06708, PITG_20377, PITG_12315, PITG_14001, PITG_03615, PITG_17508, PITG_09666, PITG_02401, PITG_00352, PITG_11588, PITG_01142, PITG_01453, PITG_03295, PITG_01920, PITG_01768, PITG_12104, PITG_11139, PITG_13245, PITG_08206, PITG_18258, PITG_10287, PITG_12745, PITG_07731, PITG_01583, PITG_01398, PITG_18377, PITG_00588, PITG_18360

GO:0045184
PITG_05884, PITG_15761, PITG_17710, PITG_10203, PITG_04335, PITG_01499, PITG_19907, PITG_20193, PITG_11205, PITG_03614, PITG_00062, PITG_03148, PITG_09419, PITG_01470, PITG_14770, PITG_08736, PITG_01171, PITG_13344, PITG_05192, PITG_01280

Under

GO:0051704
PITG_19942, PITG_01020, PITG_04339, PITG_15152, PITG_07947, PITG_15114, PITG_18487, PITG_02860, PITG_09224, PITG_04089, PITG_00707, PITG_15235, PITG_16737, PITG_07569

GO:0044403
PITG_19942, PITG_01020, PITG_04339, PITG_15152, PITG_07947, PITG_15114, PITG_18487, PITG_02860, PITG_09224, PITG_04089, PITG_00707, PITG_15235, PITG_16737, PITG_07569

GO:0044419
PITG_19942, PITG_01020, PITG_04339, PITG_15152, PITG_07947, PITG_15114, PITG_18487, PITG_02860, PITG_09224, PITG_04089, PITG_00707, PITG_15235, PITG_16737, PITG_07569

GO:0051701
PITG_19942, PITG_04339, PITG_15152, PITG_07947, PITG_15114, PITG_18487,

		PITG_02860, PITG_09224, PITG_04089, PITG_00707, PITG_15235, PITG_16737, PITG_07569
CCAAT	Over	GO:0006412<br>PITG_09866, PITG_20798, PITG_15393, PITG_09521, PITG_03178, PITG_12961, PITG_02493, PITG_12947, PITG_12454, PITG_15090, PITG_18518, PITG_22020, PITG_22523, PITG_20689, PITG_03274, PITG_16198, PITG_20116, PITG_03661, PITG_00879, PITG_19007, PITG_18054, PITG_14608, PITG_03221, PITG_09525, PITG_14850, PITG_15566, PITG_12697, PITG_06596, PITG_03398, PITG_10263, PITG_21393, PITG_20118, PITG_06722, PITG_09631, PITG_20824, PITG_09431, PITG_14609, PITG_03353, PITG_09627, PITG_17748, PITG_03916, PITG_20264, PITG_19121, PITG_05354, PITG_05174, PITG_00388, PITG_07173, PITG_19999, PITG_10863, PITG_03915, PITG_07269, PITG_10193
		GO:0044249<br>PITG_20798, PITG_15393, PITG_02740, PITG_03178, PITG_12961, PITG_14920, PITG_22124, PITG_15090, PITG_07098, PITG_12454, PITG_12725, PITG_20689, PITG_03274, PITG_13165, PITG_16198, PITG_20116, PITG_17516, PITG_05851, PITG_10210, PITG_05850, PITG_09092, PITG_05853, PITG_06595, PITG_09525, PITG_09635, PITG_03398, PITG_10263, PITG_03738, PITG_09631, PITG_20824, PITG_00571, PITG_22685, PITG_02663, PITG_13265, PITG_13416, PITG_03353, PITG_20759, PITG_03916, PITG_09576, PITG_09596, PITG_12121, PITG_04708, PITG_07173, PITG_05537, PITG_10193, PITG_07269, PITG_09866, PITG_05127, PITG_09521, PITG_02493, PITG_18706, PITG_17983, PITG_09304, PITG_18518, PITG_22020, PITG_12947, PITG_22523, PITG_17993, PITG_00879, PITG_03661, PITG_07024, PITG_19007, PITG_02455, PITG_18054, PITG_14608, PITG_03221, PITG_12053, PITG_14850, PITG_15566, PITG_06596, PITG_12697, PITG_05522, PITG_20118, PITG_21393, PITG_06722, PITG_04715, PITG_09431, PITG_00471, PITG_07145, PITG_14609, PITG_09627, PITG_17748, PITG_20264, PITG_13586, PITG_19121, PITG_05354, PITG_05174, PITG_00388, PITG_19999, PITG_10863, PITG_09698, PITG_14639, PITG_03915
		GO:0009058

PITG_20798, PITG_15393, PITG_02740, PITG_03178, PITG_12961, PITG_14920,
PITG_22124, PITG_15090, PITG_07098, PITG_12454, PITG_12725, PITG_20689,
PITG_03274, PITG_13165, PITG_16198, PITG_20116, PITG_17516, PITG_05851,
PITG_10210, PITG_05850, PITG_09092, PITG_05853, PITG_06595, PITG_09525,
PITG_18053, PITG_09635, PITG_03398, PITG_10263, PITG_03738, PITG_05177,
PITG_09631, PITG_20824, PITG_22685, PITG_00571, PITG_18583, PITG_02663,
PITG_13265, PITG_13416, PITG_03353, PITG_20759, PITG_03916, PITG_09576,
PITG_09596, PITG_12121, PITG_04708, PITG_07173, PITG_05537, PITG_10193,
PITG_07269, PITG_09866, PITG_05127, PITG_09521, PITG_02493, PITG_18706,
PITG_17983, PITG_09304, PITG_18518, PITG_22020, PITG_12947, PITG_22523,
PITG_03685, PITG_17993, PITG_10028, PITG_03661, PITG_00879, PITG_19007,
PITG_07024, PITG_02455, PITG_18054, PITG_14608, PITG_03221, PITG_12053,
PITG_14850, PITG_15566, PITG_12697, PITG_06596, PITG_05522, PITG_20118,
PITG_21393, PITG_06722, PITG_04715, PITG_09431, PITG_00471, PITG_18255,
PITG_03755, PITG_07145, PITG_18258, PITG_20211, PITG_14609, PITG_15100,
PITG_09627, PITG_17748, PITG_20264, PITG_13586, PITG_19121, PITG_13721,
PITG_05354, PITG_05174, PITG_00388, PITG_09698, PITG_10863, PITG_19999,
PITG_14639, PITG_03915

GO:0010467
PITG_17070, PITG_03934, PITG_20798, PITG_05314, PITG_15393, PITG_17706,
PITG_03178, PITG_12961, PITG_15090, PITG_04663, PITG_12454, PITG_21077,
PITG_16138, PITG_20689, PITG_03274, PITG_17673, PITG_16198, PITG_20116,
PITG_17166, PITG_19868, PITG_09525, PITG_15529, PITG_03398, PITG_10263,
PITG_09631, PITG_20824, PITG_00988, PITG_03353, PITG_05512, PITG_13908,
PITG_15598, PITG_05990, PITG_10289, PITG_03916, PITG_07173, PITG_20195,
PITG_13761, PITG_04858, PITG_10193, PITG_07308, PITG_07269, PITG_00051,
PITG_17021, PITG_09866, PITG_07174, PITG_09521, PITG_02493, PITG_16526,
PITG_18518, PITG_22020, PITG_12947, PITG_18633, PITG_22523, PITG_19362,
PITG_00879, PITG_03661, PITG_19007, PITG_18054, PITG_03855, PITG_04701,
PITG_14608, PITG_13196, PITG_03221, PITG_10444, PITG_14850, PITG_15566,
PITG_06596, PITG_12697, PITG_09405, PITG_20118, PITG_21393, PITG_06722,
PITG_09431, PITG_18113, PITG_14609, PITG_09627, PITG_00558, PITG_17748,

PITG_09628, PITG_20264, PITG_19121, PITG_09524, PITG_05354, PITG_05174, PITG_07823, PITG_00388, PITG_19999, PITG_10863, PITG_03915, PITG_13763, PITG_17437, PITG_20209

GO:0009059
PITG_09866, PITG_20798, PITG_15393, PITG_09521, PITG_03178, PITG_12961, PITG_02493, PITG_09304, PITG_12947, PITG_12454, PITG_15090, PITG_22020, PITG_18518, PITG_22523, PITG_20689, PITG_03274, PITG_16198, PITG_20116, PITG_03661, PITG_00879, PITG_19007, PITG_18054, PITG_10210, PITG_14608, PITG_03221, PITG_09525, PITG_14850, PITG_18053, PITG_15566, PITG_12697, PITG_06596, PITG_10263, PITG_03398, PITG_20118, PITG_21393, PITG_06722, PITG_09631, PITG_20824, PITG_09431, PITG_18255, PITG_18258, PITG_20211, PITG_14609, PITG_03353, PITG_09627, PITG_17748, PITG_03916, PITG_20264, PITG_19121, PITG_13721, PITG_05354, PITG_05174, PITG_00388, PITG_07173, PITG_10863, PITG_19999, PITG_03915, PITG_07269, PITG_10193

GO:0044237
PITG_22577, PITG_21117, PITG_06929, PITG_02740, PITG_20156, PITG_16691, PITG_17745, PITG_03694, PITG_12454, PITG_07098, PITG_12725, PITG_03274, PITG_02852, PITG_17673, PITG_17516, PITG_17166, PITG_18634, PITG_18437, PITG_19488, PITG_14497, PITG_19607, PITG_03398, PITG_03440, PITG_09631, PITG_02461, PITG_21908, PITG_00571, PITG_02663, PITG_13792, PITG_13265, PITG_13719, PITG_12863, PITG_05990, PITG_00146, PITG_10929, PITG_15053, PITG_04708, PITG_16137, PITG_17128, PITG_15735, PITG_12996, PITG_09521, PITG_21185, PITG_18518, PITG_10399, PITG_18633, PITG_22523, PITG_05225, PITG_12105, PITG_06698, PITG_19362, PITG_16339, PITG_17993, PITG_07024, PITG_00330, PITG_17897, PITG_03332, PITG_03855, PITG_00989, PITG_12166, PITG_12053, PITG_03020, PITG_12697, PITG_21393, PITG_09431, PITG_20211, PITG_14609, PITG_15100, PITG_18435, PITG_20264, PITG_16527, PITG_19121, PITG_07823, PITG_02446, PITG_03365, PITG_17437, PITG_13074, PITG_06668, PITG_03934, PITG_19886, PITG_22124, PITG_07399, PITG_15090, PITG_20689, PITG_16138, PITG_10711, PITG_07962, PITG_04065, PITG_02697, PITG_02305, PITG_07349, PITG_10210, PITG_09092, PITG_19868, PITG_09415, PITG_09525,

PITG_06595, PITG_15529, PITG_13775, PITG_17289, PITG_14988, PITG_10270, PITG_03110, PITG_21557, PITG_06693, PITG_13416, PITG_10289, PITG_20759, PITG_12121, PITG_09596, PITG_07173, PITG_13761, PITG_04295, PITG_07269, PITG_02162, PITG_13441, PITG_18045, PITG_06429, PITG_12160, PITG_09304, PITG_12947, PITG_22020, PITG_19869, PITG_09824, PITG_00879, PITG_12818, PITG_13150, PITG_04701, PITG_10239, PITG_05594, PITG_13196, PITG_03221, PITG_16017, PITG_15566, PITG_06596, PITG_03262, PITG_09987, PITG_19537, PITG_20118, PITG_16113, PITG_04715, PITG_18255, PITG_03898, PITG_17330, PITG_07145, PITG_03864, PITG_00254, PITG_13164, PITG_14195, PITG_05354, PITG_02124, PITG_05523, PITG_09698, PITG_19999, PITG_03915, PITG_14639, PITG_13763, PITG_20798, PITG_06687, PITG_03178, PITG_12961, PITG_17839, PITG_04663, PITG_21077, PITG_12743, PITG_16198, PITG_05586, PITG_19132, PITG_06587, PITG_05850, PITG_07022, PITG_03773, PITG_05853, PITG_17253, PITG_14818, PITG_06814, PITG_09635, PITG_03738, PITG_10263, PITG_14463, PITG_05632, PITG_00594, PITG_20103, PITG_00988, PITG_13909, PITG_03353, PITG_04910, PITG_03916, PITG_09576, PITG_20195, PITG_21504, PITG_05127, PITG_07174, PITG_02493, PITG_18706, PITG_16056, PITG_17983, PITG_17165, PITG_00655, PITG_13905, PITG_19128, PITG_02455, PITG_15885, PITG_18853, PITG_17638, PITG_03441, PITG_07153, PITG_14608, PITG_06847, PITG_10444, PITG_09405, PITG_05522, PITG_06722, PITG_18113, PITG_00558, PITG_09628, PITG_13586, PITG_05174, PITG_09524, PITG_20209, PITG_17070, PITG_05314, PITG_17706, PITG_15393, PITG_17395, PITG_14920, PITG_17114, PITG_13165, PITG_06195, PITG_20116, PITG_05851, PITG_00407, PITG_05521, PITG_02497, PITG_19535, PITG_10998, PITG_18053, PITG_14156, PITG_03220, PITG_02561, PITG_07960, PITG_18891, PITG_20824, PITG_22685, PITG_14750, PITG_05512, PITG_13908, PITG_15598, PITG_09305, PITG_18977, PITG_13749, PITG_04858, PITG_05537, PITG_13778, PITG_10193, PITG_07308, PITG_00051, PITG_17021, PITG_09866, PITG_03150, PITG_20141, PITG_20560, PITG_02467, PITG_05585, PITG_16526, PITG_00187, PITG_00289, PITG_07099, PITG_03905, PITG_02560, PITG_03661, PITG_19007, PITG_15705, PITG_18054, PITG_07916, PITG_06817, PITG_00474, PITG_13415, PITG_06889, PITG_14850, PITG_05762, PITG_00471, PITG_02527, PITG_02026, PITG_18258, PITG_09627, PITG_17748, PITG_06274, PITG_00388, PITG_10863, PITG_17400, PITG_00588

GO:0006729
PITG_05851, PITG_05853, PITG_13586, PITG_02663, PITG_05850

GO:0046146
PITG_05851, PITG_05853, PITG_13586, PITG_02663, PITG_05850

GO:0044238
PITG_22577, PITG_21117, PITG_02740, PITG_20156, PITG_16691, PITG_07098, PITG_12454, PITG_03274, PITG_12725, PITG_02852, PITG_17673, PITG_17516, PITG_17166, PITG_18634, PITG_19488, PITG_18437, PITG_14497, PITG_03398, PITG_03440, PITG_09631, PITG_02461, PITG_21908, PITG_00571, PITG_13792, PITG_13265, PITG_13719, PITG_05990, PITG_10929, PITG_00146, PITG_15053, PITG_16137, PITG_17128, PITG_15735, PITG_12996, PITG_09521, PITG_21185, PITG_18518, PITG_10399, PITG_18633, PITG_22523, PITG_05225, PITG_12105, PITG_06698, PITG_19362, PITG_16339, PITG_07024, PITG_00330, PITG_17897, PITG_03855, PITG_12166, PITG_12053, PITG_03020, PITG_12697, PITG_21393, PITG_09431, PITG_20211, PITG_14609, PITG_18435, PITG_15100, PITG_16527, PITG_20264, PITG_19121, PITG_02446, PITG_07823, PITG_17437, PITG_13074, PITG_06668, PITG_03934, PITG_22124, PITG_07399, PITG_15090, PITG_20689, PITG_16138, PITG_10711, PITG_04065, PITG_02697, PITG_02305, PITG_07349, PITG_17592, PITG_10210, PITG_09092, PITG_19868, PITG_09525, PITG_06595, PITG_15529, PITG_13775, PITG_17289, PITG_14988, PITG_10270, PITG_03110, PITG_07384, PITG_21557, PITG_06693, PITG_10289, PITG_20759, PITG_12121, PITG_09596, PITG_07173, PITG_13761, PITG_04295, PITG_07269, PITG_02162, PITG_18045, PITG_06429, PITG_12160, PITG_09304, PITG_12947, PITG_22020, PITG_19869, PITG_09824, PITG_00879, PITG_12818, PITG_05594, PITG_10239, PITG_04701, PITG_03221, PITG_13196, PITG_09474, PITG_16017, PITG_15566, PITG_06596, PITG_09987, PITG_20118, PITG_19537, PITG_16113, PITG_18255, PITG_03898, PITG_07145, PITG_00254, PITG_13164, PITG_14195, PITG_13721, PITG_05354, PITG_02124, PITG_05523, PITG_09698, PITG_19999, PITG_03915, PITG_14639, PITG_13763, PITG_20798, PITG_06687, PITG_03178, PITG_12961, PITG_13483, PITG_14717, PITG_04663, PITG_21077, PITG_12743, PITG_16198,

	PITG_05586, PITG_00229, PITG_19132, PITG_06587, PITG_07022, PITG_03773, PITG_14818, PITG_09635, PITG_03738, PITG_10263, PITG_13722, PITG_14463, PITG_05632, PITG_00594, PITG_20103, PITG_00988, PITG_03353, PITG_04910, PITG_03916, PITG_09576, PITG_20195, PITG_21504, PITG_05127, PITG_07174, PITG_02493, PITG_18706, PITG_16056, PITG_17983, PITG_17165, PITG_00655, PITG_13905, PITG_19128, PITG_02679, PITG_02455, PITG_15885, PITG_18853, PITG_17638, PITG_16991, PITG_03441, PITG_07153, PITG_14608, PITG_06847, PITG_10444, PITG_09405, PITG_05522, PITG_06722, PITG_18113, PITG_00558, PITG_09628, PITG_09524, PITG_05174, PITG_20209, PITG_17070, PITG_05314, PITG_17706, PITG_15393, PITG_17114, PITG_13165, PITG_06195, PITG_20116, PITG_00407, PITG_05521, PITG_02497, PITG_19535, PITG_10998, PITG_18053, PITG_14156, PITG_03220, PITG_02561, PITG_07960, PITG_18891, PITG_20824, PITG_22685, PITG_14750, PITG_05512, PITG_15598, PITG_13908, PITG_09305, PITG_18977, PITG_13749, PITG_04858, PITG_05537, PITG_13778, PITG_02766, PITG_10193, PITG_07308, PITG_00051, PITG_17021, PITG_09866, PITG_20141, PITG_03150, PITG_05585, PITG_02467, PITG_20560, PITG_00187, PITG_16526, PITG_00289, PITG_03905, PITG_07099, PITG_03661, PITG_19007, PITG_15705, PITG_18054, PITG_07916, PITG_06817, PITG_21841, PITG_13415, PITG_06889, PITG_14850, PITG_05762, PITG_02527, PITG_02026, PITG_18258, PITG_09627, PITG_17748, PITG_06274, PITG_00388, PITG_10863, PITG_17400, PITG_00588
	GO:0003735<br>PITG_20798, PITG_09521, PITG_03178, PITG_15090, PITG_12947, PITG_16198, PITG_20116, PITG_00879, PITG_19007, PITG_18054, PITG_14608, PITG_03221, PITG_09525, PITG_14850, PITG_12697, PITG_06596, PITG_10263, PITG_20118, PITG_09631, PITG_09431, PITG_03353, PITG_09627, PITG_03916, PITG_20264, PITG_19121, PITG_05174, PITG_00388, PITG_07173, PITG_10863, PITG_19999, PITG_07269, PITG_10193
Under	None
